# Unconventional isoquinoline-based SERMs elicit fulvestrant-like transcriptional programs in ER+ breast cancer cells

**DOI:** 10.1038/s41523-022-00497-9

**Published:** 2022-12-14

**Authors:** G. R. Hancock, K. S. Young, D. J. Hosfield, C. Joiner, E. A. Sullivan, Y. Yildiz, M. Lainé, G. L. Greene, S. W. Fanning

**Affiliations:** 1 Department of Cancer Biology, Loyola University Chicago, Stritch School of Medicine, Maywood, IL 60153 USA; 2 Ben May Department for Cancer Research, University of Chicago, Chicago, IL 60637 USA

**Keywords:** Breast cancer, Drug discovery and development, Breast cancer, Drug development

## Abstract

Estrogen receptor alpha (ERα) is a ligand-dependent master transcriptional regulator and key driver of breast cancer pathology. Small molecule hormones and competitive antagonists favor unique ERα conformational ensembles that elicit ligand-specific transcriptional programs in breast cancer and other hormone-responsive tissues. By affecting disparate ligand binding domain structural features, unconventional ligand scaffolds can redirect ERα genomic binding patterns to engage novel therapeutic transcriptional programs. To improve our understanding of these ERα structure-transcriptional relationships, we develop a series of chemically unconventional antagonists based on the antiestrogens elacestrant and lasofoxifene. High-resolution x-ray co-crystal structures show that these molecules affect both classical and unique structural motifs within the ERα ligand binding pocket. They show moderately reduced antagonistic potencies on ERα genomic activities but are effective anti-proliferative agents in luminal breast cancer cells. Interestingly, they favor a 4-hydroxytamoxifen-like accumulation of ERα in breast cancer cells but lack uterotrophic activities in an endometrial cell line. Importantly, RNA sequencing shows that the lead molecules engage transcriptional pathways similar to the selective estrogen receptor degrader fulvestrant. This advance shows that fulvestrant-like genomic activities can be achieved without affecting ERα accumulation in breast cancer cells.

## Introduction

Breast cancer is the most diagnosed and second leading cause of cancer-related death in women worldwide^1,2^. Estrogen receptor alpha-positive (ERα+) luminal subtype is the most prevalent, accounting for approximately 70% of breast cancers^3^. Although targeted therapies have extended life, ERα+ patients face relapse and drug resistance with current therapies due to multiple mechanisms of acquired and de novo resistance. Many of these resistant diseases retain ERα-dependence and some degree of sensitivity to next-generation antiestrogens with improved antagonistic potencies^4,5^. While these molecules show improved progression-free survival compared to standard of care^6^, many ER+ patients fail to respond. As these patients retain ERα expression, new ways of targeting ERα could further improve anti-cancer efficacies in this setting.

Most ERα+ breast cancer patients will receive 5 years of first-line adjuvant hormone therapy, often in conjunction with CDK4/6 (palbociclib/ribociclib/abemaciclib), mTORC1 (everolimus), or PI3K (alpelisib) inhibitors^7^. Aromatase inhibitors (AIs) are a common post-menopausal first-line therapy used to ablate estrogen levels via estradiol synthesis inhibition. However, bone loss and musculoskeletal side effects reduce patient compliance^8,9^. Tamoxifen, a selective estrogen receptor modulator (SERM), is primarily administered to pre-menopausal breast cancer patients in combination with ovarian suppression^10^. This SERM antagonizes ERα in breast cancer cells while acting as a partial agonist in the bone and uterine epithelium. While the bone-sparing activities of tamoxifen are favorable, its uterotrophic “SERM-agonist” activities increase the risk of endometrial cancers and are linked to mechanisms of therapeutic resistance^11^.

The selective estrogen receptor degrader (SERD) fulvestrant (ICI) is a second-line hormone therapy, typically given after tamoxifen or AI therapy failure^12,13^. While ICI antagonizes ERα transcription like tamoxifen, it differs by inducing receptor proteasomal degradation and unique transcriptomic effects^14,15^. These ER-degrading activities have previously been linked to ICI’s complete antagonism in hormone-responsive tissues. However, its pharmaceutical shortcomings, namely poor solubility, reduce its therapeutic utility^16^. In addition, evaluations of ICI’s mechanism of therapeutic action by the McDonnell laboratory have shown that transcriptional antagonism rather than potent and effective ERα degradation drive efficacy^14,17^. Mixed SERM/SERDs that show reduced or no ERα degradation including lasofoxifene (laso) and bazedoxifene, demonstrate superior tissue-specific activities. They are potent antagonists in the breast, partial agonists in the bone, and show few activities in other tissues^18,19^.

**Fig. 1:**
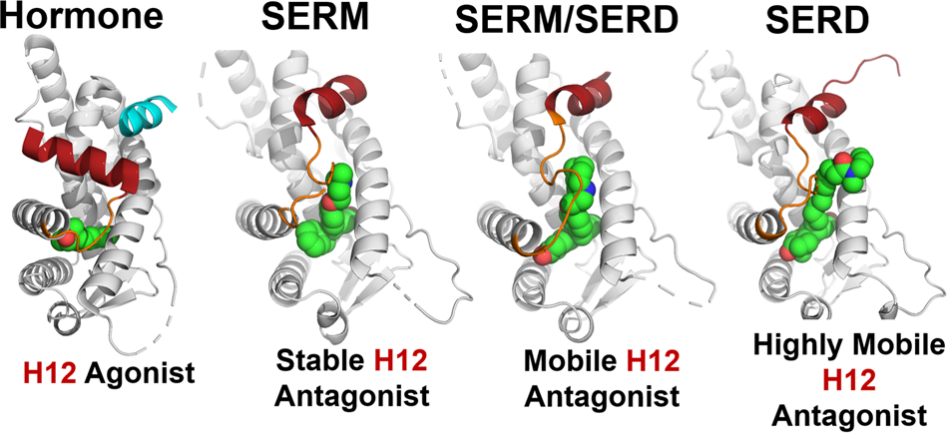
Structural basis of ligand-specific ERα activities. Ligands are shown as spheres with carbon in green, oxygen in red, and nitrogen in blue, helix 12 is colored red, the helix 11–12 loop is colored orange, and coregulator peptide in cyan. PDBs: 1GWR, 5W9C, 4XI3, and 7R62.

ERα transcriptional activities ultimately depend on the formation of ligand-specific transcriptional coregulator complexes^20,21^. The ERα ligand binding domain (LBD) is a bundle of 12 alpha helices. Hormone binding favors LBD head-to-head homodimerization, helix 12 (H12) binding over the hormone-binding pocket, and exposure of the activating function-2 (AF-2) cleft where transcriptional coregulators bind via LXXLL motifs. SERMs and SERDs are comprised of a heterocyclic core that mimics hormone binding and a side-arm that disfavors the H12 agonist conformation. Rather, H12 packs into the AF-2 cleft via an LXXML motif to sterically preclude transcriptional coregulator binding (Fig. 1). SERDs induce ERα degradation by affecting the conformational dynamics of the loop connecting H11 and H12, which further destabilizes H12^22^. SERM/SERDs show lesser effects on the conformational dynamics of the H11-12 loop and H12, which correlates to their reduced ERα degrading activities^19,22^.

**Fig. 2:**
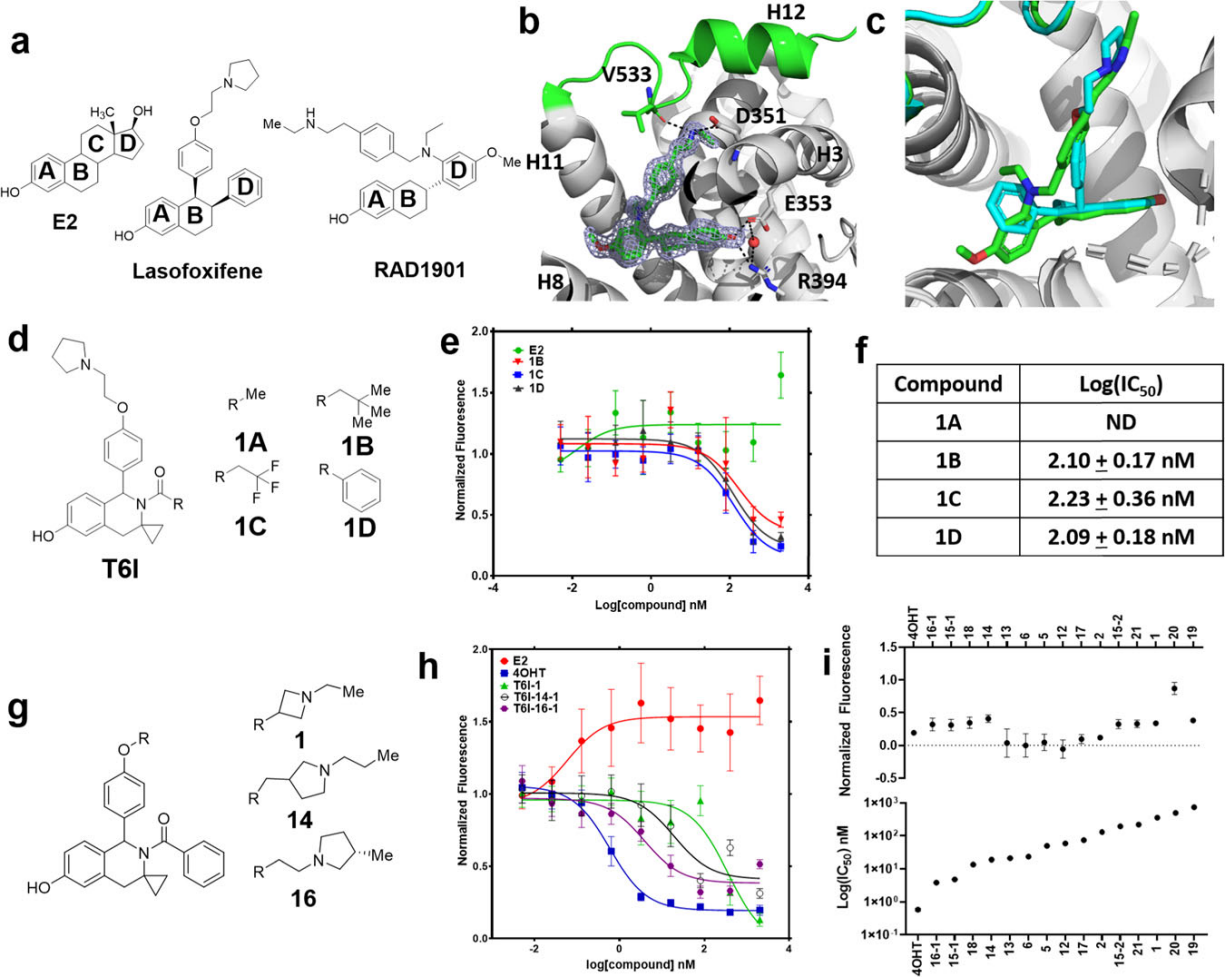
Structural basis of RAD1901 ERα antagonism and design of novel T6I-based antiestrogens. **a** Chemical structures of 17β-estradiol (E2), laso, and RAD1901. **b** X-ray co-crystal structure of RAD1901 bound in the hormone binding pocket. Blue mesh shows measured 2mFo-DFc difference density contoured to 1.5 σ. **c** Superposition of RAD1901 (green) and laso (cyan) x-ray co-crystal structures based on alpha carbon positions. Protein DataBank (PDB) Accession Numbers: 6VJD and 7TE7. **d** Chemical structures of initial T6Is. **e** ERE reporter gene assay of ERα transcriptional activation in MCF7 cells normalized to cell count. **f** Summary of Log(IC_50_) for each compound. **g** Chemical structure of representative second generation T6Is. **h** Representative reporter gene data in MCF7 cells. **i** Summary of normalized fluorescence at maximum dose (1 μM) and Log(IC_50_). All treatments are shown as the mean of three replicates ± standard deviation (s.d.). All data are normalized to cell count in their respective wells. All antagonist treatments were performed in the presence of 1 nM E2. Error bars represent s.d.

Elacestrant (RAD1901) is a chemically distinctive antiestrogen. SERD side arms are typically attached to the core at the analogous steroidal B-ring. However, RAD1901’s side-arm attaches at the analogous D-ring (Fig. 2a). It demonstrates a SERM-like effect on ERα accumulation in breast cancer cells at lower doses and a SERD-like effect on the receptor at higher doses^23^. RAD1901 is orally available and is effective against wild type and mutant ER in both in vitro and in vivo models^24–26^. In a recent Phase III clinical trial in advanced disease with prior hormone therapy, RAD1901 reduced the risk of disease progression or death by 30% in all patients and 45% in patients with *ESR1* mutations, including the Y537S, when compared to standard of care^6^. The clinical success of RAD1901 highlights the potential utility of structurally unconventional ERα antagonists in the late-stage ER+ breast cancer setting.

By affecting different helices and loops near the hormone-binding pocket, unconventional SERMs and SERDs can alter the repertoire of ERα-associated transcriptional coregulators. The Nettles and Katzenellenbogen laboratories developed the novel oxabicycloheptene sulfonate (OBHS) antiestrogenic scaffold. It uniquely antagonizes ERα by directly perturbing H11 and in turn favors H12 packing in the AF-2 cleft^27^. Further modification to the OBHS scaffold resulted in an ICI-like complete antagonism^28^. Based on these studies, next-generation dual-mechanism estrogen receptor inhibitors (DMERIs) were developed. By disrupting unique structural motifs near the hormone-binding pocket, these DMERIs repopulated ERα-transcriptional coregulator interactions^29^. Together, the RAD1901, OBHS, and DMERI studies show how structurally unconventional SERMs and SERDs can be used to elicit new ERα anti-cancer activities in breast cancer cells.

In this study, we developed a series of isoquinoline-based SERMs and SERDs to understand how ligand manipulation of specific ERα structural motifs affects transcriptional programs in luminal breast cancer-relevant models. These molecules were designed based on an x-ray co-crystal structure we solved of RAD1901 and earlier studies with laso^18,30^. Optimization of side-arm composition yielded improved therapeutic anti-transcriptional activities. The scaffold favors a SERM-like accumulation of ERα despite the incorporation of side arms taken from next-generation SERDs. Parallel structural analysis shows a unique disruption of F425 in H8 in addition to interactions with helices 3, 11, and 12. The most effective molecules show similar anti-proliferative activities in breast cancer cells to other SERMs and SERDs. Despite their SERM-like profiles in breast cancer cells, they show reduced uterotrophic activities. RNAseq in T47D breast cancer cells shows similar transcriptional programs for the lead isoquinoline with RAD1901, laso, and especially ICI. However, unique effects on *CDK1* and especially *SUMO1* are also observed. Our findings show that chemical manipulation of unique structural motifs can engage novel ERα anti-breast cancer activities.

## Results

### RAD1901 adopts a unique ligand-binding pose

To understand whether the unconventional 2D chemical structure of RAD1901 resulted in a unique ERα ligand binding pose, we solved an x-ray co-crystal structure to 1.85 Å. Overall, this structure presents a canonical ERα LBD head-to-head homodimer in the asymmetric unit and RAD1901 is well ordered in the hormone-binding pocket (Fig. 2b). Rather than the T-shaped orientation typical of most SERMs and SERDs, RAD1901 adopts an acute L-shaped ligand binding pose. Its tetrahydrofuran core participates in a bifurcated hydrogen bond between E353, R394, and a water molecule similar to other SERMs and SERDs (Fig. 2b). The side-arm adopts a vector perpendicular to helix 11 (H11) and engages a bifurcated hydrogen bond between D351 and V533 to favor antagonistic H12 docking in the activating function-2 (AF-2) cleft. RAD1901 shares a similar tetrahydrofuran core to laso. Within their respective x-ray co-crystal structures, cores of laso and RAD1901 are superimposable, while the D-ring analog lies between H8 and H11 similar to structurally novel OBHS and DMERI SERMs and SERDs (Fig. 2c)^27,29,31^. Overall, this structure shows that RAD1901 adopts an unusual L-shaped ligand binding pose that is positioned near H3, H11, and H12. Because RAD1901 elicits novel ERα-dependent genomic activities^25^, this structure implies that unique ligand binding poses may contribute to new therapeutic transcriptional programs in breast cancer cells.

### Discovery of tetrahydro-6-isoquinoline-based SERMs and SERDs

To better understand the relationship between ligand binding pose and ERα anti-cancer activities, we developed a new antiestrogenic scaffold based on the RAD1901 and laso x-ray co-crystal structures. We chose a tetrahydro-6-isoquinoline (T6I) core to mimic the tetrahydrofuran of laso. A cyclopropyl group was used in place of RAD1901’s ethylamine. A pyrrolidine-containing side-arm was used to mimic laso^18,22,32,33^. We initially synthesized 4 derivatives comprised of different pharmacophores on the analogous steroidal A ring (Fig. 2d) and used an ERE reporter gene assay in MCF7 breast cancer cells to measure their transcriptional inhibitory activities^32^. All treatments were performed in triplicate in the presence of 1 nM E2 to stimulate ERα transcription. The benzamide derivative (T6I-1D) showed the best anti-transcriptional IC_50_ at approximately 100 nM (Fig. 2e, f).

Side arms affect anti-transcriptional activities and ERα lifetime based on how they manipulate the conformational dynamics of H12 and its preceding loop^20,22,31,34^. We synthesized 14 T6I analogs, based on existing SERMs and SERDs, to determine whether side-arm composition improved transcriptional inhibition (Supplementary Fig. 1). Figure 2g shows representative side arms. Those with increased bulkiness including methylpyrrolidine, benzyl, and azepans enhanced anti-transcriptional activities (Fig. 2h, i). Whereas, less bulky side arms (i.e. methylamine), showed markedly reduced anti-transcriptional potency. These molecules were synthesized as a mix of enantiomers. To understand whether one enantiomer accounted for activity, we separated the chiral species of a subset of T6Is. The first peak for each molecule showed a 10–100-fold improved anti-transcriptional IC_50_ over the second (Fig. 2i). Supplementary Fig. 2 shows dose-response curves for all the first 14 T6I derivatives in the reporter gene assay.

### T6Is favor SERM-like effects on ERα

**Fig. 3:**
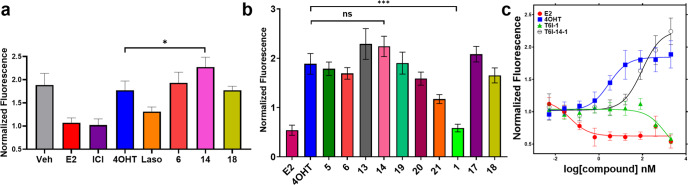
T6Is differentially affect ERα lifetime in breast cancer cells. **a** In-cell Western of endogenous ERα levels normalized to cell count in T47D breast cancer cells. **b** Summary of halo-ERα accumulation at maximum dose (1 μM). **c** Representative dose-response of halo-ERα expression in T47D breast cancer cells, data are normalized to cell count in their respective wells. All data are shown as the mean of three replicates ± s.d., ns = not significant, *p < 0.05, ***p < 0.0005 by unpaired *t* test. Error bars represent s.d.

SERMs and SERDs differentially affect ERα cellular lifetime/accumulation, which correlates with tissue-specific partial agonistic activities. SERMs increase WT ERα lifetime and are partial agonists in the bone and uterine epithelium by maintaining AF-1-dependent transcriptional coactivator recruitment^35^. SERDs induce rapid WT ERα degradation and are pure antagonists^14,17,19,22,33^. Figure 3 shows the impact of T6Is on ERα cellular lifetime in breast cancer cells. We used an in-cell Western approach to measure how a subset of T6Is affected ERα levels in MCF7:WS8 breast cancer cells. Cells were serum starved for 48 h then treated with 1 μM T6I alongside E2 (hormone), ICI (SERD), 4OHT (SERM), and laso (SERM/SERD) for 24 h. Each T6I increased ERα levels similar to the SERM 4OHT with T6I-14 showing a significant enhancement at *p*-value = 0.04 by *t* test (Fig. 3a). A live cell halo-tagged ERα assay was then used to measure the impact of an expanded set of T6Is on receptor accumulation in T47D breast cancer cells after 24 h treatment^32^. Despite many of the side arms originating from SERDs (i.e. the azepan of T6I-6 is present in bazedoxifene^19^), most T6Is demonstrated SERM-like increases to ERα at 1 μM dose. The difference between T6I-14 and 4OHT was not statistically significant in this assay. T6I-1 was the only molecule to show a SERD-like decrease to ERα levels that were significant (*p* = 0.0004) compared to 4OHT (Fig. 3b). We also measured dose-response using this system and Fig. 3c shows measured fluorescence across the 5 pM to 1 μM dose range. The T6Is showed a broad range of IC_50_ values from 34.98 ± 0.07 nM to >1 μM (Supplementary Fig. 3). The IC_50_ values generally trended with observed differences in transcriptional IC_50_ (Fig. 2), while the effect at maximum dose varied greatly. Together these data show that the T6I scaffold favors a SERM-like accumulation of ERα in the breast cancer cell.

### T6Is inhibit ERα coactivator binding

**Fig. 4:**
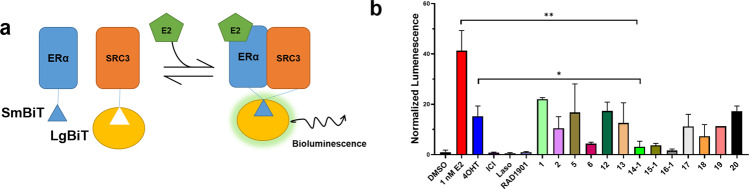
Impact of T6Is on ERα-SRC3 complex formation in HEK293T cells. **a** Schematic of nanoBiT assay. **b** Impact of hormone, SERM, and SERD on ERα-SRC3 binding after 4 h. Data are the mean of three replicates ± s.d. All antagonist treatments were performed at 1 μM in the presence of 1 nM E2. Significance determined by unpaired *t* test where *p < 0.05, **p < 0.005. Error bars represent s.d.

SERMs and SERDs favor an ERα LBD protein conformation that occludes transcriptional coactivator binding^20^. We used nanoBiT to study how the first set of T6I molecules inhibited ERα and steroid receptor coactivator-3 (SRC3) binding in HEK293T cells^36^. Figure 4 shows a schematic of the NanoBiT assay and results from this study. Plasmids for smBiT-ERα and lgBiT-SRC3 were kindly donated by Dr. Donald P. McDonnell. Plasmids were co-transfected into HEK293T cells and placed in serum-starved medium for 48 h. Cells were treated with vehicle (DMSO), 1 nM E2, or 1 nM E2 + 1 μM antagonist for 4 h then read for luminescence. Treatment of 1 nM E2-alone lead to a 40-fold increased signal compared to veh, which was antagonized in the presence of all SERMs and SERDs. T6Is 14-1, 15-1, and 16-1 were among the most potent while 1 and 2 were the least. T6I-14-1 showed a significant reduction compared to both 4OHT (*p* = 0.03) and E2 (*p* = 0.004). Overall, these findings suggest a relationship between the inhibition of coactivator binding and the efficacy ERα transcriptional antagonistic activities.

### T6Is effectively reduce breast cancer cellular proliferation

**Fig. 5:**
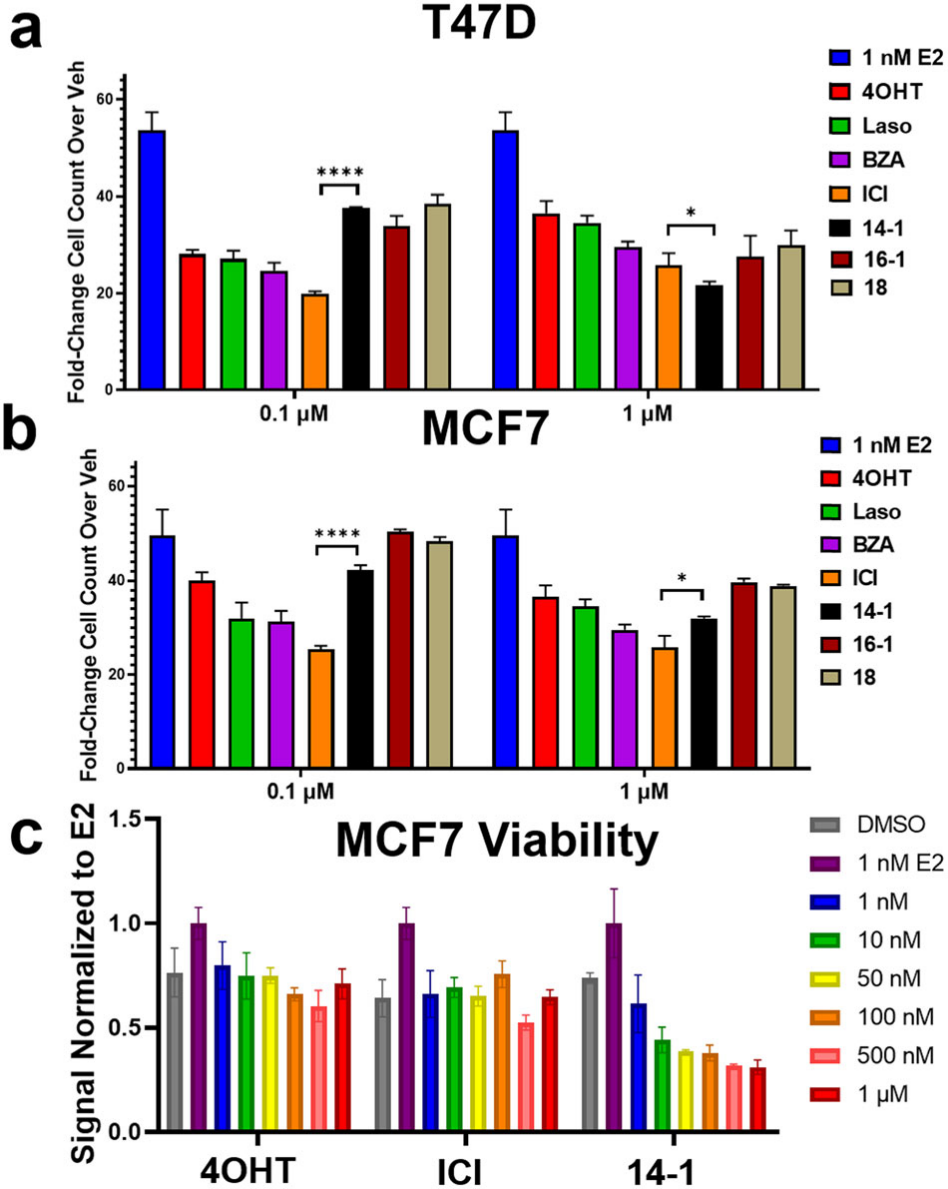
T6Is are effective anti-proliferative agents in ER+, hormone-dependent, breast cancer cells. T47D (**a**) and MCF7 (**b**) cell count after 7 days of treatment in the presence of 1 nM E2, data are the mean ± s.d. for three replicates normalized to vehicle. **c** Crystal violet viability assay of MCF7 breast cancer cells treated in the presence of 1 nM E2, data are the mean of 3 replicates ± s.d. and are normalized to 1 nM E2. Significance determined by unpaired *t* test where *p < 0.05, ****p < 0.00005. Error bars represent s.d.

We next measured the abilities of T6Is 14-1, 16-1, and 18 to inhibit the proliferation of T47D and MCF7 breast cancer cells alongside 4OHT, laso, bazedoxifene (BZA), and fulvestrant (ICI) using label-free cell counting. Cells were treated with 0.1 and 1 μM SERM or SERD for 7 days in the presence of 1 nM E2. Figure 5 shows the fold-change in cell count normalized to vehicle (DMSO) after normalizing to starting cell count in each well. The T6Is showed a reduced inhibition at 0.1 μM but similar effects at 1 μM (Fig. 5a, b). Although, these changes appeared slightly improved in T47D cells compared to MCF7 where T6I-14-1 was significantly more effective (*p* = 0.01) than ICI. While, ICI was significantly (*p* = 0.02) more effective at the higher dose in the MCF7 cells. In addition, we used a crystal violet assay to measure the effect of T6I-14-1 on MCF7 viability^37^. Cells were treated with 4OHT, ICI, or T6I-14-1 at 1 to 1000 nM in the presence of 1 nM E2 for 7 days. Surprisingly, T6I-14-1 showed reduced MCF7 cellular viability across all doses compared to 4OHT and ICI in this assay (Fig. 5c).

### T6I core uniquely perturbs Helix 8

**Fig. 6:**
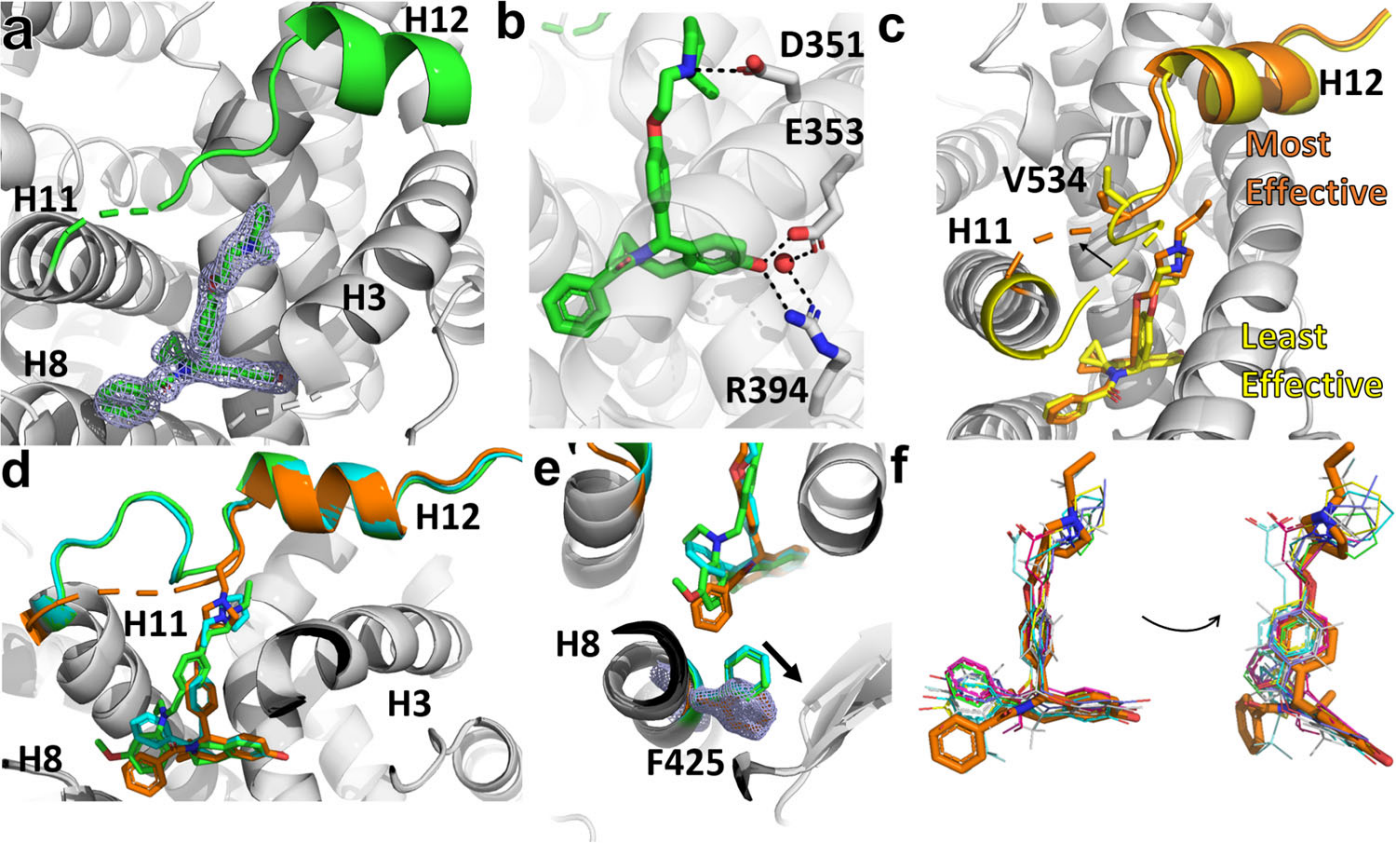
T6Is adopt a unique pose in the ERα hormone-binding pocket. **a** 2mFo-DFc difference density map (blue mesh) of T6I-18 (green sticks) contoured to 1.5 σ. H11-12 loop and H12 are highlighted in green. **b** T6Is participate in common hydrogen bond patterns to other hormones, SERMs, and SERDs. Hydrogen bonds are shown as dashed lines. **c** Superposition of T6I-1 and 14-1 x-ray co-crystal structures showing that anti-transcriptional efficacies correspond to side-arm positioning near the H11-12 loop at H12. **d** Superposition of T6I-14-1 (orange), lasofoxifene (cyan), and RAD1901 (green) x-ray crystal structures based on alpha carbon positions. **e** F425 movement in the T6I structures, blue mesh is a 2mFo-DFc difference density map of F425 in the T6I-14-1 structure contoured to 1.5 σ. **f** Superposition representative orally available SERM and SERD ligand binding poses in the hormone binding pocket. PDBs: 1RK5, 4XI3, 5AAC, 5UFX, 5W9C, 6B0F, 6PFM, 6VJD, 7KCA, 7TE7, and 8DUD.

We solved x-ray co-crystal structures to reveal how the T6Is bound in the ERα hormone binding pocket and to understand the structural basis of differential efficacy. We were able to solve structures for 10 of the 14 T6Is. Figure 6 shows x-ray crystallographic analysis and comparison to known SERMs and SERDs. In these structures, the T6Is are well ordered in the ligand binding pocket (Fig. 6a) and form canonical ERα LBD antagonist conformations with H12 packed in the AF-2 cleft. Hydrogen bonds are formed between the T6I core, E353, R394, and a water molecule while the side arm forms a hydrogen bond with D351 to favor an antagonistic H12 conformation that is docked into the AF2-cleft (Fig. 6b). The T6I core is superimposable between the 10 structures but the side arms adopt divergent vectors near H12 that correlate with ligand-specific antagonistic differences (Supplementary Fig. 4). Those with the greatest anti-transcriptional efficacies, such as T6I-14-1 perturb the H11-12 loop near V534 and sit closer to H12, whereas the least effective molecules (i.e. T6I-1) sit away from the H11-12 loop and do not approach H12 (Fig. 6c). Crystal contacts are frequently formed near the H11-12 loop by crystal symmetry mates but none were observed in these structures^30^.

The T6I core superimposes with the tetrahydrofuran of laso and RAD1901 near E353 while its benzamide adopts a similar orientation to RAD1901 but lies closer to H8 (Fig. 6d). The T6I side-arm adopts an identical vector in the ligand binding pocket to laso. It should be noted that the H11-12 loop is affected by crystal contacts in the laso and RAD1901 structures confounding analysis. Interestingly, the F425 in H8 is rotated away from the ligand pocket in the T6I structures compared to laso and RAD1901 (Fig. 6e). While this F425 movement should explain the reduced potency, our initial structure–activity relationship (SAR) study contradicts this conclusion, where the benzamide and bulkier groups were preferred (Fig. 2d–f). When compared to a broader set of SERMs and SERDs, the benzamide of the T6I core is uniquely oriented near H8 while the H3 and H12-facing substituents adopt similar orientations (Fig. 6f). Together, these structures show that the T6I scaffold adopts a unique H3-8-11-12 ligand binding pose compared to other SERMs and SERDs.

### Improving of T6I anti-proliferative activities

**Fig. 7:**
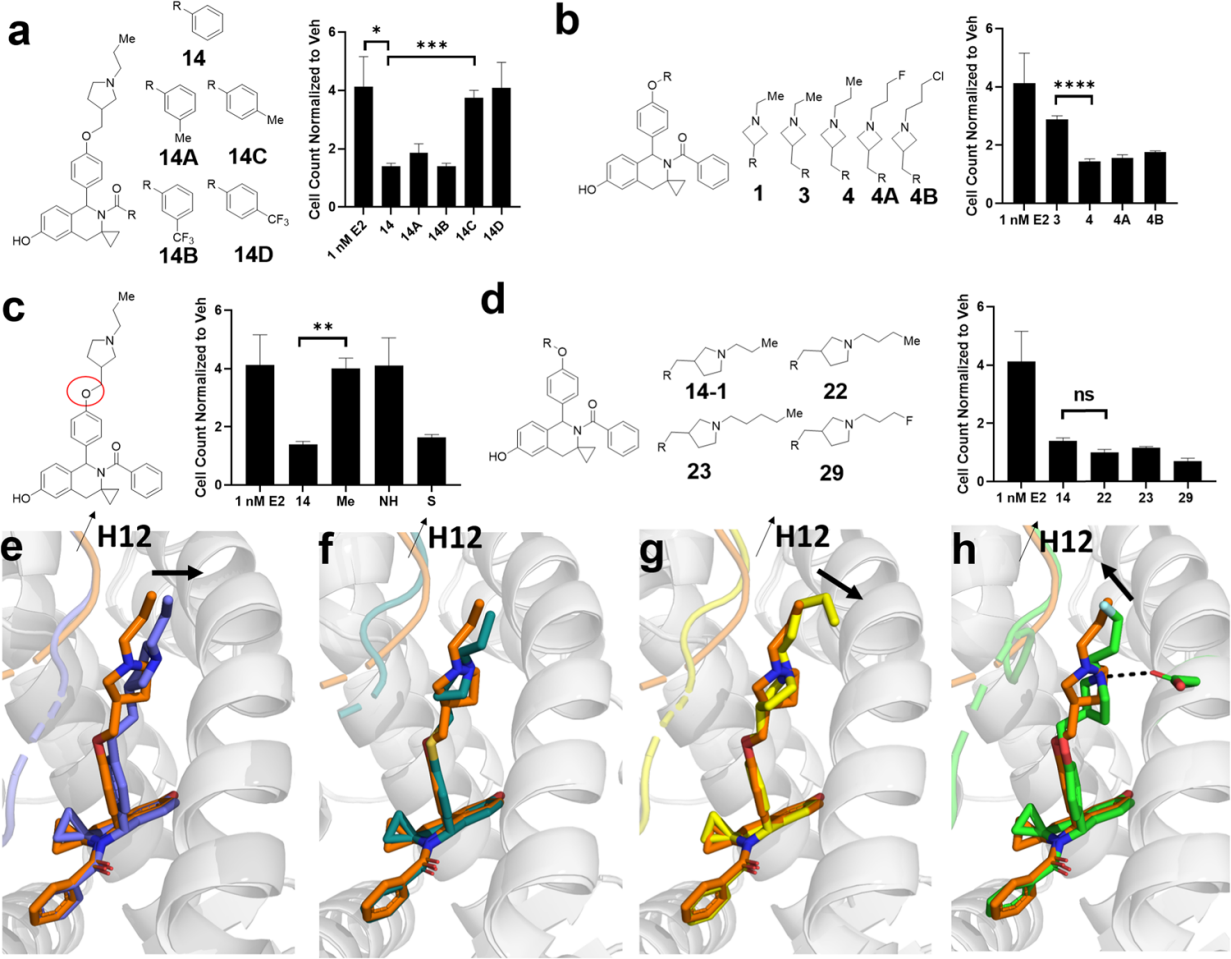
Structure–activity relationships to improve T6I anti-proliferative efficacies. **a** Derivatives of the benzamide moiety. **b** Azetidine derivatives. **c** Core-arm linker atom from an oxygen to carbon (Me), nitrogen (NH), or thiol (S). **d** Pyrrolidine derivatives. All column graphs are the cell count of T47D breast cancer cells after 7 days in the presence of 1 nM E2. Data shown are the mean of three replicates ± s.d. normalized to vehicle (DMSO) control. Error bars represent s.d. Significance determined by unpaired *t* test where *p < 0.05, **p < 0.005, ***p < 0.0005, ****p < 0.00005, ns = not significant. Superposition based on alpha carbon positions of T6I 14-1 (orange) with (**e**) T6I-Me, (**f**) T6I-27, (**g**) T6I-23, (**h**) and T6I-29. Arrows indicate repositioning compared to T6I-14. PDBs: 8DV7, 8DV8, 8DV5, and 8DVB.

The most effective T6Is lacked potency compared to other SERMs and SERDs. To improve potency, we examined SARs on three T6I sites. Figure 7 shows the chemical structures, anti-proliferative activities, and x-ray crystal structures of the second-generation T6Is.

*Site 1: Benzamide near Helix 8*. To determine what pharmacophores could be accommodated near H8, we synthesized meta, para-methyl and -trifluoromethyl derivatives of T6I-14-1. Both meta-substituted molecules showed no improvement on anti-proliferative activities in T47D breast cancer cells after 7 days and para substitutions inactivated the molecule (Fig. 7a). This is not surprising as the para carbon is already in close proximity to H8 in the x-ray crystal structure and there is insufficient room to accommodate substitutions at this site.

*Site 2: Azetidine SERD (T6I-1)*. One goal of this study was to develop structurally distinct SERMs as well as SERDs. While the majority of T6Is showed SERM-like ERα accumulation in breast cancer cells, only T6I-1 demonstrated weak ERα-degrading activities. However, it was an ineffective anti-transcriptional agent. The crystal structure showed that the azetidine could not hydrogen bond with D351, a common interaction used by SERMs and SERDs (Fig. 6c). As such, we synthesized derivatives of T6I-1 to determine whether increased linker length could improve the anti-transcriptional potency of the azetidine side-arm by capturing the D351 hydrogen bond. Increasing linker length alone did not improve anti-proliferative activities (T6I-3), but converting it to a propyl instead of ethylazetidine significantly enhanced antagonism (Fig. 7b). We also synthesized fluoro and chloropropyl analogs and found no change to efficacies. We used an in-cell Western approach to determine whether ERα-degrading activities were maintained. Surprisingly, T6I-4 showed a similar SERM-like enrichment of ERα in T47D breast cancer cells (Supplementary Fig. 5).

*Site 3: Alkyl Pyrrolidine Side Arm (T6I-14-1)*. In the first x-ray crystal structures, T6Is that adopted ligand binding poses with side arm vectors placed close to the H11-12 loop and H12 showed improved anti-transcriptional and proliferative efficacies. To explore the role of side arm vector, we changed the side arm-core linker to alkyl, amino, or thiol (Fig. 7c). Substitution to alkyl and amino groups neutralized anti-proliferative activities, while the thiol maintained a similar activity to the ether parent compound. In parallel, we studied whether increasing the alkyl group length off the pyrrolidine could improve potency (Fig. 7d). No effect was observed when butyl or pentyl alkyl groups were included. However, substitution with a fluoropropyl (T6I-29) showed a slightly improved efficacy versus propyl alone. It should be noted that we previously reduced alkyl length to an ethyl with T6I-13-1 and observed reduced efficacy compared to the propyl of T6I-14-1.

X-ray co-crystal structure analysis was performed to determine how differences in T6I side arm composition affected the antagonistic ligand binding pose (Fig. 7e–h). We were unable to solve structures with the benzamide substitutions or the azetidine derivatives. For the methyl-linked T6I-14-1 derivative, it adopted a side arm vector away from H12 suggesting a reduced ability to enforce the H12 antagonistic conformation (Fig. 7e). As no change in activity was observed, it was not surprising that the thiol-linked molecule showed a similar binding pose to the ether-linked parent compound (Fig. 7f). For the derivatives with increased alkyl length, additional terminal carbons pointed away from H12 and towards solvent suggesting no benefit to increased length (Fig. 7g). Interestingly, the fluoropropyl (T6I-29) showed a rotated pyrrolidine that improved the hydrogen bonding angle with D351 (Fig. 7h). In turn, the fluoro group sat closer to the H11-12 loop and H12 suggesting a better ability to favor the antagonistic H12 conformation.

#### The effect of T6I-29 enantiomers on ERα breast cancer cellular activities

**Fig. 8:**
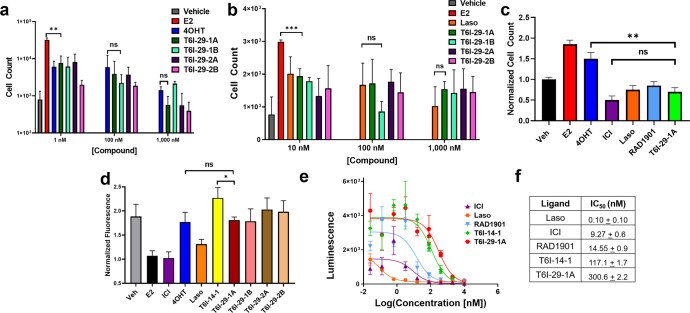
Effect of T6I-29 on ERα breast cancer activities. **a** Cell count after 7 days treatment of the separated T6I-29 peaks and 4OHT in the presence of 1 nM E2 in MCF7:WS8 cells. Data are the mean of 3 replicates ± s.d. **b** Cell count after 7 days treatment of separated compound 29 peaks and Laso in the presence of 1 nM E2 in T47D cells. E2-only treatment was at 1 nM but placed in the 10 nM column for comparison purposes. **c** Cell count after 7 day treatment of Y537S *ESR1* MCF7 cells with 1 nM E2 or 1 nM E2 + 1 µM SERM or SERD normalized to vehicle control. **d** In-cell Western of MCF7:WS8 breast cancer cells treated 100 nM compound for 24 h. Data are the mean of 3 replicates ± s.d. **e** Inhibition of ERα-SRC3 binding in the presence of 1 nM E2. **f** Table of IC_50_ values of ERα-SRC3 binding inhibition. Data are the mean of three replicates ± s.d. Error bars represent s.d. Significance determined by unpaired *t* test where *p < 0.05, **p < 0.005, ***p < 0.0005, ns = not significant.

The fluoropropyl-containing T6I-29 showed a slightly improved anti-proliferative efficacy over the propyl-containing T6I-14. With T6I-14, chiral affinity purification showed that one enantiomer disproportionately contributed to these activities. As such, we used chiral affinity chromatography to separate the enantiomeric species of T6I-29. We were able to separate two discrete peaks, which could then be further separated into two additional peaks each. These molecules are now termed 29-1A and 29-1B and 29-2A and 29-2B as they came from the first and second initial peaks, respectively. We measured their effect on the cellular proliferation of MCF7:WS8 breast cancer cells at 1, 100, and 1000 nM in the presence of 1 nM E2 compared to 4OHT (Fig. 8a). Additionally, impact on cellular proliferation was measured in T47D breast cancer cells at 10, 100, and 1000 nM in the presence of 1 nM E2 compared to laso. Surprisingly, each separated peak showed a similar cell count to either 4OHT or laso across all three doses with no clear enantiomeric preference (Fig. 8b). As laso and RAD1901 are under clinical evaluation in Y537S *ESR1* breast cancer patients^6,38^, we measured the anti-proliferative activities of T6I-29-1A alongside 4OHT, ICI, laso, and RAD1901 at 1 µM in homozygous Y537S *ESR1* MCF7 cells (kindly donated by Dr. Sarat Chandarlapaty, Fig. 8c). T6I-29-1A showed a significant reduction in proliferation compared to 4OHT (*p* = 0.001) and showed a similar efficacy to ICI, laso, and RAD1901.

An in-cell Western assay was used to measure the effect of the purified T6I-29 enantiomers on the accumulation of ERα in T47D breast cancer cells at 1 μM dose. While T6I-14-1 enhanced ERα levels past 4OHT, T6I-29-1A and 1B moderately decreased receptor levels to slightly below vehicle (Veh). T6I-29-2A and 2B showed a moderate increase over vehicle (Fig. 8c). NanoBiT was used to measure the impact of T6I-29-1A on ERα-SRC3 binding between 0.05 and 10 μM alongside T6I-14-1, laso, 4OHT, and RAD1901. Here, laso showed highly potent and effective inhibition of SRC3 binding with an IC_50_ of 100 pM. Conversely, both T6I molecules showed similarly weak inhibition of SRC3 binding with micromolar IC_50_ values (Fig. 8d). Together, these data suggest that the T6Is are effectively antagonizing ERα activities in breast cancer cells but are about 10-times less potent than RAD1901 at antagonizing coactivator binding.

### T6I-29-1A shows unexpected effects on the transcriptome of T47D breast cancer cells

**Fig. 9:**
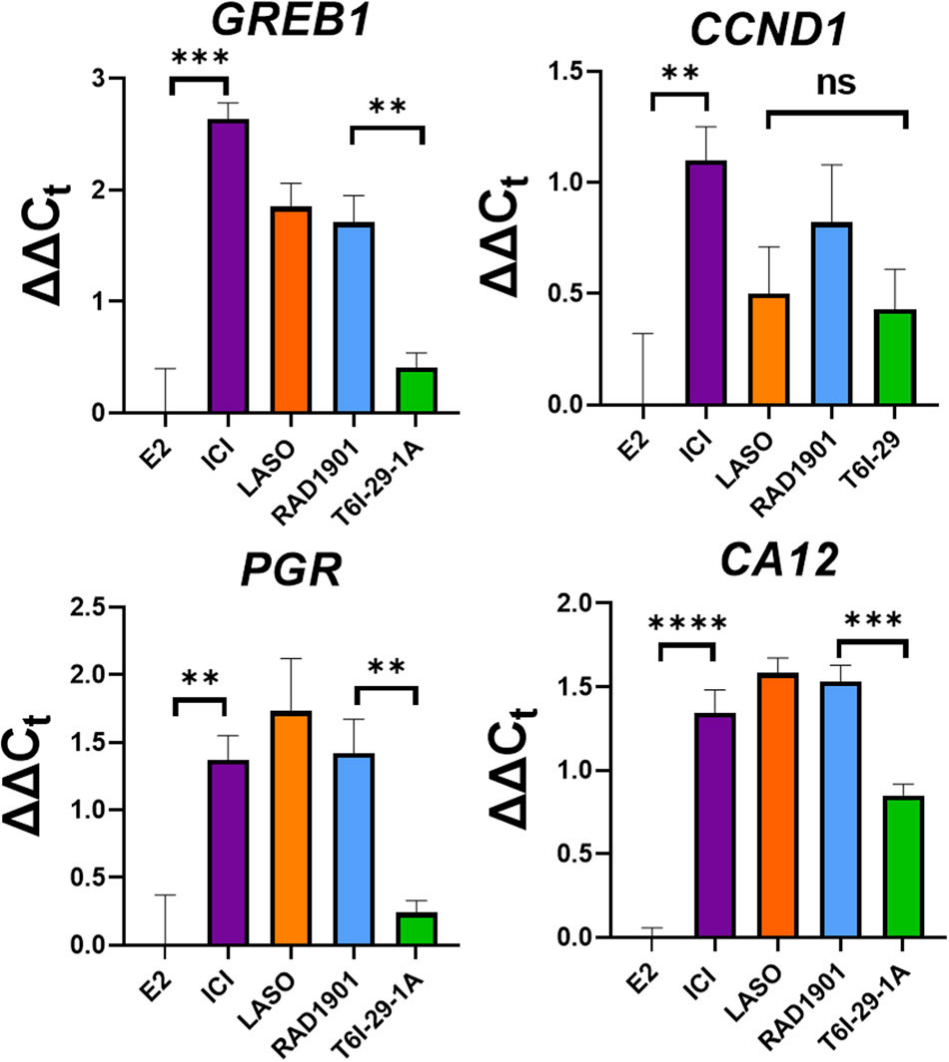
T6I-29-1A shows reduced ERα target gene downregulation compared to ICI, Laso, and RAD1901. Data are the mean of 3 replicates ± s.d. Error bars represent s.d. ΔΔC_t_ = ΔC_t_(drug + E2) – ΔC_t_(E2). Significance determined by unpaired *t* test where **p < 0.005, ***p < 0.0005, ****p < 0.00005, ns = not significant.

T6I-29-1A showed a reduced ability to antagonize transcriptional coactivator binding but similar anti-proliferative potencies to 4OHT and laso in breast cancer cells. This discrepancy suggested that another mechanism besides antagonism of SRC3 binding contributes to its anti-proliferative efficacy. We first tested the ability of T6I-29-1A to reduce the transcription of canonical ERα target genes including *GREB1*, *CCND1*, *PGR*, and *CA12*. T47D breast cancer cells were treated for 16 h with veh (DMSO), 1 nM E2 or 1 nM E2 + 1 μM T6I-29-1A, ICI, Laso, or RAD1901 and gene expression was measured by qPCR and normalized to E2-only treatment. T47D cells were chosen because the T6I scaffold showed marginally improved efficacy while T6I-29 showed largely equal efficacy in both MCF7:WS8 and T47D cells (Fig. 5). E2 showed an induction of expression for each of these genes compared to veh (Supplementary Fig. 6). While T6I-29-1A downregulated the expression of these genes, it did so with reduced efficacy compared to RAD1901, laso, and ICI (Fig. 9). It only matched laso for *CCND1* downregulation.

**Fig. 10:**
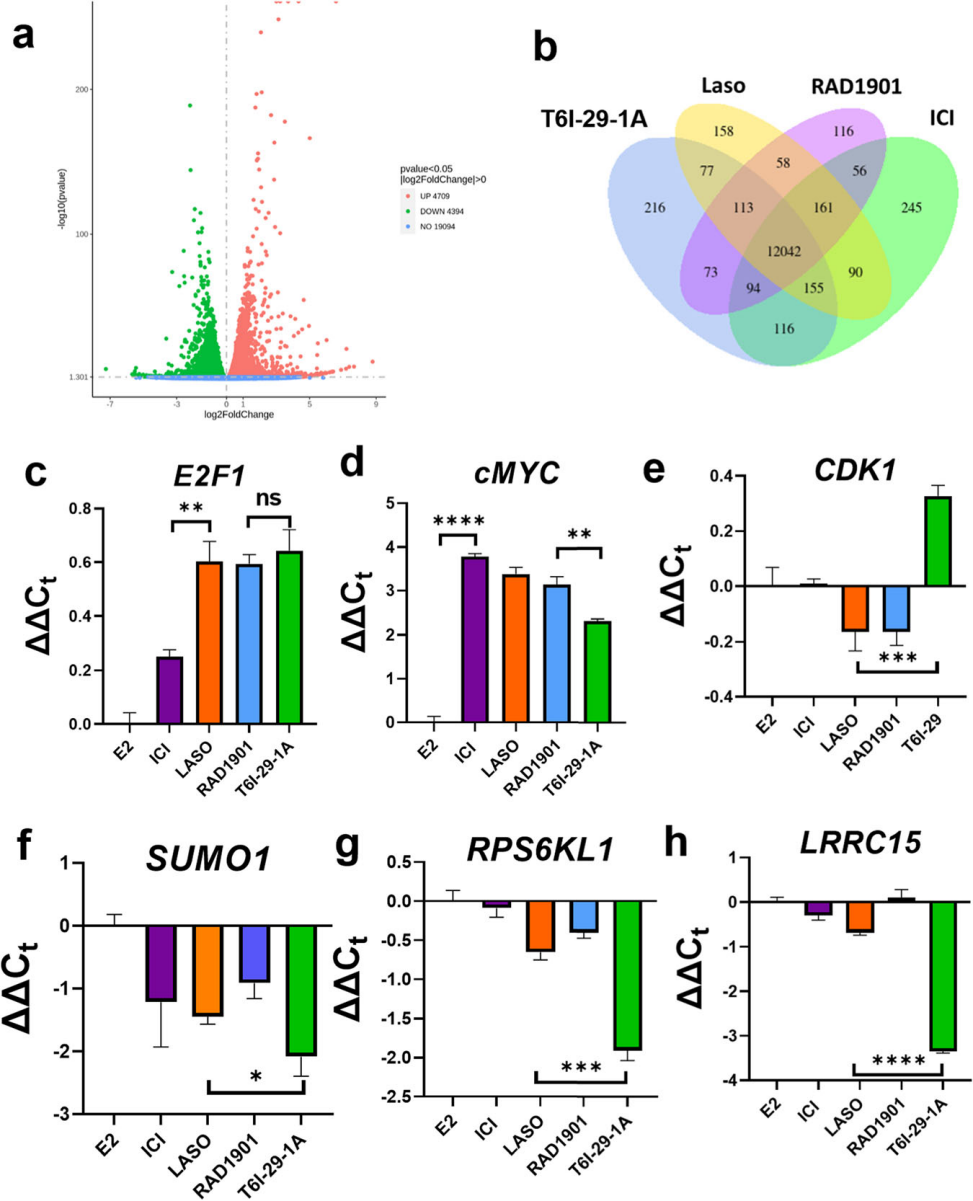
T6I-29-1A affects canonical and cryptic genes in T47D breast cancer cells. **a** Volcano plot differences between 1 nM E2 and veh treated cells highlighting the significant hormone sensitivity of the T47D breast cancer cells. **b** Venn diagram of overlapping genes between treatments. **c**–**h** Relative mRNA levels of newly identified genes *E2F1*, *cMYC*, *CDK1*, *SUMO1*, *RPS6KL1*, and *LRRC15*. ΔΔC_t_ = ΔC_t_(drug + E2) – ΔC_t_(E2). Data are the mean of three replicates ± s.d. and error bars show s.d. Significance determined by unpaired *t* test where *p < 0.05, **p < 0.005, ***p < 0.0005, and ****p < 0.00005.

We next used RNA transcriptomic analysis (RNAseq) to identify differentially affected genes. Serum starved T47D breast cancer cells were treated for 16 h with veh, 1 nM E2, or 1 nM E2 + 1 μM T6I-29-1A, laso, RAD1901, or ICI before the mRNA was isolated and sequenced. T47Ds were chosen because they showed a slightly enhanced sensitivity to the anti-proliferative effects of T6I-29-1A. All sequencing runs were performed with three replicates per treatment. Figure 10 shows the results from the RNAseq experiments in T47D cells. Supplementary Fig. 7 shows a heatmap of every replicate. As expected, 1 nM E2 induced a significant number of differentially regulated genes with 4709 upregulated and 4394 downregulated (Fig. 10a). Overall, T6I-29-1A showed slightly lower effects on the number of differentially regulated transcripts compared to E2-only versus treatments. Supplementary Fig. 8 shows volcano plots of differentially transcribed genes for each antagonist versus E2 alone. Comparison of overlapping transcripts between the antagonists in the presence of E2 versus E2-only treatment suggests that T6I-29-1A largely engages the same transcriptional programs as the other antiestrogens with 12,042 overlaps observed (Fig. 10b). ICI showed the most unique transcripts with 245, followed by T6I-29-1A with 216, laso with 158, and RAD1901 with 116. Compared to the other treatments, T6I-29-1A shared the most overlapping transcripts with ICI at 116, followed by 77 for laso, and 73 for RAD1901.

Disease ontology pathway analysis highlighted breast cancer as the major disease indication across all treatments (Supplementary Fig. 9), further supporting the ERα target engagement of T6I-29-1A. Reactome pathway analysis shows that the transcripts of T6I-29-1A are related to genome and cell cycle checkpoint pathways, similar to the other antiestrogens (Supplementary Fig. 10). Interestingly, pathways relating to SUMO E3 ligases were significantly enriched with T6I-29-1A and ICI but not laso or RAD1901. SUMOylation is an ERα post-translational modification induced by ICI and corresponds to reduced AF-1-dependent transcriptional activation in breast cancer cells^15^.

Differential gene expression analysis of this data set pointed to several cancer-relevant genes that were further studied by qPCR. Two well-characterized ERα target genes, *E2F1* and *cMYC* were identified in the RNAseq (in addition to *PGR*, *CCND1*, *GREB1*, and *CA12*). By qPCR, T6I-29-1A showed identical downregulation of *E2F1* compared to laso and RAD1901, while ICI showed a reduced impact (Fig. 10c). T6I-29-1A also showed a significant but slightly reduced antagonism of *cMYC* (Fig. 10d). Interestingly, *CDK1* and *SUMO1* were uniquely affected by T6I-29-1A. By qPCR, only T6I-29-1A reduced while laso and RAD1901 slightly upregulated *CDK1* (Fig. 10e). Comparison of differentially expressed genes relating to SUMO in the RNAseq data suggest that E2 downregulates *SUMO1* and upregulates desumoylating enzymes including *SENP1* and *SENP8*. While, both ICI and T6I-29-1A upregulated *SUMO1* and downregulated the SENP enzymes. By qPCR T6I-29-1A showed a significant (*p* = 0.02) upregulation of *SUMO1* compared to laso and RAD1901 (Fig. 10f) but was not significantly different compared to ICI by *t* test. We did not observe statistically significant differences to *SENP1* or *SENP8* expression for any treatments (Supplementary Fig. 11A, B). However, comparison of E2 versus veh shows that *SENP1* expression is enhanced while *SUMO1* is significantly downregulated (Supplementary Fig. 11C).

Two cryptic genes *RPS6KL1* and *LRRC15* were significantly and uniquely enriched in the T6I-29-1A RNAseq data compared to the other antagonists. By qPCR, *RPS6KL1* is significantly upregulated by T6I-29-1A and only slightly enriched by laso and RAD1901 (Fig. 10g). *LRRC15* showed a significant upregulation for T6I-29-1A but not for the other treatments (Fig. 10h). A literature review showed little information on *RPS6KL1* but *LRRC15* miRNA has been correlated with invasive breast cancer potential^39^. Interestingly, by qPCR E2 compared to vehicle showed little change in *E2F1*, *CDK1* and *RPS6KL1*. *SUMO1* and *LRRC15* were significantly downregulated, while *cMYC* was significantly upregulated (Supplementary Fig. 11). As such, it remains unclear as to the role of *RPS6KL1* and *LRRC15* played in these cells, except that T6I-29-1A largely opposes the effects of E2 on the expression of these genes. To determine whether similar affects are present in another cell line, we treated MCF7:WS8 breast cancer cells in the same manner as above and performed qPCR (Supplementary Fig. 12). Interestingly, T6I-29-1A largely elicits similar effects between the two cell lines with the exception of *cMYC* where a slight upregulation was observed. Laso also upregulated *cMYC* but it was significantly less (*p* = 0.02) than T6I-29-1A. Interestingly, the greatest differences were observed with ICI treatment, where a greater downregulation of *E2F1* and *CDK1* and upregulation of *SUMO1* was measured. Together, these data show that T6I-29-1A targets therapeutic and cryptic genes within the breast cancer cell, which oppose E2.

### ICI and T6I-29-1A induce SUMO1 expression in T47D breast cancer cells

**Fig. 11:**
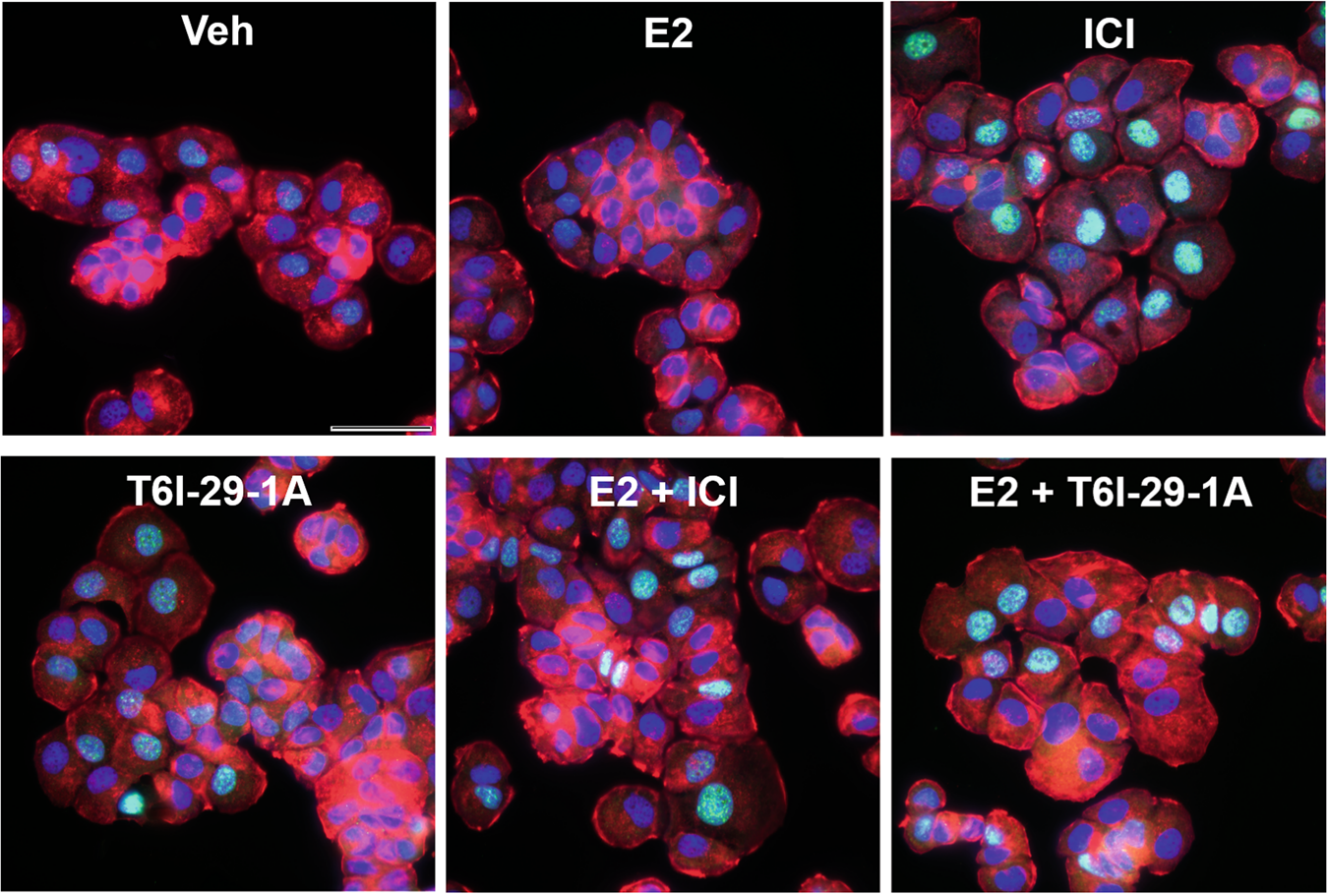
ICI and T6I-29-1A enhance SUMO1 expression in T47D breast cancer cells. SUMO1 immunofluorescence staining of T47D breast cancer cells treated with veh, 1 nM E2, or 1 µM ICI or T6I-29-1A in the presence and absence of E2. SUMO1 is shown in green, actin (phalloidin) in red, and nuclei (DAPI) in blue. Images were taken with a 60x objective. Scale bar = 50 µm.

Each antiestrogen, but especially T6I-29-1A and ICI, showed a significant enrichment of *SUMO1* transcripts in T47D breast cancer cells. While, treatment with E2 downregulated *SUMO1* gene expression compared to veh control. Immunofluorescence was used to study the effects of these treatments on SUMO1 protein expression in these cells. T47D breast cancer cells were grown directly on cover slips, serum starved, then treated with veh, 1 nM E2 or 1 µM ICI or T6I-29-1A in the presence and absence of 1 nM E2 for 16 h before immunostaining. Figure 11 shows representative images for each treatment. Cellular localization was resolved using phalloidin to stain actin and DAPI to stain nuclei. Few cells showed SUMO1 staining in the veh and E2-treatment conditions. ICI and T6I-29-1A showed enriched SUMO1 expression in the nuclei of these cells. However, E2 appeared to mitigate the SUMO1 induction by both ICI and T6I-29-1A. Quantification of the relative fluorescence in these signals shows that ICI and T6I-29-1A induce similar levels of SUMO1, which is reduced in the presence of 1 nM E2 (Supplementary Fig. 13). Together with our RNAseq and qPCR data, these immunofluorescence studies suggest that ICI and T6I-29-1A upregulates while E2 downregulates SUMO1 expression. These data imply that SUMO1 expression likely contributes to the anti-cancer activities of SERMs and SERDs. Further studies are needed to decipher the role and estrogen dependence of SUMO1 in ER + breast cancer pathology.

### T6Is show unconventional SERM-agonist relationships in Ishikawa cells

**Fig. 12:**
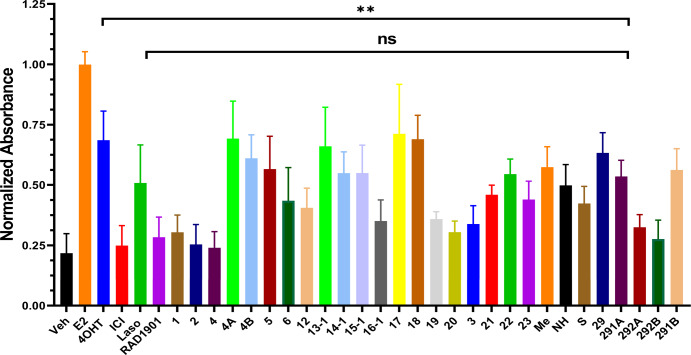
Uterotrophic activities of T6Is in Ishikawa endometrial cells by alkaline phosphatase activity. Data are the mean of three independent replicates ± s.d. and error bars represent s.d. Significance determined by unpaired *t* test where **p < 0.005, n.s. not significant.

SERM enhancement of ERα accumulation in breast cancer cells correlates with uterotrophic activities^22,30^. Despite the inclusion of side arms from known SERDs, most T6Is appeared SERM-like in breast cancer cells as they increased ERα levels. To measure the SERM-agonist activities of the T6Is, we employed an AP activity assay in Ishikawa endometrial cells^22,32,40^. Cells were placed in serum-starved medium and treated for 72 h with vehicle (DMSO), 1 nM E2, or 1 μM 4OHT, ICI, laso, RAD1901, or T6I. Figure 12 shows the results from this experiment and Supplementary Table 1 shows the mean and standard deviation for these experiments (*n* = 3–9). Many T6Is show unexpected AP activities in these cells. For example, T6I-4 showed a similar or greater SERM-like increase to ERα levels compared to 4OHT by in-cell Western at 1 μM dose. Therefore, we would expect these SERMs to show a greater induction of AP (Supplementary Fig. 5). However, T6I-4 showed AP induction similar to a SERD, while 4A and 4B, which were fluoro and chloro azetidines, showed significant induction of AP compared to the propylazetidine-containing T6I-4. Additionally, the racemic mixture compound 29 showed a greater induction of AP1 compared to its separated chiral species. T6I-29-1A showed comparable to activity to laso at 0.54 ± 0.07 and 0.51 ± 0.16 absorbance units, respectively, which was not statistically significant. This difference was significant (*p* = 0.004) compared to 4OHT, which measured 0.68 ± 0.12 absorbance units. In most studies of SERM-agonist activities, AP assays are combined with increases in uterine wet-weight in vivo^22,41,42^. To observe whether degree of AP induction correlated with differences in endometrial proliferation, we measured the effects of E2, 4OHT, ICI, laso, RAD1901, and T6I-29-1A on the cell count of Ishikawa cells after treatment for 144 h (Supplementary Fig. 14). E2 was given at 1 nM while a 1 μM dose of antiestrogen was used. A robust enhancement of proliferation was observed in the E2-treated cells compared to vehicle (DMSO) control. Interestingly, a marginal but insignificant increase to cell count was measured for 4OHT versus vehicle control. Each of the other molecules, including T6I-29-1A showed a significantly (*p* = 0.0043) reduced cell count at the end of the study compared to 4OHT.

## Discussion

In this study, we show how an unconventional chemical scaffold can affect unique ERα structural motifs and engage unexpected transcriptomic effects in breast cancer and endometrial cells. Using x-ray crystal structures of RAD1901and laso as templates, we developed a new tetrahydro-6-isoquinoline (T6I) antiestrogen scaffold. These molecules overwhelmingly favored a SERM-like accumulation of ERα in breast cancer cells, despite the inclusion of side arms from next generation SERDs such as GDC-0927^43^. The T6I scaffold can achieve potent anti-transcriptional and anti-proliferative activities in luminal breast cancer cells. However, they are inferior antagonists of ERα-SRC3 (transcriptional coactivator) binding compared to other SERMs and SERDs. Crystallographic analysis shows that anti-proliferative efficacy correlates with T6I side-arm vector and occupancy near H12 to engage the antagonist conformation similar to other SERMs and SERDs. The T6I core uniquely perturbs H8 by forcing a phenylalanine (F425) away from the hormone-binding pocket to accommodate the ligand. As other unconventional SERMs and SERDs have shown changes to the repertoire of ERα-associated coregulators, it is possible that the T6Is uniquely affect coactivators besides SRC3 or show improved recruitment of an ERα-corepressor^29,44^.

A key finding of this study is T6I-29-1A’s unexpected effect on the T47D transcriptome. With the exception of *E2F1*, T6I-29 showed inferior inhibition of classic ERα target genes compared to laso, RAD1901, and ICI. However, it showed a unique, albeit minor, downregulation of *CDK1*. In MCF7:WS8 breast cancer cells T6I-29-1 maintained a similar degree of *CDK1* downregulation while ICI and RAD1901 now downregulated its expression. Direct inhibition of *CDK1* elicits potent anti-cancer activities in breast cancer cells^45^. Our data suggest that *CDK1* expression may depend on ERα and differences in SERM or SERD-induced expression may be a result of differential ligand effects on receptor structure and/or coregulator expression between cell lines. Indirect inhibition of *CDK1* through ERα may represent a new way to achieve targeted inhibition on a single chemical scaffold. Interestingly, T6I-29 along with ICI showed enhanced SUMO1 expression. It may related to the SUMOylation of ERα itself as ICI induces rapid ERα-SUMOylation to influence transcriptional activity in MCF7 breast cancer cells^15^. However, our observation that E2 downregulated SUMO1 while upregulating deSUMOylating enzymes points to a potentially suppressive role for bulk SUMO1 expression that should be studied further.

Historically the ERα-accumulating effects of SERMs correlate with uterotrophic activities^46,47^. Our findings suggest that induction of ERα degradation may not be required for endometrial ERα antagonism. In breast cancer cells, T6I-4 and T6I-29-1A enhanced ERα accumulation to a similar or greater extent than SERM 4OHT. We therefore expected that these T6Is would induce greater stimulation of AP1 in Ishikawa endometrial cells, as has been observed for tamoxifen and endoxifen^22,48^. However, T6I-4 showed no measurable AP1 activity, while T6I-29-1A showed a weak laso-like stimulation of AP1. As the side arms from T6I-4 and T6I-29 are similar to the SERDs GDC-0927 and SAR439859, it may also be possible that the chemical composition of these molecules prevents uterotrophism rather than ERα degrading activities^4,43^. Even though 4OHT showed a robust stimulation of AP activity, it did not significantly increase Ishikawa endometrial cellular proliferation. Studies of SERM-agonistic activities often measure induction of AP activity in Ishikawa cells before in vivo uterine wet-weight studies in mice and/or rats^22,41,42^. Therefore, an important future direction will be to measure T6I-29-1A uterotrophism by mouse or rat uterine wet-weight. Further studies are also needed to understand the relationship between tissue-specific transcriptional coregulator expression, antiestrogen chemical composition, and SERM-agonist activities.

By engaging unique transcriptional programs, the SERM-like T6Is were effective anti-proliferative agents and exhibited little endometrial stimulation. These structurally unconventional ligands show that probing distinct ERα structural features can reveal novel therapeutic activities. Comprehensive pharmaceutical profiling is needed to assess in vivo activities and tissue-wide effects of these compounds. Nevertheless, this T6I scaffold represents a new opportunity for the development of hormone therapies with unique and favorable tissue-specific activities to treat ER+ breast cancers.

## Methods

### Chemicals, reagents and kits

4-hydroxytamoxifen, 17β-estradiol, and fulvestrant (68392-35-8, 50-28-2, 129453-61-8, respectively) were purchased from Millipore Sigma. Lasofoxifene (HY-A0037) and elacestrant (HY-19822) were purchased from MedChem Express. All cell culture, bacterial expression media and reagents, and quantitative PCR reagents were purchased from Thermo Fisher Inc. A Qiagen RNeasy Mini kit (74106) was used for RNA extraction. Otherwise, individual reagents pertinent to each method can be found below.

### Cell culture

HEK293T, MCF7, and T47D cell lines were purchased from ATCC (CRL-3216, HTB-22, and HTB-133, respectively). Ishikawa cells were purchased from Millipore Sigma (99040201). MCF7:WS8 cells were kindly donated by Dr. Clodia Osipo. MCF7 Y537S *ESR1* breast cancer cells were kindly donated by Dr. Sarat Chandarlapaty. All cell lines were tested for mycoplasma quarterly and their identities confirmed using STR profiling through ATCC before the start of experiments. WT and Y537S *ESR1* MCF7 and MCF7:WS8 cells were cultured in DMEM supplemented with 10% FBS and 6.5 μg/mL bovine insulin. T47D cells were cultured with RPMI supplemented with 10% FBS. Ishikawa cells were cultured with MEM supplemented with 2 mM l-glutamine, 1% non-essential amino acids, and 5% FBS.

### ERα ligand binding domain expression and purification

All x-ray crystal structures used an ERα ligand binding domain (LBD) (residues 300–550) construct with the following mutations to facilitate crystallization: C381S, C417S, C530S, and L536S. A pET21a+ plasmid containing a hexa His-TEV-tagged ERα LBD was codon optimized for *E.coli* expression, synthesized, and subcloned by Genscript Inc. The plasmid was transformed into BL21(DE3) *E.coli*. Cells were grown in Miller-LB broth with shaking at 37 °C until they reached an OD_600_ of 0.3 after which the temperature was reduced to 16 °C. Protein expression was induced with 0.3 mM IPTG for 16 h. Cells were harvested by centrifugation at 4000 × *g* at 4 °C for 15 min then resuspended in 20 mL/g cell paste with 50 mM HEPES pH 8.0, 500 mM NaCl, 20 mM imidazole pH 8.0, 5% glycerol, 0.1 mM TCEP, and appropriate EDTA-free protease inhibitor cocktail. Cells were lysed by sonication then supernatant separated by centrifugation for 45 min at 17,000 × *g* at 4 °C. His-TEV ERα LBD was purified first on a 5 mL HisTrap Fast Flow IMAC column (GE), then His-TEV protease was used to His tag from the protein, and a final purification was performed on a Superdex 16/600 size-exclusion column pre-equilibrated with 50 mL HEPES pH 8.0, 250 mM NaCl, 5% glycerol, 0.1 mM TCEP. A single peak eluted off the column corresponding to an ERα LBD dimer. A single band was visualized on an SDS-PAGE gel. The protein was concentrated to 10 mg/mL and flash frozen for later use.

### Protein crystallization, x-ray data collection, and structure solution

ERα LBD at 10 mg/mL was incubated with 1 mM ligand overnight at 4 °C. Mixture was centrifuged at 21,100 × *g* for 30 min at 4 °C to separate any insoluble molecule or precipitated protein. Hanging drop vapor diffusion was used to generate ERα LBD-drug co-crystals at 2.5, 5, and 10 mg/mL. Clear crystals emerged between 1 day and 2 months at room temperature in PEG 3350 or 8000 (10–30%) with 100 mM MgCl_2_ and HEPES pH 6–8. Crystals for RAD1901 were hexagonal pucks. T6I crystals were dodecahedrons. Mother liquor or mother liquor plus 30% glycerol were used as the cryoprotectant. All x-ray data sets were collected at the Advanced Photon Source Argonne National Laboratories, Argonne Illinois on the SBC 19-BM beamline (0.97 Å). Supplementary Table 2 is a table of crystallization data collection and refinement statistics. Data were indexed, scaled, and merged using HKL-3000. CCP4i was used for all molecular replacements using Phaser with PDB: 5UFX as the search model with the ligand removed^22^. Models were refined using iterative rounds of Phenix Refine and manual inspection with Coot^49^. Clear densities for each ligand were observed after one round of refinement. Elbow was used to generate ligand constraints. Unresolved residues were not included in the final model. No Ramachandaran outliers are present in the final models. Supplementary Fig. 15 shows stereoview 2mFo-DFc maps for each ligand in the binding pocket. All x-ray structure images were made with Pymol.

### Transcriptional reporter gene assay

This assay was performed in accordance with a previous report^30^. MCF7 breast cancer cells with a 3x-estrogen response element-green fluorescent protein (GFP) reporter gene construct under a CMV promoter were plated in 96-well dishes at 3000 cells per well. Cells were cultured for 48 h in stripped media that included charcoal stripped fetal bovine serum (FBS). Cells were treated with increasing concentrations of antiestrogen (5 pm to 1 µM) for 24 h. Subsequently, percent GFP-positive cells were imaged using a BioTek Cytation 5 live cell imager. All assays were performed three times with three technical replicates each.

### Live-cell halo-ERα accumulation assay

This assay was performed in accordance with a previous report^30^. T47D breast cancer cells with a halo-ERα under a doxycycline (dox) control were grown in 96-well dishes starting at 3000 cells per well. Cells were placed in serum-starved media for 48–72 h then simultaneously treated with 1 µg/mL dox, and 1 µM Halo-TMR direct (Promega, G2991) alongside 5 pm to 1 µM compound for 24 h. After which cells were imaged using a BioTek Cytation 5 for RFP and normalized to cell count per well. Assays were performed twice with at least three replicates.

### In cell western analysis

In-cell Western analysis was performed in a similar manner as previously described^34^. Briefly, T47D or MCF7:WS8 breast cancer cells were grown in blackout 96-well plates starting at 3000 cells per well. After 24 h, cells were placed in serum-starved media and allowed to acclimate for 48–72 h. Cells were treated with hormone, SERM, or SERD alongside vehicle control for 24 h. Only interior wells were used to avoid edge effects. Cells were fixed with 4% formaldehyde and permeabilized with 0.1% Triton X-100. Plates were blocked with Intercept blocking buffer (LI-COR). Santa Cruz F10 anti-ERα antibody (sc-8002) was used as the primary at 1:200-500 dilution. Secondary antibody was a goat anti-mouse IgG 800 IRDye antibody (926-32210) from LI-COR. Cells were also treated with CellTag700 (926-41090) to control for differences in cell count between wells. All data were collected on a LI-COR Odyssey DLx and analyzed using Emperia Studio. Each experiment was comprised of three replicates.

### NanoBiT ERα-SRC3 assay

HEK293T cells were grown in 96-well clear bottom, white-walled plates to around 70% confluence. PCDNA 3.1 plasmids containing N-terminally tagged smBiT ERα or lgBiT SRC3 nuclear recognition domain (NRD) were kindly donated by Dr. Donald P. McDonnell. These plasmids were co-transfected with a total of 0.1 µg smBiT ERα and 0.1 µg LgBiT SRC3 NRD using Turbofectin 8.0 at a 3:1 ratio. After 24 h cells were placed in serum-starved media and allowed to acclimate for an additional 48–72 h. Cells were treated with each compound in the presence of 1 nM E2 alongside 1 nM E2-only and DMSO controls for 4 h then treated with NanoGlo substrate and imaged for luminescence after 5 min using a BioTek Cytation 5. Data shown are the average of three independent replicates.

### Cellular proliferation

MCF7, MCF7:WS:8, Y537S *ESR1* MCF7 and T47D breast cancer cell lines were grown in 96-well dishes at a starting cell count of 1000 cells per well. Cells were placed in serum-starved medium for 48–72 h before they were treated with vehicle, 1 nM E2, or SERM or SERD + 1 nM E2. Cells were grown in a BioSpa attached to a BioTek Cytation 5 and were automatically counted every twelve hours for 7–10 days or until the E2-only wells reached confluence. Media was replaced every 4 days and drug was maintained in each media change. Each study was repeated three times with three technical replicates each.

### Crystal violet cellular viability assay

MCF7 breast cancer cells were placed in 24-well dishes at 15,000 cells per well. After 24 h, they were placed in serum-starved media for 48 to 72 h. Cells were treated with vehicle (DMSO), 1 nM E2, or 1 nM E2 + 1–1000 nM 4OHT, ICI, or T6I-14-1 for 7 days. After treatment, cells were stained with 0.4% crystal violet in 50% methanol for 30 min. Cells were washed with PBS to remove excess dye. 500 µL of methanol was then added to each well to dissolve the dye and placed on a rocker for 30 min at room temperature and the absorbance at 570 was recorded.

### RNA sequencing

T47D breast cancer cells were grown in 6-well dishes at 50,000 cells per well. After 24 h they were placed in serum starved media for 48 h then treated with vehicle (DMSO), 1 nM E2, or 1 nM E2 + 1 µM ICI, laso, RAD1901, or T6I-29-1A for 16 h in triplicate. After 24 h, they were placed in serum-starved media for 48 h. They were then treated with vehicle (DMSO), 1 nM E2, or 1 µM 4OHT, laso, RAD1901, ICI, or T6I-29-1A. RNA was isolated using a Qiagen RNeasy Kit then sent to Novogene for sequencing and bioinformatics analysis.

### Quantitative PCR (qPCR)

Cells were treated as indicated. Total RNA was isolated with the RNeasy Kit (Qiagen). cDNA was made using M-MLV Reverse transcriptase (Invitrogen) and carried out per the manufacturer’s instructions.

Primers:

*GREB1* F: 5′-CTGCCCCAGAATGGTTTTTA-3′

*GREB1* R: 5′-GGACTGCAGAGTCCAGAAGC-3′

*CCND1* F: 5′-AACTACCTGGACCGCTTCCT-3′

*CCND1* R: 5′-CCACTTGAGCTTGTTCACCA-3′

*PGR* F: 5′-AGCCAGAGCCCACAATACAG-3′

*PGR* R: 5′-GACCTTACAGCTCCCACAGG-3′

*CA12* F: 5′-GACCTTTATCCTGACGCCAGCA-3′

*CA12* R: 5′-CATAGGACGGATTGAAGGAGCC-3′

*E2F1* F: 5′-GGACCTGGAAACTGACCATCAG-3′

*E2F1* R: 5′-CAGTGAGGTCTCATAGCGTGAC-3′

*cMyc* F: 5′-TTCGGGTAGTGGAAAACCAG-3′

*cMyc* R: 5′-CAGCAGCTCGAATTTCTTCC-3′

*CDK1* F: 5′-GGAAACCAGGAAGCCTAGCATC-3′

*CDK1* R: 5′-GGATGATTCAGTGCCATTTTGCC-3′

*SUMO1* F: 5′-AGCAGTGAGATTCACTTCAAAGTG-3′

*SUMO1* R: 5′-TCTGACCCTCAAAGAGAAACCTG-3′

*RPS6KL1* F: 5′-CATCTTCCTGCACCTGGAGCAT-3′

*RPS6KL1* R: 5′-AGCTGAGCCTTCATCCTCTCCT-3′

*LRRC15* F: 5′-CGTTGCTGTTCCAAGCGTCCAT-3′

*LRRC15* R: 5′-GCTCAGTGGTAGAAGAGACGGA-3′

### Immunofluorescence (IF) assay

T47D cells were cultured on coverslips for 24 h followed by 48 h growth in serum starved media. Cells were subsequently treated with vehicle (DMSO), 1 nM E2, 1 µM ICI, 1 nM E2 + 1 µM ICI, 1 µM T6I-29-1A, or 1 nM E2 + 1 µM T6I-29-1A for 24 h. Cells were fixed for 10 min with 4% paraformaldehyde at room temperature was followed by three washes with ice-cold PBS-T (0.1% Tween 20, PBS) and 10 min of permeabilization with 0.5% saponin in PBS. Blocking was performed with 1% bovine serum albumin (BSA) in PBS-T for 30 min. Cells were incubated in a humidified chamber with 1:500 rabbit monoclonal anti-SUMO1 antibody (Abcam Ltd, Cambridge, UK, ab32058) in 1% BSA for 1 h at room temperature. Cells were washed 3 times with PBS-T, then incubated in the dark for 1 h with 1:1000 goat anti-rabbit IgG H&L (Alexa Fluor® 488) antibody (Abcam Ltd, ab150077) as well as 1:1000 phalloidin-iFluor 594 reagent (Abcam Ltd, ab176757) in 1% BSA. Finally, after three PBS washes, cells were mounted to slides using Invitrogen^TM^ ProLong^TM^ glass antifade mountant with NucBlue counterstain (Fisher, P36983). Image acquisition was performed using an Olympus BX53 microscope and Olympus cellSens Dimension 1.16 software at 60x magnification. Quantification of nuclear fluorescent signal was performed using ImageJ 1.53t software. Fluorescent intensity of all nuclei within captured frames were measured and normalized by subtracting mean background fluorescence of 5 representative areas.

### Alkaline phosphatase (AP) assay

AP assays were performed as described^22^. Briefly, 15,000 Ishikawa cells were seeded into 96-well plates then placed in serum-starved medium for 48–72 h. Cells were treated for three days with veh, hormone, SERM, or SERD. The media was aspirated and the cells were frozen at −80 °C for at least 24 h. Cells were thawed then incubated with p-nitrophenyl phosphate (ThermoFisher) chromogenic AP substrate. After 60 min at 40 °C, absorbance was read at 405 nm on a BioTek Cytation 5 plate reader.

### Statistical analysis

X-ray crystallographic statistics were obtained from HKL 3000 and Phenix. Graphpad Prism 9.0 was used to analyze the cellular proliferation, ERα accumulation (in-cell Western and halo-tag), reporter gene and qPCR assays. All biological assays were performed at least three times. Where reported, statistical significance was determined using *t* test and *p*-values ≤ 0.05 were considered statistically significant and were stated where relevant in each figure. For IC_50_ measurements *R*^2^ was used for the quality of the fit. Fits were considered valid if *R*^2^ > 0.90.

### Chemical synthesis

Reagents and solvents were used as received from commercial suppliers. ^1^H NMR spectra were obtained on a Bruker AVANCE 400 spectrometer at 400 MHz with tetramethylsilane was used as an internal standard for proton spectra. Thin-layer chromatography was performed using Merck TLC silica gel 60 F_254_ plates. Visualization of TLC plates was performed using UV light (220, 230 nm). The mass spectra were obtained on a Waters Acquity LC-MS spectrometer using Electrospray Ionization. HPLC analysis were performed with using the method shown below and gradient found in Supplementary Table 3.

### HPLC method

Column: Eclipse plus C18, (100 × 4.6 mm, 3.5 μm).

Purge Flow: 0.8 mL/min

Mobile Phase **A**: 0.05% TFA in Water.

Mobile Phase **B**: 0.05% TFA in Aetonitrile.

### Prep HPLC purification method (Method-A)

High-pressure liquid chromatography (HPLC) purification analysis was performed using a WATERS mass based auto purification system with a binary solvent system A and B using a gradient elution found in Supplementary Tables 4–6:

### HPLC Method

Column: GEMINI NX C18(150 × 30 mm, 10 µm).

Mobile Phase **A**: 10 mM Ammonium Formate in water.

Mobile Phase **B**: Acetonitrile

Flowrate: 30 mL/min,

Injection volume: 500 µL,

Runtime: 20 min,

Detection: 220 and 254 nm

### Synthesis of intermediate H-8

#### Introduction

Synthesis of intermediate **H-8** was achieved from the commercially available **H-1**. **H-1** was reacted with titanium isopropaxide, BF_3_•EtO and EtMgBr to afford **H-2**. **H-2** was reacted with **H-3**, TFA, toluene in Microwave to achieve **H-4**. **H-4** was reacted with **H-5**, TEA to afford **H-6**. **H-6** was treated with 1.0 M BBr_3_ to afford demethylated compound **H-7**, which was protected with benzyl treating with benzyl bromide to afford **H-8**. Supplementary Fig. 16 shows the synthesis scheme.

#### Preparation of H-2

**H-1** (10.00 g, 68.02 mmol) in dry THF (300 mL) was charged with titanium tetra isopropoxide (24.00 mL, 81.62 mmol) followed by the addition of EtMgBr (136.0 mL, 136.05 mmol, 1.0 M in THF) at room temperature under argon atmosphere (Note: The reaction was exothermic during Grignard addition). The resulting mixture was stirred for 1 h at room temperature. BF_3_•Et_2_O (20.00 mL, 136.05 mmol) was added to the reaction mixture and stirred for 1 h. The reaction mixture was poured in cold aqueous solution of (10%) NaOH (100.0 mL) and diluted with EtOAc (1000 mL). The resulting reaction mixture was filtered and the organic layer was washed with water (2 × 100 mL) and brine (2 × 100 mL). The resulting reaction mixture was dried over Na_2_SO_4_, filtered, and concentrated under reduced pressure to obtain **H-6** [11.50 g (crude), AMRI lot # IN-GUM-C-178] as light yellow oil; ESI-MS: *m/z* = (M + H)^+^ 178.24.

#### Preparation of H-4

**H-3** [3.00 g, 12.93 mmol, AMRI lot # IN-GUM-C-146] in toluene (20.0 mL) was charged with **H-2** [4.57 g, 25.86 mmol, AMRI lot # IN-GUM-C-178] TFA (15.0 mL) at room temperature. The resulting reaction mixture was stirred at 140 °C for 45 min in microwave. The reaction mixture was cooled to room temperature, diluted with EtOAc(500 mL), and washed with sat. aq. NaHCO_3_ (3 × 150 mL) and brine (3 × 150 mL), dried over Na_2_SO_4_, filtered and concentrated under reduced pressure to obtain crude material. The obtained crude material was purified by flash chromatography by using silica gel (100–200 mesh) and eluted with (0–5%) MeOH in CH_2_Cl_2_. Combined column fractions were concentrated under reduced pressure to afford **H-4** [3.80 g, 75% (yield is combined four batches), AMRI lot # IN-GUM-D-10) as brown oil.

^1^H NMR (400 MHz, CDCl_3_): δ 7.65 (d, *J* = 8.4 Hz, 2H), 7.09 (d, *J* = 8.4 Hz, 2H), 6.64–6.59 (m, 2H), 6.49 (d, *J* = 8.8 Hz, 1H) 4.87 (s, 1H), 3.68 (s, 3H), 3.25 (bd, *J* = 16.4 Hz,1H), 2.66 (bs, 1H), 2.28 (bd, *J* = 16.4 Hz,1H), 0.57–0.48 (m, 3H), 0.33–0.30 (m, 1H); ESI-MS: *m/z* = (M + H)^+^ 392.02.

#### Preparation of H-6

**H-4** [3.80 g, 9.71 mmol, AMRI lot # IN-GUM-D-10] in CH_2_Cl_2_ (50 mL) was charged with TEA (2.70 mL, 19.43 mmol) followed by **H-5** (1.30 mL, 11.66 mmol) at 0 °C, under argon atmosphere. The reaction mixture was stirred for 4 h and diluted with CH_2_Cl_2_ (150 mL). The organic layer was washed with water (2 × 50 mL) and brine (2 × 50 mL). The resulting mixture was dried over Na_2_SO_4_, filtered, and concentrated under reduced pressure to obtain crude material. The obtained crude material was purified by flash chromatography by using silica gel (100–200 mesh) and eluted with (25–30%) EtOAc in hexanes. Combined column fractions were concentrated under reduced pressure to afford **H-6** [3.80 g, 79.00%, AMRI lot # IN-GUM-D-12] as an off white foam solid.

^1^H NMR (400 MHz, CDCl_3_): δ 7.58 (d, *J* = 8.4 Hz, 2H), 7.48–7.38 (m, 5H), 7.16 (d, *J* = 8.0 Hz, 2H), 7.01 (d, *J* = 8.4 Hz, 2H), 6.82 (dd, *J* = 8.4 Hz, 2.0 Hz, 1H), 6.71(s, 1H), 3.84 (s, 3H), 3.73 (bd, *J* = 16.8 Hz,1H), 2.17 (bd, *J* = 16.8 Hz,1H), 0.30–0.28 (m, 3H), −0.033 to −0.028 (m, 1H); ESI-MS: *m/z* = (M + H)^+^ 496.03.

#### Preparation of H-7

**H-6** [3.80 g, 7.67 mmol, AMRI lot # IN-GUM-D-12] in CH_2_Cl_2_ (50 mL) was charged with BBr_3_ (20.0 mL, 19.19 mmol, 1.0 M in CH_2_Cl_2_), at 0 °C, under argon atmosphere. The reaction mixture was stirred for 2 h and quenched with MeOH (10 mL) at 0 °C. The resultant reaction mixture was stir at room temperature for 1 h, after 1 h reaction mixture was directly concentrated under reduced pressure to obtained crude material. The obtained crude was purified by flash chromatography by using silica gel (100–200 mesh) and eluted with 0–2% MeOH in CH_2_Cl_2_. Combined column fractions were concentrated under reduced pressure to afford **H-7** [3.72 g, 98.00%, AMRI lot # IN-GUM-D-15] as an off-white foam solid

^1^H NMR (400 MHz, CDCl_3_): δ 9.45 (s, 1H), δ 7.67 (d, *J* = 8.0 Hz, 2H), 7.45 (bs, 5H), 7.08 (d, *J* = 8.0 Hz, 2H), 6.91 (d, *J* = 8.4 Hz, 1H), 6.79 (bs, 1H), 6.67 (dd, *J* = 8.4 Hz, 2.4 Hz, 1H), 6.64 (s, 1H), 3.62 (bd, *J* = 16.8 Hz,1H), 2.20 (bd, *J* = 16.8 Hz,1H), 0.29 (bs, 2H), 0.13 (bs, 1H), −0.028 to −0.033 (m, 1H); ESI-MS: *m/z* = (M + H)^+^ 482.01.

#### Preparation of H-8

**H-7** [3.70 g, 7.90 mmol, AMRI lot # IN-GUM-D-15] in DMF (50 mL) was charged with K_2_CO_3_ (1.60 g, 11.85 mmol), followed by benzyl bromide (1.18 mL, 9.87 mmol) at room temperature under argon atmosphere. The reaction mixture was stirred for 24 h. The reaction mixture was poured in an ice-cold water (200 mL), product was extracted with EtOAc (3 × 50 mL), and combined organic layer was washed with cold brine (2 × 50 mL), water (2 × 50 mL), dried over Na_2_SO_4_, filtered and concentrated under reduced pressure to obtained crude material. The obtained crude material was purified by flash chromatography by using silica gel (100–200 mesh) and eluted with (15–20%) EtOAc in hexanes. Combined column fractions were concentrated under reduced pressure to afford **H-8** [3.20 g, 78.0 %, AMRI lot # IN-GUM-D-17] as an off-white solid.

^1^H NMR (400 MHz, CDCl_3_): δ 7.58 (d, *J* = 8.4 Hz, 2H), 7.47–7.35 (m, 11H), 7.17 (d, *J* = 8.0 Hz, 2H), 7.01 (d, *J* = 8.4 Hz, 1H), 6.89 (dd, *J* = 8.4 Hz, 2.4 Hz, 1H), 6.80 (d, *J* = 2.4 Hz, 1H), 5.09 (s, 2H), 3.72 (bd, *J* = 16.8 Hz,1H), 2.16 (bd, *J* = 16.8 Hz,1H), 0.39–0.20 (m, 3H), −0.031 to −0.058 (m, 1H); ESI-MS: *m/z* = (M + H)^+^ 572.05.

### Synthesis of compound-1

#### Introduction

Synthesis of **compound-1** was achieved from synthesized intermediate **H-8**. **H-8** was reacted with **H-9** in sealed tube to achieved **H-10**. **H-10** was treated with TiCl_4_ to afford **compound-1**. Supplementary Fig. 17 shows the synthetic scheme.

#### Preparation of H-10

**H-8** (0.25 g, 0.437 mmol, AMRI lot # IN-GUM-D-17), CuI (9.0 mg, 0.043 mmol), Cs_2_CO_3_ (0.570 g, 1.751 mmol), 1,10-phenathroline (18.0 mg, 0.087 mmol) and **H-9** (0.24 g, 1.751 mmol) in butyronitrile (0.2 mL) was stirred at 130 °C for 24 h in sealed tube. The progress of the reaction was monitored by TLC and UPLC-MS. Reaction mixture was cooled to room temperature and diluted with EtOAc (150 mL), copper salts were filtered through celite pad, washed with excess of ethylacetate (25 mL), filtrate was concentrated under reduced pressure to afford **H-10** [0.21 g (crude), AMRI lot # IN-GUM-D-23] as an off white foam solid; ESI-MS: *m/z* = (M + H)^+^ 545.22.

#### Preparation of compound-1

**H-10** (0.210 g, 0.604 mmol, AMRI lot # IN-GUM-D-23) in CH_2_Cl_2_ (10.0 mL) was charged with TiCl_4_ (5.0 mL, 9.064 mmol) at 0 °C. The resulting reaction mixture was stirred at the same temperature for 3 h. The progress of the reaction was monitored by UPLC. The reaction was quenched by pouring in ice cold sat. aq. NaHCO_3_ (100 mL), the resulted material was extracted with 10% MeOH in CH_2_Cl_2_ (3 × 100 mL). The combined organic layer was washed with sat. aq. NaHCO_3_ (3 × 50.0 mL), brine (5 × 50.0 mL), dried over Na_2_SO_4_, filtered, and concentrated under reduced pressure to obtain crude material as a pale yellow solid. The obtained crude material was purified by preparative-HPLC (Method-A). The pure fractions were collected and CH_3_CN was concentrated under reduced pressure. Aqueous layer (10.0 mL) was extracted with 10% MeOH in CH_2_Cl_2_ (3 × 20 mL), dried over Na_2_SO_4_, filtered, and concentrated to afford **compound-1** [0.020 g, 10.10% (over two steps) AMRI lot # IN-GUM-D-25) as an off-white solid.

^1^H NMR (400 MHz, DMSO): δ 9.38 (s, 1H), 7.44 (s, 5H), 7.15 (d, *J* = 8.4 Hz, 2H), 6.88 (d, *J* = 8.4 Hz, 1H), 6.74 (d, *J* = 8.8 Hz, 3H), 6.67–6.62 (m, 2H), 4.75–4.72 (m, 1H), 3.75–3.73 (m, 2H), 3.63–3.59 (m, 1H), 2.96 (bs, 2H), 2.51 (s, 2H), 2.21–2.16 (m, 1H), 0.91–0.87 (m, 3H), 0.27–0.25 (bs, 2H), 0.14–0.12 (m, 1H), −0.28 to −0.30 (m, 1H); ESI-MS: *m/z* = (M + H)^+^ 455.16.

### Synthesis of compound-2

#### Introduction

Synthesis of **compound-2** was achieved from earlier synthesized intermediate **H-8**. **H-8** was reacted with **H-11** in sealed tube to achieve **H-12**. **H-12** was treated with HCl to afford **H-13**. **H-13** was treated with propionaldehyde to achieve **H-14**. **H-14** was treated with TiCl_4_ to afford **compound-2**. Supplementary Fig. 18 shows the synthetic scheme.

#### Preparation of H-12

**H-8** (0.300 g, 0.525 mmol, AMRI lot # IN-GUM-D-17), CuI (50 mg, 0.262 mmol), Cs_2_CO_3_ (0.341 g, 1.050 mmol), 1,10-phenathroline (20.0 mg, 0.105 mmol) and **H-11** (0.363 g, 2.101 mmol) in butyronitrile (0.2 mL) was stirred at 130 °C for 24 h in sealed tube. The progress of the reaction was monitored by TLC and UPLC-MS. The reaction mixture was cooled to room temperature and diluted with EtOAc (150 mL), copper salts were filtered through celite pad, washed with excess of ethylacetate (25 mL), filtrate was washed with sat. aq. NaHCO_3_ (2 × 50 ml), dried over Na_2_SO_4_, filtered and concentrated under reduced pressure to obtained crude material. The obtained crude material was purified by flash chromatography by using silica gel (100–200 mesh) and eluted with 15–20% EtOAc in hexanes. Combined column fractions were concentrated under reduced pressure to afford **H-12** [0.24 g, 75.0%, AMRI lot # IN-GUM-D-24] as a pale yellow oil.

^1^H NMR (400 MHz CDCl_3_): δ 7.48–7.45 (m, 4H), 7.43–7.30 (m, 8H), 7.04–7.00 (m, 2H), 6.88 (dd, *J* = 8.4 Hz, 2.0 Hz, 1H), 6.80 (bs, 1H), 6.62 (d, *J* = 8.8 Hz, 2H), 5.08 (s, 2H), 4.87–4.84 (m, 1H), 4.29–4.25 (m, 2H), 4.01–3.96 (m, 2H), 3.75–3.71 (m, 1H), 2.18–2.14 (m, 1H), 1.44 (s, 9H), 0.32–0.26 (bs, 3H), 0.02–0.05 (m, 1H); ESI-MS: *m/z* = (M + H)^+^ 617.24.

#### Preparation of H-13

**H-12** (0.250 g, 0.405 mmol, AMRI lot # IN-GUM-D-24) in dioxane (10.0 mL) was charged with HCl [1.0 mL (4.0 M in 1, 4-dioxane)] at 0 °C. The resulting reaction mixture was stirred at room temperature for 16 h. Reaction mixture was concentrated under reduced pressure to afford **H-13** [0.28 g (crude HCl salt), AMRI lot # IN-GUM-D-33] as a pale yellow oil; ESI-MS: *m/z* = (M + H)^+^ 517.18.

#### Preparation of H-14

**H-13** (0.280 g, 0.542 mmol, AMRI lot # IN-GUM-D-33) in MeOH (10.0 mL) was charged propionaldehyde (0.7 mL, 2.710 mmol) followed by acetic acid (0.1 mL) at room temperature under argon atmosphere. The resultant reaction mixture was stir for 4 h. NaBH_3_CN (0.10 g, 1.620 mmol) was added in two to three lots, stirring was continued for 16 h. Reaction mixture was diluted with CH_2_Cl_2_ (100 mL), washed the CH_2_Cl_2_ layer with sat. aq. NaHCO_3_ (2 × 50 mL), brine (2 × 50 mL), dried over Na_2_SO_4_, filtered and concentrated under reduced pressure to afford **H-14** [0.210 g (crude), AMRI lot # IN-GUM-D-34] as pale yellow semi-solid; ESI-MS: *m/z* = (M + H)^+^ 559.24.

#### Preparation of compound-2

**H-14** (0.260 g, 0.905 mmol, AMRI lot # IN-GUM-D-56) in CH_2_Cl_2_ (20.0 mL) was charged with TiCl_4_ (4.0 mL) at 0 °C. The resulting reaction mixture was stirred at the same temperature for 3 h. The progress of the reaction was monitored by UPLC. The reaction was quenched by pouring in ice cold NaHCO_3_ (100 mL), the resulted material was extracted with 10% MeOH in CH_2_Cl_2_ (3 × 100 mL). The combined organic layer was washed with sat.aq. NaHCO_3_ (3 × 50.0 mL), brine (2 × 30.0 mL), dried over Na_2_SO_4_, filtered, and concentrated under reduced pressure to obtain crude material. The obtained crude material was purified by preparative-HPLC (Method-A). The pure fractions were collected and CH_3_CN was concentrated under reduced pressure. Aqueous layer (10.0 mL) was extracted with 10% MeOH in CH_2_Cl_2_ (3 × 20 mL); organic layer was washed with sat. aq. NaHCO_3_ (10 mL), dried over Na_2_SO_4_, filtered, and concentrated and the resulted material was lyophilized to afford **compound**-**2** [0.038 g, 20.10% (over three steps) AMRI lot # IN-GUM-D-58] as an off-white solid.

^1^H NMR (400 MHz, DMSO): δ 9.42 (s, 1H), 7.45 (s, 5H), 7.15 (d, *J* = 8.4 Hz, 2H), 6.88 (d, *J* = 8.4 Hz, 1H), 6.74 (d, *J* = 8.8 Hz, 3H), 6.67–6.61 (m, 2H), 4.75–4.61 (m, 1H), 3.71–3.68 (m, 2H), 3.64–3.59 (m, 1H), 2.89–2.84 (m, 2H), 2.38–2.32 (m, 2H), 2.21–2.16 (m, 1H), 1.31–1.23 (m, 2H), 0.85–0.81 (m, 3H), 0.28–0.22 (bs, 2H), 0.14–0.10 (m, 1H), −0.28 to −0.31 (m, 1H); ESI-MS: *m/z* = (M + H)^+^ 469.69.

### Synthesis of compound-6

#### Introduction

Synthesis of **compound-6** was achieved from synthesized intermediate **H-8**. **H-8** was reacted with **H-15** in sealed tube to achieve **H-16**. **H-16** was treated with TiCl_4_ to afford **compound-6**. Supplementary Fig. 19 shows the synthetic scheme.

#### Preparation of H-16

**H-8** (0.20 g, 0.350 mmol, AMRI lot # IN-GUM-C-197), CuI (7.0 mg, 0.035 mmol), Cs_2_CO_3_ (0.227 g, 0.700 mmol), 1,10-phenathroline (14.0 mg, 0.070 mmol) and **H-15** (0.4 mL mmol) was stirred at 125 °C for 24 h in sealed tube. The progress of the reaction was monitored by TLC and UPLC-MS. Reaction mixture was cooled to room temperature and diluted with EtOAc (100 mL), copper salts were filtered through celite pad, washed the celite with excess of ethylacetate (50 mL), filtrate was washed with sat. aq. NaHCO_3_ (2 × 50 ml), dried over Na_2_SO_4_, filtered and concentrated under reduced pressure to afford **H-16** [0.21 g (crude), AMRI lot # IN-GUM-C-208] as an off white foam solid; ESI-MS: *m/z* = (M + H)^+^ 573.26.

#### Preparation of compound-6

**H-16** (0.210 g, 0.367 mmol, AMRI lot # IN-GUM-C-208) in CH_2_Cl_2_ (15.0 mL) was charged with TiCl_4_ (2.0 mL) at 0 °C. The resulting reaction mixture was stirred at the same temperature for 3 h. The reaction was quenched by pouring in ice cold sat. aq. NaHCO_3_ (100 mL), the resulted material was extracted with 10% MeOH in CH_2_Cl_2_ (3 × 150 mL). The combined organic layer was washed with sat. aq. NaHCO_3_ (2 × 50.0 mL), brine (2 × 50.0 mL), dried over Na_2_SO_4_, filtered, and concentrated under reduced pressure to obtain crude material as a pale yellow solid. The obtained crude material was purified by preparative-HPLC (Method-A). The pure fractions were collected, concentrated under reduced pressure, and the resulted material was lyophilized to afford **compound-6** [0.040 g, 23.80% (over two steps) AMRI lot # IN-GUM-D-1] as an off-white solid.

^1^H NMR (400 MHz, DMSO): δ 9.38 (s, 1H), 7.44 (s, 5H), 7.17 (d, *J* = 8.0 Hz, 2H), 6.87–6.77 (m, 4H), 6.67–6.62 (m, 2H), 4.07 (bs, 2H), 3.64–3.60 (m, 1H), 2.95–2.67 (m, 2H), 2.45–2.33 (m, 4H), 2.21–2.17 (m, 1H), 1.53–1.40 (m, 6H), 0.28–0.25 (bs, 2H), 0.14–0.12 (m, 1H), −0.25 to −0.27 (m, 1H); ESI-MS: *m/z* = (M + H)^+^ 483.21.

### Synthesis of compound-13

#### Introduction

Synthesis of **compound-13** was achieved from synthesized intermediate **H-8**. **H-8** was reacted with **H-17** in sealed tube to achieve **H-18**. **H-18** was treated with TiCl_4_ to afford **compound-13**. Supplementary Fig. 20 shows the scheme.

#### Preparation of H-18

**H-8** (1.00 g, 1.750 mmol, AMRI lot # IN-GUM-D-74), CuI (33.0 mg, 0.170 mmol), Cs_2_CO_3_ (1.130 g, 3.50 mmol), 1,10-phenathroline (70.0 mg, 0.035 mmol) and **H-17** (0.70 g, 5.250 mmol) in butyronitrile (3.0 mL) was stirred at 130 °C for 24 h in sealed tube. The progress of the reaction was monitored by TLC and UPLC-MS. Reaction mixture was cooled to room temperature and diluted with EtOAc (250 mL), copper salts were filtered through celite pad, washed with excess of ethylacetate (50 mL), filtrate was washed with sat. aq. NaHCO_3_ (2 × 50 ml), dried over Na_2_SO_4_, filtered and concentrated under reduced pressure to afford **H-18** [0.980 g (crude), AMRI lot # IN-GUM-D-84] as a brown oil; ESI-MS: *m/z* = (M + H)^+^ 573.22.

#### Preparation of compound-13

**H-18** (0.90 g, 1.573 mmol, AMRI lot # IN-GUM-D-84) in CH_2_Cl_2_ (50.0 mL) was charged TiCl_4_ (10.0 mL) at 0 °C under argon atmosphere. The resulting reaction mixture was stirred at the same temperature for 4 h. The progress of the reaction was monitored by UPLC. The reaction was quenched by pouring in ice cold sat. aq. NaHCO_3_ (250 mL), the resulted material was extracted with 10% MeOH in CH_2_Cl_2_ (3 × 150 mL). The combined organic layer was washed with sat. aq. NaHCO_3_ (2 × 50.0 mL), brine (2 × 50.0 mL), dried over Na_2_SO_4_, filtered, and concentrated under reduced pressure to obtain crude material as a pale yellow solid. The obtained crude material was purified by preparative-HPLC (Method-A). The pure fractions were collected and CH_3_CN was concentrated under reduced pressure. Aqueous layer (10.0 mL) was extracted with 10% MeOH in CH_2_Cl_2_ (3 × 35 mL); organic layer was washed with sat. aq. NaHCO_3_ (25 mL), dried over Na_2_SO_4_, filtered, and concentrated and the resulted material was lyophilized to afford **compound**-**13** [0.160 g, 18.95% (over two steps), AMRI lot # IN-GUM-D-88] as an off-white solid.

^1^H NMR (400 MHz, DMSO): δ 9.41 (s, 1H), 7.44 (s, 5H), 7.15 (d, *J* = 8.4 Hz, 2H), 6.89–6.78 (m, 4H), 6.67–6.62 (m, 2H), 3.80 (d, *J* = 6.8 Hz, 2H), 3.64–3.60 (m, 1H), 2.60–2.53 (m, 1H), 2.45–2.28 (m, 6H), 2.21–2.17 (m, 1H), 1.92–1.88 (m, 1H), 1.49–1.41 (m, 1H), 1.02–0.98 (m, 3H), 0.27–0.24 (bs, 2H), 0.14–0.06 (m, 1H), −0.26 to −0.28 (m, 1H); ESI-MS: *m/z* = (M + H)^+^ 483.17.

### Synthesis of compound-14

#### Introduction

Synthesis of **compound-14** was achieved from synthesized intermediate **H-8**. **H-8** was reacted with **H-19** in sealed tube to achieved **H-20**. **H-20** was treated with TiCl_4_ to afford **compound-14**. Supplementary Fig. 21 shows the scheme.

#### Preparation of H-20

**H-8** (1.00 g, 1.750 mmol, AMRI lot # IN-GUM-D-74), CuI (33.0 mg, 0.170 mmol), Cs_2_CO_3_ (1.130 g, 3.50 mmol), 1,10-phenathroline (70.0 mg, 0.035 mmol) and **H-19** (1.00 g, 7.00 mmol) in butyronitrile (3.0 mL) was stirred at 130 °C for 24 h in sealed tube. The progress of the reaction was monitored by TLC and UPLC-MS. The reaction mixture was cooled to room temperature and diluted with EtOAc (250 mL), copper salts were filtered through celite pad, washed with excess of ethylacetate (50 mL), filtrate was washed with sat. aq. NaHCO_3_ (3 × 50 ml), dried over Na_2_SO_4_, filtered and concentrated under reduced pressure to afford **H-20** [1.2 g (crude), AMRI lot # IN-GUM-D-87] as a brown oil; ESI-MS: *m/z* = (M + H)^+^ 587.21.

#### Preparation of compound-14

**H-20** (1.1 g, 1.877 mmol, AMRI lot # IN-GUM-D-87) in CH_2_Cl_2_ (50.0 mL) was charged TiCl_4_ (10.0 mL) at 0 °C under argon atmosphere. The resulting reaction mixture was stirred at the same temperature for 4 h. The progress of the reaction was monitored by UPLC. The reaction was quenched by pouring in ice cold sat. aq. NaHCO_3_ (350 mL), the resulted material was extracted with 10% MeOH in CH_2_Cl_2_ (3 × 200 mL). The combined organic layer was washed with sat. aq. NaHCO_3_ (2 × 100.0 mL), brine (2 × 100.0 mL), dried over Na_2_SO_4_, filtered, and concentrated under reduced pressure to obtain crude material as a pale yellow solid. The obtained crude material was purified by preparative-HPLC (Method-A). The pure fractions were collected and CH_3_CN was concentrated under reduced pressure. Aqueous layer (10.0 mL) was extracted with 10% MeOH in CH_2_Cl_2_ (3 × 35 mL); organic layer was washed with sat. aq. NaHCO_3_ (25 mL), dried over Na_2_SO_4_, filtered, and concentrated and the resulted material was lyophilized to afford **compound**-**14** [0.160 g, 18.47% (over two steps), AMRI lot # IN-GUM-D-89] as an off-white solid.

^1^H NMR (400 MHz, DMSO): δ 9.43 (bs, 1H), 7.44 (s, 5H), 7.15 (d, *J* = 8.4 Hz, 2H), 6.89–6.78 (m, 4H), 6.67–6.61 (m, 2H), 3.80 (d, *J* = 6.8 Hz, 2H), 3.64–3.60 (m, 1H), 2.60–2.53 (m, 1H), 2.45–2.28 (m, 6H), 2.21–2.17 (m, 1H), 1.92–1.88 (m, 1H), 1.49–1.37 (m, 3H), 0.86–0.83 (m, 3H), 0.28–0.21 (bs, 2H), 0.14–0.06 (m, 1H), −0.26 to −0.28 (m, 1H); ESI-MS: *m/z* = (M + H)^+^ 497.20.

### Synthesis of compound-16-Peak-1 and Peak-2

#### Introduction

Synthesis of **compound-16-Peak-1** and **Peak-2** were achieved from earlier synthesized intermediate **H-8**. **H-8** was reacted with **H-21** in sealed tube to achieve **H-22**. **H-22** was treated with TiCl_4_ to afford **compound-16-Peak-1** and **Peak-2**. Supplementary Fig. 22 shows the synthetic scheme.

#### Preparation of H-22

**H-8** (0.50 g, 0.875 mmol, AMRI lot # IN-GUM-D-17), CuI (20.0 mg, 0.087 mmol), Cs_2_CO_3_ (0.570 g, 3.50 mmol), 1,10-phenathroline (40.0 mg, 0.035 mmol) and **H-21** (0.5 mL) in butyronitrile (0.5 mL) was stirred at 130 °C for 24 h in sealed tube. The progress of the reaction was monitored by TLC. The reaction mixture was cooled to room temperature and diluted with EtOAc (150 mL), copper salts were filtered through celite pad, washed with excess of ethylacetate (50 mL), and filtrate was washed with sat. aq. NaHCO_3_ (3 × 50 ml), dried over Na_2_SO_4_, filtered and concentrated under reduced pressure to obtain crude material. The obtained crude was purified by flash chromatography by using silica gel (100–200 mesh) and eluted with 2–% MeOH in CH_2_Cl_2_. Combined pure column fractions were concentrated under reduced pressure to afford **H-22** [0.290 g, 58.00%, AMRI lot # IN-GUM-D-47] as a pale yellow semi-solid.

^1^H NMR (400 MHz, DMSO): δ 7.47–7.26 (m, 12H), 7.03 (d, *J* = 8.0 Hz, 2H), 6.89–6.87 (m, 1H), 6.80–6.78 (m, 3H), 4.09 (t, *J* = 6.0 Hz, 2H), 3.75–3.70 (m, 2H), 3.01–2.85 (m, 4H), 2.61–2.55 (m, 1H), 2.32–2.25 (m, 1H), 2.18–2.00 (m, 4H), 1.42–1.33 (m, 1H), 1.04 (d, *J* = 6.8 Hz, 3H), 0.28–0.26 (bs, 3H), 0.08 to −0.10 (bs, 1H); ESI-MS: *m/z* = (M + H)^+^ 573.23.

#### Preparation of Compound-16-Peak-1 and Peak-2

**H-22** (0.290 g, 0.506 mmol, AMRI lot # IN-GUM-D-47) in CH_2_Cl_2_ (30.0 mL) was charged TiCl_4_ (10.0 mL) at 0 °C under argon atmosphere. The resulting reaction mixture was stirred at the same temperature for 3 h. The progress of the reaction was monitored by UPLC. The reaction was quenched by pouring in ice cold sat. aq. NaHCO_3_ (150 mL), the resulted material was extracted with 10% MeOH in CH_2_Cl_2_ (3 × 150 mL). The combined organic layer was washed with sat. aq. NaHCO_3_ (2 × 50.0 mL), brine (2 × 50.0 mL), dried over Na_2_SO_4_, filtered, and concentrated under reduced pressure to obtain crude material as a pale yellow solid. The obtained crude material was purified by preparative-HPLC (Method-A). The pure fractions were collected and CH_3_CN was concentrated under reduced pressure. Aqueous layer (10.0 mL) was extracted with 10% MeOH in CH_2_Cl_2_ (3 × 15 mL); organic layer was washed with sat. aq. NaHCO_3_ (15 mL), dried over Na_2_SO_4_, filtered, and concentrated and the resulted material was lyophilized to afford **compound**-**16** [0.070 g, 16.58% (over two steps), AMRI lot # IN-GUM-D-49] as an off-white solid.

**Compound-16** (0.070 g) was purified by SFC (Method-B) to afford **Compound**-**16-Peak-1 (**0.020 g, 57.14%, AMRI lot # IN-GUM-D-67-Peak-1) as an off-white solid.

^1^H NMR (400 MHz, DMSO): δ 9.37 (s, 1H), 7.44 (s, 5H), 7.15 (d, *J* = 8.4 Hz, 2H), 6.89–6.83 (m, 3H), 6.81–6.763 (bs, 1H), 6.67–6.62 (m, 2H), 4.00 (t, *J* = 6.0 Hz, 2H), 3.64–3.59 (m, 1H), 2.80–2.70 (m, 3H), 2.67–2.54 (m, 2H), 2.21–2.11 (m, 2H), 2.06–2.02 (m, 1H), 1.94–1.88 (m, 1H), 1.27–1.19 (m, 1H), 0.96 (d, *J* = 6.8 Hz, 3H), 0.28–0.21 (bs, 2H), 0.14–0.12 (m, 1H), −0.25 to −0.28 (m, 1H); ESI-MS: *m/z* = (M + H)^+^ 483.21.

**Compound**-**16-Peak-2** [0.024 g, 68.57%, AMRI lot # IN-GUM-D-67-Peak-2] as an off-white solid.

^1^H NMR (400 MHz, DMSO): δ 9.38 (s, 1H), 7.44 (s, 5H), 7.15 (d, *J* = 8.4 Hz, 2H), 6.89–6.83 (m, 3H), 6.81–6.76 (bs, 1H), 6.67–6.62 (m, 2H), 4.00 (t, *J* = 6.0 Hz, 2H), 3.64–3.59 (m, 1H), 2.80–2.70 (m, 3H), 2.67–2.54 (m, 2H), 2.21–2.11 (m, 2H), 2.06–2.02 (m, 1H), 1.94–1.88 (m, 1H), 1.27–1.19 (m, 1H), 0.96 (d, *J* = 6.8 Hz, 3H), 0.28–0.21 (bs, 2H), 0.14–0.12 (m, 1H), −0.25 to −0.28 (m, 1H); ESI-MS: *m/z* = (M + H)^+^ 483.21.

### Synthesis of compound-3

#### Introduction

Synthesis of **compound-3** was achieved from earlier synthesized intermediate **H-6**. **H-6** was reacted with **H-9** in a sealed tube to achieve **H-10**. **H-10** was treated with TMS-Cl to afford **H-11**. **H-11** was treated with **H-12** to achieve **H-13**. **H-13** was treated with TiCl_4_ to afford

**Compound-3**. Supplementary Fig. 23 shows the synthetic scheme.

#### Preparation of H-10

To a stirred solution of **H-6** (0.80 g, 1.616 mmol, AMRI lot # IN-GUM-D-147), CuI (30.0 mg, 0.161 mmol), Cs_2_CO_3_ (1.05 g, 3.232 mmol), 1,10-phenathroline (58.0 mg, 0.323 mmol) and **H-9** (1.20 g, 6.464 mmol) was added butyronitrile (2.0 mL) and stirred at 130 °C for 24 h in a sealed tube. The progress of the reaction was monitored by TLC and UPLC-MS. Upon completion reaction mixture was cooled to room temperature and diluted with EtOAc (250 mL), copper salts were filtered through celite pad, washed with excess of EtOAc (100 mL), filtrate was concentrated under reduced pressure, the obtained crude material was purified by flash chromatography by using silica gel (100–200 mesh) and eluted with 30–35% EtOAc in hexanes. Pure column fractions were concentrated under reduced pressure to afford **H-10** (0.68 g, 76%, AMRI lot # IN-GUM-D-171) as a pale yellow foam.

^1^H NMR (400 MHz, DMSO-*d*_*6*_): δ 7.49–7.40 (m, 5H), 7.33 (d, *J* = 8.4 Hz, 2H), 7.23 (d, *J* = 8.0 Hz, 1H), 6.88–6.80 (m, 5H), 4.07 (d, *J* = 6.4 Hz, 2H), 3.94 (brs, 2H), 3.78 (s, 3H), 3.68 (brd, *J* = 17.4 Hz, 3H), 2.93–2.90 (m, 1H), 2.27 (brd, *J* = 17.4 Hz, 1H), 1.37 (s, 9H), 0.29 (brs, 2H), 0.14 (brs, 1H), −0.24 (d, *J* = 10.8 Hz, 1H); ESI-MS: *m/z* = (M + H)^+^ 555.51.

#### Preparation of H-11

To a stirred solution of **H-10** (0.65 g, 1.173 mmol, AMRI lot # IN-GUM-D-171) in 2, 2, 2, trifluoroethanol (7.00 mL) was added TMS-Cl (1.50 mL) at 0 °C. The resulting reaction mixture was stirred at room temperature for 5 h. Upon completion reaction, mixture was concentrated under reduced pressure to afford H**-11** [0.620 g (crude HCl salt), AMRI lot # IN-GUM-D-178] as an off-white solid.

ESI-MS: *m/z* = (M + H)^+^ 455.44

#### Preparation of H-13

To a stirred solution of **H-11** (0.12 g, 0.245 mmol, AMRI lot # IN-GUM-D-178) in MeOH (3.0 mL) was charged with **H-12** (3.0 mL) followed by the addition of acetic acid (0.5 mL) at room temperature under argon atmosphere. The resultant reaction mixture was stir for 4 h. NaBH_3_CN (0.10 g, 1.620 mmol) was added to the reaction mixture in two to three lots, and stirring was continued for 3 d. The reaction mixture was diluted with 10% MeOH/ CH_2_Cl_2_ (100 mL), organic layer washed with sat. aq. NaHCO_3_ (2 × 20.0 mL), brine (2 × 20.0 mL), dried over Na_2_SO_4_, filtered and concentrated under reduced pressure to afford **H-13** [0.120 g (crude), AMRI lot # IN-GUM-D-179] as an off-white semisolid.

ESI-MS: *m/z* = (M + H)^+^ 483.51

#### Preparation of compound-3

To a stirred solution of **H-12** (0.120 g, 0.248 mmol, AMRI lot # IN-GUM-D-179) in CH_2_Cl_2_ (5.0 mL) was charged with BBr_3_ (0.40 mL, 0.373 mmol, 1.0 M in CH_2_Cl_2_) at 0 °C and the reaction mixture was stirred at room temperature for 5 h. The progress of the reaction was monitored by UPLC. The reaction was quenched with MeOH (0.50 mL), the resulted material was extracted with 10% MeOH in CH_2_Cl_2_ (3 × 25 mL). The combined organic layers were washed with saturated aqueous NaHCO_3_ (2 × 10.0 mL), brine (2 × 10.0 mL), dried over Na_2_SO_4_, filtered, and concentrated under reduced pressure, the obtained crude material was purified by preparative-HPLC (Method-A). The pure fractions were collected and CH_3_CN was concentrated under reduced pressure. Aqueous layer (10.0 mL) was extracted with 10% MeOH in CH_2_Cl_2_ (3 × 20.0 mL), dried over Na_2_SO_4_, filtered, and concentrated to afford **compound-3** [0.011 g, 10.10% (over two steps) AMRI lot # IN-GUM-D-182] as an off-white solid.

^1^H NMR (400 MHz, DMSO-*d*_*6*_): δ 9.38 (s, 1H), 7.51–7.42 (m, 5H), 7.15 (d, *J* = 8.8 Hz, 2H), 6.89–6.76 (m, 4H), 6.67–6.62 (m, 2H), 4.04 (d, *J* = 7.2 Hz, 2H), 3.61 (d, *J* = 17.2 Hz, 1H), 3.23 (d, *J* = 6.8 Hz, 2H), 2.90 (brs, 2H), 2.74–2.66 (m, 3H), 2.19 (d, *J* = 16.8 Hz, 1H), 0.84 (t, *J* = 7.2 Hz, 3H), 0.27 (brs, 2H), 0.14–0.05 (m, 1H), -0.26 (brd, *J* = 11.2 Hz, 1H); ESI-MS: *m/z* = (M + H)^+^ 469.46.

### Synthesis of compound-4

#### Introduction

Synthesis of **compound-4** was achieved from earlier synthesized intermediate **H-8**. **H-8** was reacted with **H-9** under Ullmann reaction conditions to achieve **H-14**. **H-14** was treated for Boc deprotection with TMS-Cl to afford **H-15**. **H-15** on reductive amination with propionaldehyde to achieve **H-16**. **H-16** was treated for debenzylation with TiCl_4_ to afford **compound-4**. Supplementary Fig. 24 shows the synthetic scheme.

#### Preparation of H-14

To a stirred solution of **H-8** (0.80 g, 1.401 mmol, AMRI lot # IN-GUM-D-107), CuI (27.0 mg, 0.140 mmol), Cs_2_CO_3_ (0.910 g, 2.802 mmol), 1,10-phenathroline (52.0 mg, 0.280 mmol) and **H-9** (1.04 g, 5.604 mmol) in butyronitrile (2.0 mL) was stirred at 130 °C for 24 h in a sealed tube. The progress of the reaction was monitored by TLC and UPLC-MS. Upon reaction mixture was cooled to room temperature and diluted with EtOAc (250 mL), copper salts were filtered through celite pad, washed with excess of EtOAc (100 mL), filtrate was concentrated under reduced pressure, the obtained crude material was purified by flash chromatography by using silica gel (100–200 mesh) and eluted with 30–35% EtOAc in hexanes. Combined column fractions were concentrated under reduced pressure to afford **H-14** (0.550 g, 62.0%, AMRI lot # IN-GUM-D-133) as a pale yellow foam solid.

^1^H NMR (400 MHz, DMSO): δ 7.49–7.33 (m, 10H), 7.17 (d, *J* = 8.4 Hz, 2H), 7.02 (d, *J* = 8.4 Hz, 1H), 6.93–6.86 (m, 5H), 5.12 (s, 2H), 4.07 (d, *J* = 6.8 Hz, 2H), 3.94 (brs, 2H), 3.68 (d, *J* = 17.4 Hz, 3H), 2.95–2.88 (m, 1H), 2.27 (brd, *J* = 16.8 Hz, 1H), 1.37 (s, 9H), 0.29 (brs, 2H), 0.14 (brs, 1H), -0.24 (d, *J* = 10.8 Hz, 1H).

#### Preparation of H-15

To a stirred solution of **H-14** (0.550 g, 0.873 mmol, AMRI lot # IN-GUM-D-133) in trifluoroethanol(10.0 mL) was charged with TMS-Cl (1.00 mL) at 0 °C. The resulting reaction mixture was stirred at room temperature for 4 h. The reaction mixture was concentrated under reduced pressure to afford **H-15** [0.50 g (crude HCl salt), AMRI lot # IN-GUM-D-136] as an off white solid; ESI-MS: *m/z* = (M + H)^+^ 531.12.

#### Preparation of H-16

To a stirred solution of **H-15** (0.35 g, 0.620 mmol, AMRI lot # IN-GUM-D-136) in MeOH (15.0 mL) was charged with propionaldehyde (0.2 mL, 1.551 mmol) followed by the addition of acetic acid (0.1 mL) at room temperature under argon atmosphere and the reaction mixture was stir for 4 h, NaBH_3_CN (0.15 g, 1.556 mmol) was added to the reaction mixture in two to three lots and stirring was continued for 24 h. The reaction mixture was diluted with CH_2_Cl_2_ (100 mL) washed with saturated aqueous NaHCO_3_ (2 × 50.0 mL), brine (2 × 50.0 mL), dried over Na_2_SO_4_, filtered and concentrated under reduced pressure to afford **H-16** [0.370 g (crude), AMRI lot # IN-GUM-D-137] as pale yellow semi-solid; ESI-MS: *m/z* = (M + H)^+^ 573.19.

#### Preparation of compound-4

To a stirred solution of **H-16** (0.350 g, 0.580 mmol, AMRI lot # IN-GUM-D-137) in CH_2_Cl_2_ (30.0 mL) was charged with TiCl_4_ (4.00 mL) at 0 °C and the resulting reaction mixture was stirred at the same temperature for 4 h. The progress of the reaction was monitored by UPLC-MS, upon completion reaction mixture was quenched by pouring in ice cold NaHCO3 (150 mL), the resulted solution was extracted with 10% MeOH in CH_2_Cl_2_ (3 × 150 mL). The combined organic layer was washed with sat. aq. NaHCO_3_ (2 × 50.0 mL), brine (2 × 50.0 mL), dried over Na_2_SO_4_, filtered and concentrated under reduced pressure. The obtained crude material was purified by preparative-HPLC (Method-A). The pure fractions were collected and CH_3_CN was concentrated under reduced pressure and aqueous layer (10.0 mL) was extracted with 10% MeOH in CH_2_Cl_2_ (3 × 20.0 mL), organic layer was washed with sat. aq. NaHCO_3_ (10.0 mL), dried over Na_2_SO_4_, filtered, and concentrated and the resulted material was lyophilized to afford **compound**-**4** [0.060 g, 20.10% (over three steps) AMRI lot # IN-GUM-D-141] as an off-white solid.

^1^H NMR (400 MHz, DMSO-*d*_*6*_): δ 9.41 (s, 1H), 7.49–7.40 (m, 5H), 7.16 (d, *J* = 8.4 Hz, 2H), 6.89–6.78 (m, 4H), 6.67–6.62 (m, 2H), 4.04 (d, *J* = 6.8 Hz, 2H), 3.62 (d, *J* = 16.8 Hz, 1H), 3.28 (d, *J* = 6.4 Hz, 2H), 2.93 (brs, 2H), 2.78–2.71 (m, 1H), 2.34–2.21 (m, 2H), 2.19 (d, *J* = 16.8 Hz, 1H), 1.25 (q, *J* = 7.6 Hz, 2H), 0.82 (t, *J* = 7.2 Hz, 3H), 0.27 (brs, 2H), 0.14–0.10 (m, 1H), −0.28 (brd, *J* = 10.8 Hz, 1H); ESI-MS: *m/z* = (M + H)^+^ 483.16.

### Synthesis of compound-5

#### Introduction

Synthesis of **compound-5** was achieved from synthesized intermediate **H-7**. **H-7** was reacted with TBDMS-Cl to afford **H-17**. **H-17** was reacted with **H-18** in a sealed tube to afford **compound-5**. Supplementary Fig. 25 shows the synthetic scheme.

#### Preparation of H-17

To a stirred solution of **H-7** [2.00 g, 4.150 mmol, AMRI lot # IN-GUM-C-169] in CH_2_Cl_2_ (50.0 mL) was charged with imidazole (0.60 g, 8.300 mmol) followed by the addition of TBDMS-Cl (1.00 g, 6.230 mmol) at 0 °C, under argon atmosphere. The reaction mixture was stirred for 48 h, upon completion, reaction mixture was diluted with CH_2_Cl_2_ (100 mL) and the organic layer was washed with water (2 × 50.0 mL), brine (2 × 50.0 mL). The organic layer was dried over Na_2_SO_4_, filtered and concentrated under reduced pressure; the obtained crude material was purified by flash chromatography by using silica gel (100–200 mesh) and eluted with 2–5% EtOAc in hexanes. The pure column fractions were concentrated under reduced pressure to afford **H-17** (1.75 g, 70.85%, AMRI lot # IN-GUM-C-170) as an off-white foam solid.

^1^H NMR (400 MHz, CDCl_3_): δ 7.48 (d, *J* = 8.4 Hz, 2H), 7.38–7.29 (m, 5H), 7.05 (d, *J* = 8.4 Hz, 2H), 6.93–6.86 (m, 1H), 6.84 (d, *J* = 8.4 Hz, 1H), 6.63 (dd, *J* = 8.4 Hz, 2.0 Hz, 1H), 6.57(s, 1H), 3.60 (d, *J* = 16.8 Hz,1H), 2.03 (d, *J* = 16.8 Hz, 1H), 0.91 (s, 9H), 0.18 (brs, 2H), 0.14 (s, 6H), 0.09 (s, 1H), −0.14 to −018 (m, 1H);

#### Preparation of compound-5

To a stirred solution of **H-17** (0.25 g, 0.420 mmol, AMRI lot # IN-GUM-C-170), CuI (8.0 mg, 0.042 mmol), Cs_2_CO_3_ (0.273 g, 0.840 mmol), 1,10-phenathroline (15.0 mg, 0.084 mmol) in **H-18** (1.00 mL) was stirred at 125 °C for 24 h in a sealed tube. The progress of the reaction was monitored by TLC and UPLC-MS, upon completion reaction mixture was cooled to room temperature and diluted with 10% MeOH/CH_2_Cl_2_ (50 mL), copper salts were filtered through celite pad, washed the celite with excess of 10% MeOH/CH_2_Cl_2_ (50.0 mL), filtrate was washed with sat. aq. NaHCO_3_ (2 × 20.0 ml), dried over Na_2_SO_4_, filtered and concentrated under reduced pressure, the obtained crude material was purified by preparative-HPLC (Method-A). The pure fractions were collected, concentrated under reduced pressure, and the resulted material was lyophilized to afford **compound-5** [0.028 g, 23.8% (over two steps) AMRI lot # IN-GUM-C-171] as an off-white solid.

^1^H NMR (400 MHz, CDCl_3_): δ 7.47–7.38 (m, 5H), 7.26 (d, *J* = 8.0 Hz, 2H), 6.98–6.89 (m, 1H), 6.73–6.66 (m, 4H), 4.22 (brs, 2H), 3.66 (d, *J* = 16.4 Hz, 1H),3.14 (s, 2H), 3.02 (s, 3H), 2.09 (d, *J* = 16.4 Hz, 1H) 1.81 (s, 4H), 1.66 (s, 5H), 0.89–0.83 (m, 2H), 0.28–0.19 (m, 3H), −0.05 to −0.14 (m, 1H); ESI-MS: *m/z* = (M + H)^+^ 497.37.

### Synthesis of compound-12

#### Introduction

Synthesis of **compound-12** was achieved from synthesized intermediate **H-8**. **H-8** was reacted with **H-19** in a sealed tube to achieve **H-20**. **H-20** was treated with TiCl_4_ to afford **compound-13**. Supplementary Fig. 26 shows the synthetic scheme.

#### Preparation of H-20

To a stirred solution of **H-8** (0.40 g, 0.700 mmol, AMRI lot # IN-GUM-D-64), CuI (70.0 mg, 0.350 mmol), Cs_2_CO_3_ (0.455 g, 1.400 mmol), 1,10-phenathroline (30.0 mg, 0.140 mmol) and **H-19** (0.5 g, 2.802 mmol) in butyronitrile (1.00 mL) was stirred at 130 °C for 24 h in a sealed tube. The progress of the reaction was monitored by TLC and UPLC-MS, upon completion, reaction mixture was cooled to room temperature and diluted with EtOAc (250 mL), copper salts were filtered through celite pad, washed with excess of EtOAc (50.0 mL), filtrate was washed with saturated aqueous NaHCO_3_ (2 × 50.0 mL), dried over Na_2_SO_4_, filtered and concentrated under reduced pressure. The obtained crude material was purified by flash chromatography by using silica gel (100–200 mesh) and eluted with 7–10% MeOH in CH_2_Cl_2_. The pure column fractions were concentrated under reduced pressure to afford **H-20** (0.135 g, 34.85%, AMRI lot # IN-GUM-D-65) as a pale brown foam solid.

ESI-MS: *m/z* = (M + H)^+^ 559.21.

#### Preparation of compound-12

To a stirred solution of **H-20** (0.130 g, 0.232 mmol, AMRI lot # IN-GUM-D-65) in CH_2_Cl_2_ (20.0 mL) was charged TiCl_4_ (2.5 mL) at 0 °C under argon atmosphere and the resulting reaction mixture was stirred at the same temperature for 4 h. The progress of the reaction was monitored by UPLC upon completion; the reaction was quenched by pouring in ice cold sat. aq. NaHCO_3_ (50 mL), the resulted solution was extracted with 10% MeOH in CH_2_Cl_2_ (3 × 100 mL). The combined organic layer was washed with sat. aq. NaHCO_3_ (2 × 50.0 mL), brine (2 × 50.0 mL), dried over Na_2_SO_4_, filtered, and concentrated under reduced pressure to obtain crude material as a pale yellow solid. The obtained crude material was purified by preparative-HPLC (Method-A). The pure fractions were collected and CH_3_CN was concentrated under reduced pressure. Aqueous layer (10.0 mL) was extracted with 10% MeOH in CH_2_Cl_2_ (3 × 35.0 mL), organic layer was washed with saturated aqueous NaHCO_3_ (25.0 mL), dried over Na_2_SO_4_, filtered, and concentrated and the resulted material was lyophilized to afford **compound**-**12** (0.045 g, 37%, AMRI lot # IN-GUM-D-68] as an off-white solid.

^1^H NMR (400 MHz, DMSO-*d*_*6*_): δ 9.41 (s, 1H), 7.51–7.41(m, 5H), 7.14 (d, *J* = 8.4 Hz, 2H), 6.89 (d, *J* = 8.4 Hz, 1H), 6.79 (d, *J* = 8.4 Hz, 3H), 6.67–6.62 (m, 2H), 4.83–4.80 (m, 1H), 3.62 (d, *J* = 16.8 Hz, 1H), 2.81–2.77 (m, 1H), 2.67–2.60 (m, 1H), 2.45–2.39 (m, 4H), 2.26–2.23 (m, 1H), 2.19 (d, *J* = 16.8 Hz, 1H), 1.75–1.64 (m, 1H), 1.01 (t, *J* = 7.2 Hz, 3H), 0.27 (brs, 2H), 0.14–0.11 (m, 1H), −0.27 to −0.29 (m, 1H); ESI-MS: *m/z* = (M + H)^+^ 469.19.

### Synthesis of compound-15-Peak-1 and Peak-2

#### Introduction

Synthesis of **compound-15-Peak-1** and **Peak-2** were achieved from earlier synthesized intermediate **H-8 & H-22**. **H-8** was reacted with **H-20** under Ullmann reaction conditions to achieve **H-23**. **H-23** upon debenzylation with TiCl_4_ to afford **compound-15**. The obtained **Compound-15** was subjected to diastereomeric separation using SFC to get **Compound-15-Peak-1** and **Compound-15-Peak-2**. Supplementary Fig. 27 shows the synthetic scheme.

#### Preparation of H-22

To a stirred solution of **H-21** (1.00 g, 8.220 mmol,), K_2_CO_3_ (2.30 g, 16.44 mmol), in acetonitrile (50.0 mL) was charged 2-bromoethanol (0.70 mL, 9.04 mmol). The resultant mixture was stirred at 80 °C for 24 h. The progress of the reaction was monitored by TLC and UPLC-MS. Upon completion the reaction mixture was cooled to room temperature and diluted with EtOAc (50 mL), potassium salts were filtered through celite pad, washed with excess of EtOAc (50.0 mL), filtrate was concentrated under reduced pressure to afford **H-22** [1.48 g (crude), AMRI lot # IN-GUM-D-51] as a brown oil.

#### Preparation of H-23

To a stirred solution of **H-8** (0.40 g, 0.700 mmol, AMRI lot # IN-GUM-D-55), CuI (14.0 mg, 0.070 mmol), Cs_2_CO_3_ (0.455 g, 1.400 mmol), 1,10-phenathroline (30.0 mg, 0.140 mmol) and **H-22** (0.5 g, 2.802 mmol) in butyronitrile (1.00 mL) was stirred at 130 °C for 24 h in a sealed tube. The progress of the reaction was monitored by TLC and UPLC-MS. Reaction mixture was cooled to room temperature and diluted with EtOAc (250 mL), copper salts were filtered through celite pad, washed with excess of EtOAc (50.0 mL), and filtrate was washed with saturated aqueous NaHCO_3_ (2 × 50.0 mL), dried over Na_2_SO_4_, filtered and concentrated under reduced pressure. The obtained crude material was purified by flash chromatography by using silica gel (100–200 mesh) and eluted with 3–5% MeOH in CH_2_Cl_2_. The pure column fractions were concentrated under reduced pressure to afford **H-23** (0.195 g, 48.75%, AMRI lot # IN-GUM-D-55) as a pale yellow oil.

ESI-MS: *m/z* = (M + H)^+^ 573.23.

#### Preparation of Compound-15-Peak-1 and Peak-2

To a stirred solution of **H-23** (0.195 g, 1.877 mmol, AMRI lot # IN-GUM-D-87) in CH_2_Cl_2_ (50.0 mL) was charged with TiCl_4_ (10.0 mL) at 0 °C under argon atmosphere and the resulting mixture was stirred at the same temperature for 4 h. The progress of the reaction was monitored by UPLC. The reaction mixture was quenched by pouring in ice-cold saturated aqueous NaHCO_3_ (350 mL), the resulted material was extracted with 10% MeOH in CH_2_Cl_2_ (3 × 200 mL). The combined organic layer was washed with saturated aqueous NaHCO_3_ (2 × 100 mL), brine (2 × 100 mL), dried over Na_2_SO_4_, filtered, and concentrated under reduced pressure to obtain crude material as a pale yellow solid. The obtained crude material was purified by preparative-HPLC (Method-A), pure fractions were collected and CH_3_CN was concentrated under reduced pressure. Aqueous layer (10.0 mL) was extracted with 10% MeOH in CH_2_Cl_2_ (3 × 35.0 mL), organic layer was washed with saturated aqueous NaHCO_3_ (25.0 mL), dried over Na_2_SO_4_, filtered, and concentrated to afford **compound**-**15 (**0.070 g, 48%, AMRI lot # IN-GUM-D-60] as an off-white solid.

**Compound-15** (0.070 g) was purified by SFC (Method-B) to afford **Compound**-**15-Peak-1** (0.024 g, 57.14%, AMRI lot # IN-GUM-D-66-Peak-1) as an off-white solid.

^1^H NMR (400 MHz, DMSO-*d*_*6*_): δ 9.43 (s, 1H), 7.49–7.40 (m, 5H), 7.15 (d, *J* = 8.8 Hz, 2H), 6.89–6.83 (m, 3H), 6.78 (brs, 1H), 6.70–6.62 (m, 2H), 4.00 (t, *J* = 6.0 Hz, 2H), 3.62 (d, *J* = 17.2 Hz, 1H), 2.80–2.60 (m, 4H), 2.59–2.44 (m, 1H), 2.19 (d, *J* = 17.2 Hz,1H), 2.14–2.11 (m, 1H), 2.05–2.01 (m, 1H), 1.93–1.88 (m, 1H), 1.26–1.20 (m, 1H), 0.96 (d, *J* = 6.8 Hz, 3H), 0.27 (brs, 2H), 0.14–0.12 (m, 1H), −0.25 to −0.28 (m, 1H); ESI-MS: *m/z* = (M + H)^+^ 483.21.

**Compound**-**15-Peak-2** [0.024 g, 68.57%, AMRI lot # IN-GUM-D-66-Peak-2] as an off-white solid.

^1^H NMR (400 MHz, DMSO-*d*_*6*_): δ 9.37 (s, 1H), 7.49–7.41 (m, 5H), 7.15 (d, *J* = 8.4 Hz, 2H), 6.89–6.83 (m, 3H), 6.76 (brs, 1H), 6.67–6.62 (m, 2H), 4.00 (t, *J* = 6.0 Hz, 2H), 3.62 (d, *J* = 17.2 Hz, 1H), 2.80–2.60 (m, 4H), 2.59–2.44 (m, 1H), 2.19 (d, *J* = 17.2 Hz,1H), 2.14–2.11 (m, 1H), 2.05–2.01 (m, 1H), 1.93–1.88 (m, 1H), 1.26–1.20 (m, 1H), 0.96 (d, *J* = 6.8 Hz, 3H), 0.27 (brs, 2H), 0.14–0.12 (m, 1H), −0.26 to −0.29 (m, 1H); ESI-MS: *m/z* = (M + H)^+^ 483.21.

### Synthesis of compound-17

#### Introduction

Synthesis of **compound-17** was achieved from earlier synthesized intermediate **H-8 & H-25**. **H-8** was reacted with **H-25** under Ullmann reaction conditions in a sealed tube to achieve **H-26**. **H-26** upon debenzylation with TiCl_4_ to afford **compound-17**. Supplementary Fig. 28 shows the synthetic scheme.

#### Preparation of H-25

To a stirred solution of **H-24** (5.00 g, 41.11 mmol,), K_2_CO_3_ (10.00 g, 82.22 mmol), in acetonitrile (75.0 mL) was charged 2-bromoethanol (3.40 mL, 45.23 mmol). The resultant reaction mixture was stirred at 85 °C for 16 h. The progress of the reaction was monitored by TLC. Reaction mixture was cooled to room temperature and diluted with EtOAc (50 mL), potassium salts were filtered through celite pad, washed with excess of EtOAc (50.0 mL), filtrate was concentrated under reduced pressure to afford **H-25** [6.30 g (crude), AMRI lot # IN-GUM-D-31] as a brown oil.

#### Preparation of H-26

To a stirred solution of **H-8** (0.50 g, 0.875 mmol, AMRI lot # IN-GUM-D-17), CuI (30.0 mg, 0.087 mmol), Cs_2_CO_3_ (0.570 g, 1.751 mmol), 1,10-phenathroline (32.0 mg, 0.175 mmol) and **H-25** (0.5 mL) in butyronitrile (1.00 mL) was stirred at 130 °C for 24 h in a sealed tube. The progress of the reaction was monitored by TLC and UPLC-MS. Upon completion reaction, mixture was cooled to room temperature and diluted with EtOAc (250 mL), copper salts were filtered through celite pad, washed with excess of EtOAc (50.0 mL), and filtrate was washed with saturated aqueous NaHCO_3_ (2 × 50.0 mL), dried over Na_2_SO_4_, filtered and concentrated under reduced pressure. The obtained crude material was purified by flash chromatography by using silica gel (100–200 mesh) and eluted with 3–5% MeOH in CH_2_Cl_2_. The combined column fractions were concentrated under reduced pressure to afford **H-26** (0.260 g, 51.91%, AMRI lot # IN-GUM-D-40) as a pale yellow oil.

ESI-MS: *m/z* = (M + H)^+^ 573.23.

#### Preparation of compound-17

To a stirred solution of **H-26** (0.30 g, 0.524 mmol, AMRI lot # IN-GUM-D-40) in CH_2_Cl_2_ (50.0 mL) was charged with TiCl_4_ (10.0 mL) at 0 °C under argon atmosphere and the resulting reaction mixture was stirred at the same temperature for 4 h. The progress of the reaction was monitored by UPLC. Upon completion, the reaction mixture was quenched by pouring in ice-cold saturated aqueous NaHCO_3_ (350 mL), the resulted material was extracted with 10% MeOH in CH_2_Cl_2_ (3 × 200 mL). The combined organic layers were washed with saturated aqueous NaHCO_3_ (2 × 100 mL), brine (2 × 100 mL), dried over Na_2_SO_4_, filtered, and concentrated under reduced pressure to obtain crude material as a pale yellow solid. The obtained crude material was purified by preparative-HPLC (Method-A), pure fractions were collected and CH_3_CN was concentrated under reduced pressure. Aqueous layer (10.0 mL) was extracted with 10% MeOH in CH_2_Cl_2_ (3 × 35.0 mL), organic layer was washed with saturated aqueous NaHCO_3_ (25.0 mL), dried over Na_2_SO_4_, filtered, and concentrated to afford **compound**-**17** (0.075 g, 48.00%, AMRI lot # IN-GUM-D-43] as an off-white solid.

^1^H NMR (400 MHz, DMSO-*d*_*6*_): δ 9.41 (s, 1H), 7.50–7.41 (m, 5H), 7.15 (d, *J* = 8.4 Hz, 2H), 6.90–6.83 (m, 3H), 6.78 (brs, 1H), 6.67–6.62 (m, 2H), 4.01 (t, *J* = 6.0 Hz, 2H), 3.62 (d, *J* = 16.8 Hz, 1H), 3.10–3.06 (m, 2H), 2.44–2.33 (m, 2H), 2.19 (d, *J* = 16.8 Hz,2H), 1.89–1.81 (m, 1H), 1.65–1.61 (m, 2H),1.29–1.23 (m, 1H), 1.02 (d, *J* = 6.0 Hz, 3H), 0.27 (brs, 2H), 0.14–0.11 (m, 1H), −0.27 to −0.29 (m, 1H); ESI-MS: *m/z* = (M + H)^+^ 483.2

### Synthesis of compound-18

#### Introduction

Synthesis of **compound-18** was achieved from earlier synthesized intermediate **H-8 & H-28**. **H-8** was reacted with **H-28** under Ullmann reaction conditions in a sealed tube to achieve **H-29**. **H-29** was treated for debenzylation with TiCl_4_ to afford **compound-18**. Supplementary Fig. 29 shows the synthetic scheme.

#### Preparation of H-28

To a stirred solution of **H-27** (3.50 g, 28.78 mmol,), K_2_CO_3_ (7.10 g, 57.56.22 mmol), in acetonitrile (50.0 mL) was charged 2-bromoethanol (2.40 mL, 31.65 mmol). The resultant reaction mixture was stirred at 85 °C for 16 h. The progress of the reaction was monitored by TLC. Upon completion reaction mixture was cooled to room temperature and diluted with EtOAc (50 mL), potassium salts were filtered through celite pad, washed with excess of EtOAc (50.0 mL), filtrate was concentrated under reduced pressure to afford **H-28** [4.60 g (crude), AMRI lot # IN-GUM-D-11] as a yellow oil.

#### Preparation of H-29

To a stirred solution of **H-8** (0.30 g, 0.525 mmol, AMRI lot # IN-GUM-D-17), CuI (10.0 mg, 0.052 mmol), Cs_2_CO_3_ (0.34 g, 1.050 mmol), 1,10-phenathroline (19.0 mg, 0.105 mmol) and **H-28** (0.5 mL) in butyronitrile (0.500 mL) was stirred at 130 °C for 24 h in a sealed tube. The progress of the reaction was monitored by TLC and UPLC-MS. Reaction mixture was cooled to room temperature and diluted with EtOAc (250 mL), copper salts were filtered through celite pad, washed with excess of EtOAc (50.0 mL), filtrate was washed with saturated aqueous NaHCO_3_ (2 × 50.0 mL), dried over Na_2_SO_4_, filtered and concentrated under reduced pressure. The obtained crude material was purified by flash chromatography by using silica gel (100–200 mesh) and eluted with 5–7% MeOH in CH_2_Cl_2_. The combined pure fractions were concentrated under reduced pressure to afford **H-29** (0.152 g, 51.91%, AMRI lot # IN-GUM-D-27) as a pale yellow oil.

ESI-MS: *m/z* = (M + H)^+^ 573.23.

#### Preparation of compound-18

To a stirred solution of **H-29** (0.220 g, 0.384 mmol, AMRI lot # IN-GUM-D-27) in CH_2_Cl_2_ (15.0 mL) was charged with TiCl_4_ (3.0 mL) at 0 °C under argon atmosphere and the resulting reaction mixture was stirred at the same temperature for 5 h. The progress of the reaction was monitored by UPLC. Upon completion, the reaction mixture was quenched by pouring in ice-cold saturated aqueous NaHCO_3_ (100 mL), the resulted material was extracted with 10% MeOH in CH_2_Cl_2_ (3 × 150 mL). The combined organic layer was washed with saturated aqueous NaHCO_3_ (2 × 50 mL), brine (2 × 50 mL), dried over Na_2_SO_4_, filtered, and concentrated under reduced pressure to obtain crude material as a pale yellow solid. The obtained crude material was purified by preparative-HPLC (Method-A), pure fractions were collected and CH_3_CN was concentrated under reduced pressure. Aqueous layer (10.0 mL) was extracted with 10% MeOH in CH_2_Cl_2_ (3 × 10.0 mL), organic layer was washed with saturated aqueous NaHCO_3_ (10.0 mL), dried over Na_2_SO_4_, filtered, and concentrated to afford **compound**-**18** (0.080 g, 41.00%, AMRI lot # IN-GUM-D-30] as a pale yellow solid.

^1^H NMR (400 MHz, DMSO-*d*_*6*_): δ 9.38 (s, 1H), 7.49–7.41(m, 5H), 7.15 (d, *J* = 8.4 Hz, 2H), 6.89–6.83 (m, 3H), 6.77 (brs, 1H), 6.67–6.62 (m, 2H), 4.01 (t, *J* = 6.0 Hz, 2H), 3.62 (d, *J* = 16.8 Hz, 1H), 3.13–3.06 (m, 2H), 2.44–2.32 (m, 2H), 2.19 (d, *J* = 16.8 Hz,2H), 1.89–1.81 (m, 1H), 1.65–1.61 (m, 2H),1.31–1.23 (m, 1H), 1.02 (d, *J* = 6.0 Hz, 3H), 0.27 (brs, 2H), 0.14–0.11 (m, 1H), −0.26 to −0.28 (m, 1H); ESI-MS: *m/z* = (M + H)^+^ 483.2.

### Synthesis of compound-19

#### Introduction

Synthesis of **compound-19** was achieved from synthesized intermediate **H-8**. **H-8** was reacted with **H-30** under Ullmann reaction conditions in a sealed tube to achieve **H-31**. **H-31** was treated for debenzylation with TiCl_4_ to afford **compound-19**. Supplementary Fig. 30 shows the synthetic scheme.

#### Preparation of H-31

To a stirred solution of **H-8** (0.30 g, 0.525 mmol, AMRI lot # IN-GUM-C-197), CuI (10.0 mg, 0.052 mmol), Cs_2_CO_3_ (0.340 g, 1.050 mmol), 1,10-phenathroline (20.0 mg, 0.105 mmol) and **H-30** (0.5 mL) was stirred at 130 °C for 24 h in a sealed tube. The progress of the reaction was monitored by TLC. Upon completion reaction, mixture was cooled to room temperature and diluted with EtOAc (150 mL), copper salts were filtered through celite pad, washed with excess of EtOAc (50.0 mL), filtrate was washed with sat. aq. NaHCO_3_ (3 × 50.0 ml), dried over Na_2_SO_4_, filtered and concentrated under reduced pressure to afford **H-31** [0.240 g, (crude), AMRI lot # IN-GUM-D-13] as a pale yellow oil.

ESI-MS: *m/z* = (M + H)^+^ 533.22.

#### Preparation of compound-19

To a stirred solution of **H-31** (0.240 g, 0.451 mmol, AMRI lot # IN-GUM-D-13) in CH_2_Cl_2_ (30.0 mL) was charged with TiCl_4_ (5.0 mL) at 0 °C under argon atmosphere and the resulting reaction mixture was stirred at the same temperature for 4 h. The progress of the reaction was monitored by UPLC. Upon completion the reaction was quenched by pouring in ice-cold sat. aq. NaHCO_3_ (150 mL), the resulted material was extracted with 10% MeOH in CH_2_Cl_2_ (3 × 150 mL). The combined organic layer was washed with sat. aq. NaHCO_3_ (2 × 50.0 mL), brine (2 × 50.0 mL), dried over Na_2_SO_4_, filtered, and concentrated under reduced pressure to obtain crude material as a pale yellow solid. The obtained crude material was purified by preparative-HPLC (Method-A). The pure fractions were collected and CH_3_CN was concentrated under reduced pressure. Aqueous layer (10.0 mL) was extracted with 10% MeOH in CH_2_Cl_2_ (3 × 15 mL); organic layer was washed with sat. aq. NaHCO_3_ (15.0 mL), dried over Na_2_SO_4_, filtered, and concentrated and the resulted material was lyophilized to afford **compound**-**19** [0.042 g, 18.21% (over two steps), AMRI lot # IN-GUM-D-16] as an off-white solid.

^1^H NMR (400 MHz, DMSO-*d*_*6*_): δ 9.41 (s, 1H), 7.50–7.41 (m, 5H), 7.18 (d, *J* = 8.4 Hz, 2H), 6.87 (d, *J* = 8.4 Hz, 3H), 6.77 (brs, 1H), 6.67–6.63 (m, 2H), 4.12 (t, *J* = 6.0 Hz, 2H), 3.62 (d, *J* = 16.8 Hz, 1H), 2.93 (s, 2H), 2.43 (s, 6H), 2.19 (d, *J* = 16.8 Hz,1H), 0.28 (brs, 2H), 0.12 (brs, 1H), −0.25 to −0.27 (m, 1H); ESI-MS: *m/z* = (M + H)^+^ 443.46.

### Synthesis of compound-20

#### Introduction

Synthesis of **compound-20** was achieved from synthesized intermediate **H-8**. **H-8** was reacted with **H-32** in a sealed tube to achieve **H-33**. **H-33** was treated with TiCl_4_ to afford **compound-20**. Supplementary Fig. 31 shows the synthetic scheme.

#### Preparation of H-33

To a stirred solution of **H-8** (0.30 g, 0.525 mmol, AMRI lot # IN-GUM-C-197), CuI (10.0 mg, 0.052 mmol), Cs_2_CO_3_ (0.340 g, 1.050 mmol), 1,10-phenathroline (20.0 mg, 0.105 mmol) and **H-32** (0.7 mL) was stirred at 130 °C for 24 h in a sealed tube. The progress of the reaction was monitored by TLC. Upon completion reaction, mixture was cooled to room temperature and diluted with EtOAc (150 mL), copper salts were filtered through celite pad, washed with excess of EtOAc (50.0 mL), filtrate was washed with sat. aq. NaHCO_3_ (3 × 50.0 ml), dried over Na_2_SO_4_, filtered and concentrated under reduced pressure to afford **H-33**[0.350 g,(crude), AMRI lot # IN-GUM-D-7] as a pale yellow oil.

ESI-MS: *m/z* = (M + H)^+^ 619.27.

#### Preparation of Compound-20

To a stirred solution of **H-33** (0.350 g, 0.566 mmol, AMRI lot # IN-GUM-D-7) in CH_2_Cl_2_ (30.0 mL) was charged with TiCl_4_ (5.0 mL) at 0 °C under argon atmosphere and the resulting reaction mixture was stirred at the same temperature for 6 h. The progress of the reaction was monitored by UPLC. Upon completion, the reaction was quenched by pouring in ice-cold sat. aq. NaHCO_3_ (150 mL), the resulted material was extracted with 10% MeOH in CH_2_Cl_2_ (3 × 150 mL). The combined organic layer was washed with sat. aq. NaHCO_3_ (2 × 50.0 mL), brine (2 × 50.0 mL), dried over Na_2_SO_4_, filtered, and concentrated under reduced pressure. The obtained pale yellow crude material was purified by preparative-HPLC (Method-A). The pure fractions were collected and CH_3_CN was concentrated under reduced pressure and the resulted material was lyophilized to afford compound-**20** [0.030 g, 13.22% (over two steps), AMRI lot # IN-GUM-D-9] as an off-white solid

^1^H NMR (400 MHz, DMSO-*d*_*6*_): δ 8.30 (s, 1H), 7.49–7.40 (s, 5H), 7.17 (d, *J* = 8.4 Hz, 2H), 6.87 (d, *J* = 8.4 Hz, 3H), 6.78 (brs, 1H), 6.67–6.63 (m, 2H), 4.03 (t, *J* = 6.0 Hz, 2H), 3.62 (d, *J* = 16.8 Hz, 1H), 2.94 (t, *J* = 4.8 Hz, 2H), 2.40 (s, 4H), 2.19 (d, *J* = 6.8 Hz, 1H), 0.27 (brs, 2H), 0.14–0.11 (m, 1H), −0.26 to −0.29 (m, 1H); ESI-MS: *m/z* = (M + H)^+^ 429.42.

### Synthesis of compound-21

#### Introduction

Synthesis of **compound-21** was achieved from synthesized intermediate **H-8**. **H-8** was reacted with **H-34** under Ullmann reaction conditions in a sealed tube to achieve **H-35**. **H-35** was treated for debenzylation with TiCl_4_ to afford **compound-21**. Supplementary Fig. 32 shows the synthetic scheme.

#### Preparation of H-35

To a stirred solution of **H-8** (0.200 g, 0.35 mmol, AMRI lot # IN-GUM-C-197), CuI (7.0 mg, 0.035 mmol), Cs_2_CO_3_ (0.227 g,0.70 mmol), 1,10-phenathroline (14.0 mg, 0.07 mmol) and **H-34** (0.4 mL) was stirred at 120 °C for 24 h in a sealed tube. The progress of the reaction was monitored by TLC. Upon completion reaction, mixture was cooled to room temperature and diluted with EtOAc (150 mL), copper salts were filtered through celite pad, washed with excess of EtOAc (50.0 mL), filtrate was washed with sat. aq. NaHCO_3_ (3 × 50.0 ml), dried over Na_2_SO_4_, filtered and concentrated under reduced pressure. The obtained crude was purified by flash chromatography by using silica gel (100–200 mesh) and eluted with 75–100% EtOAc in hexane. Combined pure column fractions were concentrated under reduced pressure to afford **H-35** (0.100 g, 50.00%, AMRI lot # IN-GUM-D-3) as a pale yellow oil. ESI-MS: *m/z* = (M + H)^+^ 533.26.

#### Preparation of compound-21

To a stirred solution of **H-35** (0.100 g, 0.187 mmol, AMRI lot # IN-GUM-D-3) in CH_2_Cl_2_ (10.0 mL) was charged with TiCl_4_ (1.0 mL) at 0 °C under argon atmosphere and the resulting reaction mixture was stirred at the same temperature for 5 h. The progress of the reaction was monitored by UPLC. Upon completion, reaction was quenched by pouring in ice-cold sat. aq. NaHCO_3_ (100 mL), the resulted material was extracted with 10% MeOH in CH_2_Cl_2_ (3 × 100 mL). The combined organic layer was washed with sat. aq. NaHCO_3_ (2 × 50.0 mL), brine (2 × 50.0 mL), dried over Na_2_SO_4_, filtered, and concentrated under reduced pressure. The obtained pale yellow crude material was purified by preparative-HPLC (Method-A). The pure fractions were collected and CH_3_CN was concentrated under reduced pressure. Aqueous layer (10.0 mL) was extracted with 10% MeOH in CH_2_Cl_2_ (3 × 15 mL); organic layer was washed with sat. aq. NaHCO_3_ (15.0 mL), dried over Na_2_SO_4_, filtered, and concentrated and the resulted material was lyophilized to afford **compound**-**21** (0.022 g, 26.50%, AMRI lot # IN-GUM-D-8) as an off-white solid.

^1^H NMR (400 MHz, DMSO-*d*_*6*_): δ 9.38 (s, 1H), 7.49–7.40 (s, 5H), 7.16 (d, *J* = 8.4 Hz, 2H), 6.89–6.84 (m, 3H), 6.77 (brs, 1H), 6.67–6.62 (m, 2H), 3.99 (t, *J* = 4.8 Hz, 2H), 3.62 (d, *J* = 16.8 Hz, 1H), 2.90 (t, *J* = 5.6 Hz, 2H), 2.63 (q, *J* = 6.8 Hz, 2H), 2.19 (d, *J* = 6.8 Hz, 1H), 1.31–1.23 (m, 1H), 1.03 (t, *J* = 7.2 Hz, 3H), 0.27 (brs, 2H), 0.15–0.12 (m, 1H), −0.25 to −0.28 (m, 1H); ESI-MS: *m/z* = (M + H)^+^ 443.21.

### Synthesis of compound-22

#### Introduction

Synthesis of **compound-22** was achieved from earlier synthesized intermediate **H-8**. **H-8** was reacted with **H-36** in a sealed tube to achieve **H-37**. **H-37** was treated for Boc deprotection using 2,2,2,-trifluoroethanol and TMSCl to get **H-38**. **H-38** upon reductive amination reaction with butyraldehyde to achieve **H-39**. **H-39** was treated with TiCl_4_ to afford **compound-22**. Supplementary Fig. 33 shows the synthetic scheme.

#### Preparation of H-37

To a stirred solution of **H-8** (0.500 g, 0.875 mmol, AMRI lot # IN-GUM-D-107) was charged with CuI (17.0 mg, 0.087 mmol), Cs_2_CO_3_ (0.570 g, 1.75 mmol), 1,10-phenathroline (34.0 mg, 0.175 mmol), **H-36** (0.704 g, 3.50 mmol) in butyronitrile (1.0 mL) and stirred at 130 °C for 24 h in a sealed tube. The progress of the reaction was monitored by TLC and UPLC-MS. Upon completion reaction, mixture was cooled to room temperature and diluted with EtOAc (150 mL), copper salts were filtered through celite pad, washed with excess of EtOAc (50.0 mL), filtrate was washed with saturated aqueous NaHCO_3_ (2 × 50.0 ml), dried over Na_2_SO_4_, filtered and concentrated under reduced pressure. The obtained crude material was purified by flash chromatography by using silica gel (100–200 mesh) and eluted with 35–40% EtOAc in hexanes. Combined pure fractions were concentrated under reduced pressure to afford **H-37** (0.320 g, 57.0%, AMRI lot # IN-GUM-D-132) as an off-white foam solid.

ESI-MS: *m/z* = (M + H)^+^ 645.47.

#### Preparation of H-38

To a stirred solution of **H-37** (0.320 g, 0.494 mmol, AMRI lot # IN-GUM-D-132) in 2, 2, 2-trifluoroethanol (5.0 mL) was charged with TMS-Cl (0.50 mL) at 0 °C. The resulting reaction mixture was stirred at room temperature for 3 h. The reaction mixture was concentrated under reduced pressure to afford **H-38** [0.350 g (crude HCl salt), AMRI lot # IN-GUM-D-134] as an off-white solid.

ESI-MS: *m/z* = (M + H)^+^ 545.15.

#### Preparation of H-39

To a stirred solution of **H-38** (0.350 g, 0.605 mmol, AMRI lot # IN-GUM-D-134) in MeOH (10.0 mL) was charged with butyraldehyde (0.4 mL, 3.027 mmol) followed by the addition of acetic acid (0.1 mL) at room temperature under argon atmosphere. The resultant reaction mixture was stir for 4 h. NaBH_3_CN (0.15 g, 2.420 mmol) was added to the reaction mixture in two to three portions, and stirring was continued for 24 h. The reaction mixture was diluted with CH_2_Cl_2_ (100 mL), CH_2_Cl_2_ layer washed with sat. aq. NaHCO_3_ (2 × 50.0 mL), brine (2 × 50.0 mL), dried over Na_2_SO_4_, filtered and concentrated under reduced pressure to afford **H-39** [0.370 g (crude), AMRI lot # IN-GUM-D-135] as pale yellow semi-solid.

ESI-MS: *m/z* = (M + H)^+^ 601.23.

#### Preparation of Compound-22

To a stirred solution of **H-39** (0.370 g, 0.616 mmol, AMRI lot # IN-GUM-D-135) in CH_2_Cl_2_ (10.0 mL) was charged with TiCl_4_ (4.00 mL) at 0 °C and the resulting reaction mixture was stirred at the same temperature for 3 h. The progress of the reaction was monitored by UPLC. Upon completion, reaction mixture was quenched by pouring in ice-cold NaHCO_3_ (100 mL), the resulted material was extracted with 10% MeOH in CH_2_Cl_2_ (3 × 100 mL). The combined organic layer was washed with sat. aq. NaHCO_3_ (3 × 50.0 mL), brine (2 × 30.0 mL), dried over Na_2_SO_4_, filtered, and concentrated under reduced pressure. The obtained crude material was purified by preparative-HPLC (Method-A). The pure fractions were collected and CH_3_CN was concentrated under reduced pressure. Aqueous layer (10.0 mL) was extracted with 10% MeOH in CH_2_Cl_2_ (3 × 20.0 mL), organic layer was washed with sat. aq. NaHCO_3_ (10.0 mL), dried over Na_2_SO_4_, filtered, and concentrated and the resulted material was lyophilized to afford **compound**-**22** [0.045 g, 15.10% (over three steps) AMRI lot # IN-GUM-D-138] as an off-white solid.

^1^H NMR (400 MHz, DMSO-*d*_*6*_): δ 9.41 (s, 1H), 7.49–7.40 (m, 5H), 7.15 (d, *J* = 8.4 Hz, 2H), 6.89–6.83 (m, 4H), 6.67–6.62 (m, 2H), 3.81 (d, *J* = 8.0 Hz, 2H), 3.62 (d, *J* = 17.2 Hz, 1H), 2.63–2.58 (m, 1H), 2.36–2.33 (m, 5H), 2.19 (d, *J* = 6.8 Hz, 1H), 1.90 (brs, 1H) 1.44–1.23 (m, 6H), 0.86 (t, *J* = 7.2 Hz, 3H), 0.27 (brs, 2H), 0.14–0.10 (m, 1H), −0.26 to −0.28 (m, 1H); ESI-MS: *m/z* = (M + H)^+^ 511.18.

### Synthesis of compound-23

#### Introduction

Synthesis of **compound-23** was achieved from earlier synthesized intermediate **H-38**. **H-38** was reacted with n-pentanol to achieve **H-40**. **H-40** was treated with 1.0 M BBr_3_ in CH_2_Cl_2_ to afford **compound-23**. Supplementary Fig. 34 shows the synthetic scheme.

#### Preparation of H-40

To a stirred solution of **H-38** (0.300 g, 0.519 mmol, AMRI lot # IN-GUM-D-124) in MeOH (10.0 mL) was charged with pentanal (1.00 mL, 2.076 mmol) followed by the addition of acetic acid (0.1 mL) at room temperature under argon atmosphere. The resultant reaction mixture was stir for 4 h. NaBH_3_CN (0.10 g, 1.557 mmol) was added to the reaction mixture in two to three lots, and stirring was continued for 16 h. Reaction mixture was diluted with CH_2_Cl_2_ (100 mL), CH_2_Cl_2_ layer washed with sat. aq. NaHCO_3_ (2 × 50.0 mL), brine (2 × 50.0 mL), dried over Na_2_SO_4_, filtered and concentrated under reduced pressure to afford **H-40** [0.350 g (crude), AMRI lot # IN-GUM-D-129] as pale yellow semi-solid.

ESI-MS: *m/z* = (M + H)^+^ 615.22.

#### Preparation of compound-23

To a stirred solution of **H-40** (0.350 g, 0.570 mmol, AMRI lot # IN-GUM-D-129) in CH_2_Cl_2_ (20.0 mL) was charged with TiCl_4_ (4.00 mL) at 0 °C and the resulting reaction mixture was stirred at the same temperature for 3 h. The progress of the reaction was monitored by UPLC. The reaction mixture was quenched by pouring in ice cold NaHCO_3_ (100 mL), the resulted material was extracted with 10% MeOH in CH_2_Cl_2_ (3 × 100 mL). The combined organic layer was washed with saturated aqueous NaHCO_3_ (3 × 50.0 mL), brine (2 × 30.0 mL), dried over Na_2_SO_4_, filtered, and concentrated under reduced pressure to obtain crude material. The obtained crude material was purified by preparative-HPLC (Method-A). The pure fractions were collected and CH_3_CN was concentrated under reduced pressure. Aqueous layer (10.0 mL) was extracted with 10% MeOH in CH_2_Cl_2_ (3 × 20.0 mL), organic layer was washed with sat. aq. NaHCO_3_ (10.0 mL), dried over Na_2_SO_4_, filtered, and concentrated and the resulted material was lyophilized to afford **compound**-**23** [0.025 g, 8.00% (over three steps) AMRI lot # IN-GUM-D-131] as an off-white solid.

^1^H NMR (400 MHz, DMSO-*d*_*6*_): δ 9.41 (s, 1H), 7.50–7.41 (m, 5H), 7.15 (d, *J* = 8.4 Hz, 2H), 6.92–6.83 (m, 4H), 6.77 (brs, 1H), 6.66–6.62 (m, 2H), 3.81 (d, *J* = 7.2 Hz, 2H), 3.62 (d, *J* = 16.8 Hz, 1H), 2.60–2.55 (m, 1H), 2.35–2.32 (m, 5H), 2.19 (d, *J* = 6.8 Hz, 1H), 1.95–1.88 (m, 1H), 1.41–1.36 (m, 4H), 1.26–1.23 (m, 4H), 0.84 (t, *J* = 6.4 Hz, 3H), 0.27 (brs, 2H), 0.14–0.10 (m, 1H), −0.26 to −0.28 (m, 1H); ESI-MS: *m/z* = (M + H)^+^ 525.16.

### Synthesis of compound-24

#### Introduction

Synthesis of **compound-24** was achieved from earlier synthesized intermediate **H-6**. **H-6** was reacted with **H-41** in a sealed tube to achieve **H-42**. **H-42** was treated with Pd/C to afford **H-43**. **H-43** was treated with 1.0 M BBr_3_ to achieve **H-44**. **H-45** was treated with propionaldehyde to afford **compound-24**. Supplementary Fig. 35 shows the synthetic scheme.

#### Preparation of H-42

To a stirred solution of **H-6** (1.00 g, 2.02 mmol, AMRI lot # IN-GUM-D-147) was charged with CuI (20 mg, 0.101 mmol), Pd(PPh_3_)_4_ (0.240 g, 0.20 mmol), **H-41** (0.50 g, 3.03 mmol) in TEA (20 mL) and stirred at 100 °C for 16 h in a sealed tube. The progress of the reaction was monitored by TLC and UPLC-MS. Upon completion reaction, mixture was cooled to room temperature and diluted with EtOAc (150 mL), copper salts were filtered through celite pad, washed with excess of EtOAc (25.0 mL), filtrate was concentrated under reduced pressure. The obtained crude material was purified by flash chromatography by using silica gel (100–200 mesh) and eluted with 20–40% EtOAc in hexanes. Combined pure fractions were concentrated under reduced pressure to afford **H-42** (0.750 g, 63.0%, AMRI lot # IN-GUM-D-170) as an off-white solid.

^1^H NMR (400 MHz, DMSO-*d*_*6*_): δ 7.50–7.40 (m, 5H), 7.33 (d, *J* = 8.4 Hz, 2H), 7.23 (d, *J* = 8.0 Hz, 2H), 7.05 (d, *J* = 8.4 Hz, 1H), 6.87–6.81 (m, 3H), 3.78 (s, 3H), 3.67 (d, *J* = 17.2 Hz, 1H), 3.60–3.56 (m, 1H), 3.41–3.36 (m, 1H), 3.25–3.19 (m, 3H), 2.28 (d, *J* = 16.8 Hz, 1H), 2.18–2.15 (m, 1H), 1.92–1.89 (m, 1H), 1.39 (s, 9H) 0.30–0.27 (m, 2H), 0.16 (brs, 1H), −0.32 to −0.35 (m, 1H).

#### Preparation of H-43

To a stirred solution of **H-42** (0.750 g, 1.334 mmol, AMRI lot # IN-GUM-D-170) in etanol (15.0 mL) was charged with 10% Pd/C (100 mg) under nitrogen. The resulting reaction mixture was stirred at room temperature for 72 h under hydrogen balloon atmosphere. Palladium catalyst was filtered through celite bed, washed the celite bed with excess ethanol. Filtrate was concentrated under reduced pressure to afford **H-43** [0.620 g (crude), AMRI lot # IN-GUM-D-172] as an off white solid; ESI-MS: *m/z* = (M + H)^+^ 567.57

#### Preparation of H-44

To a stirred solution of **H-43** [0.10 g, 0.176 mmol, AMRI lot # IN-GUM-D-172] in CH_2_Cl_2_ (10.0 mL) was charged with BBr_3_ (0.50 mL, 0.530 mmol, 1.0 M in CH_2_Cl_2_), at 0 °C under argon atmosphere. The reaction mixture was stirred for 3 h and quenched with MeOH (0.5 mL) at 0 °C. The reaction mixture was stir at room temperature for 1 h, upon reaction mixture was directly concentrated under reduced pressure to afford **H-44** [0.085 g (crude), AMRI lot # IN-GUM-D-176) as an off-white foam solid; ESI-MS: *m/z* = (M + H)^+^ 453.51.

#### Preparation of compound-24

To a stirred solution of **H-45** (0.080 g, 0.176 mmol, AMRI lot # IN-GUM-D-176) in MeOH (5.0 mL) was charged with propionaldehyde (0.10 mL, 0.707 mmol) followed by the addition of acetic acid (0.1 mL) at room temperature under argon atmosphere. The resultant reaction mixture was stir for 4 h. NaBH_3_CN (0.02 g, 0.352 mmol) was added to the reaction mixture in two to three lots, and stirring was continued for 16 h. Reaction mixture was diluted with CH_2_Cl_2_ (100 mL), CH_2_Cl_2_ layer washed with sat. aq. NaHCO_3_ (2 × 50.0 mL), brine (2 × 50.0 mL), dried over Na_2_SO_4_, filtered and concentrated under reduced pressure to obtained crude material. The crude was triturated with MTBE (3.0 mL) to get solid, filtered the solid product and washed with n-hexane and dried to afford **compound-24** (0.020 g, 21% AMRI lot # IN-GUM-D-177] as an off-white solid.

^1^H NMR (400 MHz, DMSO-*d*_*6*_): δ 9.40 (s, 1H), 7.49–7.40 (m, 5H), 7.15 (d, *J* = 8.4 Hz, 4H), 6.88 (d, *J* = 8.4 Hz, 1H), 6.80 (brs, 1H), 6.67–6.63 (m, 2H), 3.62 (d, *J* = 16.8 Hz, 1H), 3.10–2.65 (m, 6H), 2.32–2.03 (m, 4H), 1.64–1.53 (m, 6H), 0.87 (t, *J* = 6.4 Hz, 3H), 0.25 (brs, 2H), 0.14–0.12 (m, 1H), −0.30 to −0. 33 (m, 1H); ESI-MS: *m/z* = (M + H)^+^ 495.56.

### Synthesis of compound-25

#### Introduction

Synthesis of **compound-25** was achieved from synthesized intermediate **H-45**. **H-45** was reacted with Iodo fluro propane to afford **compound-25**. Supplementary Fig. 36 shows the synthetic scheme.

#### Preparation of compound-25

To a stirred solution of **H-45** [0.350 g, 0.774 mmol, AMRI lot # IN-GUM-D-181] in DMF (10.0 mL) was charged with DIPEA (0.20 mL, 1.548 mmol) followed by the addition of iodofluropropane (0.051 mL, 0.580 mmol) at 0 °C, under argon atmosphere. The reaction mixture was stirred for 24 h at below 16 °C. The reaction mixture was quenched with cold water and product was extracted with ethylacetate (3 × 50.0 mL). The combined organic layer was washed with water (2 × 50.0 mL) and brine (2 × 50.0 mL), dried over Na_2_SO_4_, filtered and concentrated under reduced pressure. The obtained crude material was purified by preparative-HPLC (Method-A). The pure fractions were collected and CH_3_CN was concentrated under reduced pressure. Aqueous layer (10.0 mL) was extracted with 10% MeOH in CH_2_Cl_2_ (3 × 20.0 mL); the organic layer was washed with sat. aq. NaHCO_3_ (10.0 mL), dried over Na_2_SO_4_, filtered, and concentrated and the resulted material was lyophilized to afford **compound**-**25** [0.045 g, 18.0% (over three steps) AMRI lot # IN-GUM-D-183] as an off-white solid.

^1^H NMR (400 MHz, DMSO-*d*_*6*_): δ 9.40 (s, 1H), 7.49–7.40 (m, 5H), 7.16 (d, *J* = 8.0 Hz, 2H), 7.10 d, *J* = 8.4 Hz, 2H), 6.89 (d, *J* = 8.0 Hz, 1H), 6.80 (brs, 1H), 6.66–6.63 (m, 2H), 4.51 (t, *J* = 6.0 Hz, 1H), 4.39 (t, *J* = 6.0 Hz, 1H),), 3.62 (d, *J* = 16.8 Hz, 1H), 2.68 –2.59 (m, 1H), 2.54–2.32 (m, 5H), 2.19 (d, *J* = 16.8 Hz, 1H), 2.02–1.91 (m, 2H), 1.89–1.72 (m, 3H), 1.60–1.57 (m, 2H), 1.36–1.33 (m, 1H), 1.18–1. 05 (m, 1H), 0.25 (brs, 2H), 0.14–0.11 (m, 1H), −0.31 to −0.33 (m, 1H); ESI-MS: *m/z* = (M + H)^+^ 525.16.

### Synthesis of compound-26

#### Introduction

Synthesis of **compound-26** was achieved from earlier synthesized intermediate **H-6**. **H-6** was reacted with **H-46** under Buchwald reaction conditions in a sealed tube to achieve **H-47**. **H-47** was subjected to demethylation using BBr_3_ to afford **compound-26**. Supplementary Fig. 37 shows the synthetic scheme.

#### Preparation of H-47

To a stirred solution of **H-6** (0.50 g, 1.010 mmol, AMRI lot # IN-GUM-D-147) was charged with Cs_2_CO_3_ (0.49 g, 1.515 mmol), **H-46** (0.17 g, 1.212 mmol) in 1,4-dioxane(20 mL) was degassed with argon for 5 min then charged with brett Phos (0.049 g, 0.101 mmol), brett Phos Pd-G3 (0.045 g, 0.050 mmol), The resultant reaction mixture was stirred at 90 °C for 16 h in a sealed tube. The progress of the reaction was monitored by TLC and UPLC-MS. Reaction mixture was cooled to room temperature and diluted with EtOAc (150 mL), Palladium salts wrer filtered through celite pad, washed with excess of EtOAc (25.0 mL), filtrate was concentrated under reduced pressure to obtain crude material. The obtained crude material was purified by flash chromatography by using silica gel (100–200 mesh) and eluted with 7–10% MeOH in CH_2_Cl_2_. Combined column fractions were concentrated under reduced pressure to afford **H-47** (0.120 g, 23.0%, AMRI lot # IN-GUM-D-175) as a yellow foam solid.

#### Preparation of compound-26

To a stirred solution of **H-47** [0.12 g, 0.235 mmol, AMRI lot # IN-GUM-D-175] in CH_2_Cl_2_ (10.0 mL) was charged with BBr_3_ (0.50 mL, 0.530 mmol, 1.0 M in CH_2_Cl_2_), at 0 °C under argon atmosphere. The reaction mixture was stirred for 3 h and quenched with MeOH (0.5 mL) at 0 °C. The reaction mixture was stir at room temperature for 1 h, upon reaction mixture was directly concentrated under reduced pressure to obtain crude material. The obtained crude material was purified by preparative-HPLC (Method-A). The pure fractions were collected and CH_3_CN was concentrated under reduced pressure. Aqueous layer (10.0 mL) was extracted with 10% MeOH in CH_2_Cl_2_ (3 × 20.0 mL); the organic layer was washed with sat. aq. NaHCO_3_ (10.0 mL), dried over Na_2_SO_4_, filtered, and concentrated and the resulted material was lyophilized to afford **compound**-**26** [0.048 g, 18.0% (over three steps) AMRI lot # IN-GUM-D-180] as an off-white solid.

^1^H NMR (400 MHz, DMSO-*d*_*6*_): δ 9.35 (s, 1H), 7.45–7.40 (m, 5H), 6.94 (d, *J* = 8.0 Hz, 2H), 6.85 d, *J* = 7.6 Hz, 2H), 6.64–6.59 (m, 3H), 6.44 (d, *J* = 8.4 Hz, 2H), 5.63 (t, *J* = 6.0 Hz, 1H), 3.59 (d, *J* = 16.8 Hz, 1H), 2.90 (t, *J* = 6.0 Hz, 2H), 2.39–2.32 (m, 5H), 2.16 (d, *J* = 17.2 Hz, 1H), 1.93–1.81 (m, 1H), 1.45–1.40 (m, 3H), 1.23 (s, 1H), 0.85 (t, *J* = 7.6 Hz, 3H), 0.26 (brs, 2H), 0.14–0.05 (m, 1H), −0.14 to −0.16 (m, 1H); ESI-MS: *m/z* = (M + H)^+^ 496.55.

### Synthesis of compound-27

#### Introduction

Synthesis of **Compound-27** was achieved from the commercially available **H-9** and earlier synthesized intermediate **H-8. H-9** was treated with MsCl in presence of triethylamine to get **H-10**. **H-10** was reacted with potassium thioacetate to afford **H-11**. **H-11** was treated with 30% NaOMe to afford **H-12**. Copper mediated thioarylation of **H-12** with **H-8** in presence of CuI and Cs_2_CO_3_ afforded **H-13**. Boc-deprotection of **H-13** using TMSCl in trifluoroethanol afforded **H-14**. The reductive amination of **H-14** with propionaldehyde **H-15** furnished **H-16**. Benzyl deprotection of **H-16** using TiCl_4_ in CH_2_Cl_2_ afforded **Compound-27**. Supplementary Fig. 38 shows the synthetic scheme.

#### Preparation of H-10

Diisopropylethylamine (DIPEA, 6.50 mL, 37.30 mmol) followed by Ms-Cl (2.00 mL, 24.87 mmol) were added to a stirred solution of **H-9** (5.00 g, 24.87 mmol) in CH_2_Cl_2_ (30.0 mL) at 0 °C under argon atmosphere. The reaction mixture was stirred for 1 h and diluted with CH_2_Cl_2_ (100 mL). The organic layer was washed with water (2 × 50.0 mL) and brine (2 × 50.0 mL) and dried over Na_2_SO_4_, filtered, and concentrated under reduced pressure to afford **H-10** [6.72 g (crude), AMRI lot # IN-GUM-D-173] as pale yellow oil.

^1^H NMR (400 MHz, CDCl_3_): δ 4.23–4.12 (m, 2H), 3.59–3.48 (m, 2H), 3.41–3.32 (m, 1H), 3.19–3.09 (m, 1H), 3.03 (s, 3H), 2.62 (brs, 1H), 2.09–1.99 (m, 1H), 1.81–1.65 (m, 1H), 1.46 (s, 9H).

#### Preparation of H-11

Potassium thioacetate (4.10 g, 36.02 mmol) was added to a stirred solution of **H-10** [6.70 g, 24.010 mmol, and AMRI lot #IN-GUM-D-173] in DMF (70.0 mL) under argon atmosphere. The reaction mixture was stirred for 16 h at 95 °C. Then, quenched with cold water (100 mL), extracted with MTBE (3 × 50.0 mL). The combined organic layer was washed with cold water (2 × 50.0 mL) and brine (2 × 50.0 mL), dried over Na_2_SO_4_, filtered, and concentrated under reduced pressure to afford **H-11** [3.60 g (crude), AMRI lot # IN-GUM-D-174] as brown oil.

^1^H NMR (400 MHz, DMSO-*d*_*6*_): δ 3.39–3.29 (m, 2H), 3.21–3.12 (m, 1H), 2.90–2.83 (m, 3H), 2.34 (s, 3H), 2.30–2.25 (m, 1H), 1.98–1.84 (m, 1H), 1.61–1.51 (m, 1H), 1.38 (s, 9H).

#### Preparation of H-12

Sodium methoxide solution (0.5 mL, 30% in methanol) was added to a stirred solution of **H-11** [0.50 g, 1.930 mmol, AMRI lot # IN-GUM-D-174] in MeOH (10.0 mL) at room temperature under argon atmosphere. The reaction mixture was stirred for 24 h at rt. Then, the reaction was quenched with cold water (10 mL), acidified with citric acid (pH ~ 5-6), extracted with ethyl acetate (3 × 50.0 mL). The combined organic layer was washed with cold water (2 × 50.0 mL) and brine (2 × 50.0 mL), dried over Na_2_SO_4_, filtered, and concentrated under reduced pressure to afford **H-12** [0.38 g (crude), AMRI lot # IN-GUM-D-184] as brown oil.

#### Preparation of H-13

The sealed tube was charged with **H-12** (0.10 g, 0.175 mmol, AMRI lot # IN-GUM-D-149), CuI (3.0 mg, 0.017 mmol), Cs_2_CO_3_ (0.113 g, 0.350 mmol), 1,10-phenanthroline (6.0 mg, 0.034 mmol), **H-8** (0.320 g, 0.698 mmol) and butyronitrile (1.0 mL) and the mixture was stirred at 130 °C for 24 h. The progress of the reaction was monitored by TLC and UPLC-MS. After completion of the reaction, solution was cooled to room temperature and diluted with EtOAc (30 mL); copper salts were filtered through celite pad, washed with excess of EtOAc (25 mL). The filtrate was concentrated under reduced pressure to obtain crude material which was purified by flash chromatography using silica gel (100–200 mesh) by eluting with 30–35% EtOAc in hexanes to afford **H-13** (0.150 g, 70.9%, AMRI lot # IN-GUM-E-63) as a pale yellow foam solid; MS (MM) m/z 661.54 [M + H]^+^.

#### Preparation of H-14

TMS-Cl (0.50 mL) was added to a stirred solution of **H-13** (0.150 g, 0.227 mmol, AMRI lot # IN-GUM-D-185) in trifluoroethanol (5.0 mL) at 0 °C. The resulting reaction mixture was stirred at room temperature for 4 h. The reaction mixture was concentrated under reduced pressure to afford **H-14** [0.120 g (crude HCl salt), AMRI lot # IN-GUM-D-187] as an off white solid; MS (MM) *m/z* 561.51 [M + H]^+^.

#### Preparation of H-16

Propionaldehyde **H-15** (0.50 mL, 0.857 mmol) followed by acetic acid (0.1 mL) were added to a stirred solution of **H-14** (0.120 g, 0.214 mmol, AMRI lot # IN-GUM-D-187) in MeOH (5.0 mL) at room temperature under argon atmosphere. Then, the reaction mixture was stirred for 4 h and added NaCNBH_3_ (0.10 g, 0.428 mmol) portion wise and stirring was continued for 24 h. Upon completion of reaction, the mixture was diluted with 10% MeOH/CH_2_Cl_2_ (50 mL). The organic layer was washed with sat. aq. NaHCO_3_ solution (2 × 20.0 mL), brine (2 × 20.0 mL), dried over Na_2_SO_4_, filtered and concentrated under reduced pressure to afford **H-16** [0.150 g (crude), AMRI lot # IN-GUM-D-188] as pale yellow semi-solid; MS (MM) m/z 603.58 [M + H]^+^.

#### Preparation of compound-27

TiCl_4_ (1.00 mL) was added to a stirred solution of **H-16** (0.150 g, 0.249 mmol, AMRI lot # IN-GUM-D-188) in CH_2_Cl_2_ (10.0 mL) at 0 °C. Then, the reaction was stirred at the same temperature for 4 h. The progress of the reaction was monitored by UPLC-MS, upon completion of the reaction; the solution was poured into saturated ice-cold NaHCO_3_ solution (100 mL). The resulted solution was extracted with 10% MeOH in CH_2_Cl_2_ (3 × 75 mL). The combined organic layer was washed with brine (2 × 25.0 mL), dried over Na_2_SO_4_, filtered and concentrated under reduced pressure to obtain crude material which was purified by preparative-HPLC (Method-A). The pure fractions were collected and CH_3_CN was concentrated under reduced pressure and the resulted aqueous phase (~10.0 mL) was extracted with 10% MeOH in CH_2_Cl_2_ (3 × 20.0 mL), organic layer was washed with sat. aq. NaHCO_3_ solution (10.0 mL), dried over Na_2_SO_4_, filtered, concentrated and dried under lyophilization to afford **compound-27** (0.009 g, 4.25%, AMRI lot # IN-GUM-E-191] as an off-white solid.

^1^H NMR (400 MHz, DMSO-*d*_*6*_): δ 9.40 (s, 1H), 7.49–7.42 (m, 5H), 7.26 (d, *J* = 8.4 Hz, 2H), 7.19 (d, *J* = 8.0 Hz, 2H), 6.90 (d, *J* = 8.0 Hz, 1H), 6.78 (brs, 1H), 6.68–6.63 (m, 2H), 3.62 (d, *J* = 17.2 Hz, 1H), 2.97 (d, *J* = 17.2 Hz, 2H), 2.76 (d, *J* = 7.2 Hz, 1H), 2.60–2.54 (m, 3H), 2.21(d, *J* = 17.2 Hz, 1H), 2.08–1.90 (m, 2H), 1.44–1.33 (m, 5H), 0.84 (t, *J* = 7.2 Hz, 3H), 0.26 (brs, 2H), 0.14–0.04 (m, 1H), −0.28 to −0.38 (m, 1H); MS (MM) m/z 513.51 [M + H]^+^.

### Synthesis of compound-28

#### Introduction

Synthesis of **compound-28** was achieved from earlier synthesized intermediate **H-6**. **H-6** was reacted with **H-17** in a sealed tube to achieve **H-18**. **H-18** treated with 1.0 M BBr_3_ to afford **compound-28**. Supplementary Fig. 39 shows the synthetic scheme.

#### Preparation of H-18

The seal tube was charged with **H-6** (0.10 g, 0.202 mmol, AMRI lot # IN-GUM-D-142), TMEDA (0.50 mL, 0.404 mmol), Pd(OAc)_2_ (5.0 mg, 0.020 mmol), tri(o-tolyl)phosphine (3.0 mg, 0.010 mmol), **H-17** (0.1 mL, 0.242 mmol) and DMF (3.0 mL) and then stirred at 100 °C for 24 h. Upon completion of the reaction (TLC and UPLC-MS), cooled to room temperature and diluted with EtOAc (50 mL). Palladium salts were filtered through celite pad, washed with excess of EtOAc (20.0 mL), filtrate was concentrated under reduced pressure to obtain crude material. The obtained crude material was purified by flash chromatography using silica gel (100–200 mesh) by eluting with 30–35% EtOAc in hexanes to afford **H-18** (0.075 g, 78%, AMRI lot # IN-GUM-D-143) as an off-white solid.

^1^H NMR (400 MHz, DMSO-*d*_*6*_): δ 7.62 (d, *J* = 8.4 Hz, 2H), 7.51 (d, *J* = 16.0 Hz, 1H), 7.49–7.45 (m, 5H), 7.28 (d, *J* = 8.0 Hz, 2H), 7.07 (d, *J* = 8.0 Hz, 1H), 6.87–6.82 (m, 3H), 6.49 (d, *J* = 16.0 Hz, 1H), 3.79 (s, 3H), 3.68 (brd, *J* = 16.8 Hz, 1H), 2.29 (brd, *J* = 16.8 Hz, 1H), 1.47 (s, 9H), 0.30–0.25 (m, 2H), 0.19–0.17 (m, 1H), -0.24 (d, *J* = 10.8 Hz, 1H); MS (MM) m/z 496.14 [M + H]^+^.

#### Preparation of compound-28

BBr_3_ (0.3 mL, 0.2828 mmol, 1.0 M in CH_2_Cl_2_) was added to a stirred solution of **H-18** (0.07 g, 0.141 mmol, AMRI lot # IN-GUM-D-143) in CH_2_Cl_2_ (5.0 mL) at 0 °C. Then, the reaction was warmed to room temperature and stirred for 4 h. The progress of the reaction was monitored by UPLC. The reaction was quenched with MeOH (0.5 mL) and then diluted with 10% MeOH in CH_2_Cl_2_ (75 mL). The organic layer was washed with saturated aqueous NaHCO_3_ solution (2 × 10.0 mL), brine (2 × 10.0 mL), dried over Na_2_SO_4_, filtered, and concentrated under reduced pressure to obtain crude material as a pale yellow solid. The obtained crude material was triturated with mixture of hexane and MTBE (50 ml) to get precipitate. Filtered the solid and dried to afford **compound-28** [0.045 g, 75%, AMRI lot # IN-GUM-D-145] as an off-white solid.

^1^H NMR (400 MHz, DMSO-*d*_*6*_): δ 12.40 (s, 1H), 9.46 (s, 1H), 7.62 (d, *J* = 8.0 Hz, 2H), 7.55 (d, *J* = 15.6 Hz, 1H), 7.48–7.43 (m, 5H), 7.29 (d, *J* = 8.0 Hz, 2H), 6.93 (d, *J* = 8.4 Hz, 1H), 6.85 (brs, 1H), 6.69–6.65 (m, 2H), 6.50 (d, *J* = 16.0 Hz, 1H), 3.68 (brd, *J* = 16.8 Hz, 1H), 2.22 (brd, *J* = 17.2 Hz, 1H), 0.30–0.22 (m, 2H), 0.19–0.17 (m, 1H), -0.32 (brd, *J* = 10.8 Hz, 1H); MS (MM) m/z 426.07 [M + H]^+^.

### Synthesis of compound-29

#### Introduction

Synthesis of **compound-29** was achieved from earlier synthesized intermediate **H-6**. **H-6** was reacted with **H-11** in a sealed tube to achieve **H-12**. **H-12** was treated with 1.0 M BBr_3_ in CH_2_Cl_2_ to get **H-13. H-13** was treated with **1-iodofluoropropane** to afford **compound-29**. Supplementary Fig. 40 shows the synthetic scheme.

#### Preparation of H-20

The seal tube was charged with **H-6** (1.750 g, 3.535 mmol, AMRI lot # IN-GUM-D-167), CuI (0.070 g, 0.353 mmol), Cs_2_CO_3_ (2.30 g, 7.070 mmol), 1,10-phenanthroline (0.130 g, 0.707 mmol), **H-19** (3.00 g, 14.141 mmol) and butyronitrile (4.0 mL) and then stirred at 130 °C for 24 h. The progress of the reaction was monitored by TLC and UPLC-MS. Upon completion of reaction, the mixture was cooled to room temperature and diluted with EtOAc (150 mL), copper salts were filterd through celite pad, washed with excess of EtOAc (100.0 mL), filtrate was concentrated under reduced pressure to obtain crude material. The obtained crude material was purified by flash chromatography by using silica gel (100–200 mesh) by eluting with 30–35% EtOAc in hexanes to afford **H-20** (1.51 g, 73%, AMRI lot # IN-GUM-D-190) as a pale yellow gel.

^1^H NMR (400 MHz, DMSO-*d*_*6*_): δ 7.51–7.40 (m, 5H), 7.16 (d, *J* = 8.4 Hz, 2H), 7.00 (d, *J* = 8.8 Hz, 1H), 6.87–6.80 (m, 5H), 3.96–3.86 (m, 2H), 3.78 (s, 3H), 3.67 (brd, *J* = 17.2 Hz, 1H), 3.47–3.40 (m, 1H), 3.38–3.32 (m, 1H), 3.26–3.21 (m, 1H), 3.08–3.04 (m, 1H), 2.59–2.55 (m, 1H), 2.27 (brd, *J* = 17.4 Hz, 1H), 2.08–1.90 (m, 1H), 1.73–1.64 (m, 1H), 1.38 (s, 9H), 0.29–0.26 (m, 2H), 0.14 (brs, 1H), −0.23 (d, *J* = 10.4 Hz, 1H); MS (MM) m/z 569.25 [M + H]^+^.

#### Preparation of H-21

Borontribromide (10.00 mL, 11.619 mmol, 1.0 M in CH_2_Cl_2_) was added to a stirred solution of **H-20** (2.20 g, 3.873 mmol, AMRI lot # IN-GUM-D-190) in CH_2_Cl_2_ (30.0 mL) at 0 °C. Then the reaction was warmed to room temperature and stirred for 8 h. The progress of the reaction was monitored by UPLC. The reaction was quenched with MeOH (3.0 mL) and then diluted with 10% MeOH in CH_2_Cl_2_ (150 mL). The organic layer was washed with saturated aqueous NaHCO_3_ (2 × 50.0 mL), brine (2 × 50.0 mL), dried over Na_2_SO_4_, filtered, and concentrated under reduced pressure to obtain crude material as a pale yellow solid. The obtained crude material was triturated with mixture of hexane and MTBE (50 ml) to get precipitate. Filtered the solid and dried to afford **H-21** [1.50 g, 81%, AMRI lot # IN-GUM-D-194] as an off-white solid.

^1^H NMR (400 MHz, DMSO-*d*_*6*_): δ 7.51–7.39 (m, 5H), 7.17 (d, *J* = 8.4 Hz, 2H), 6.96–6.72 (m, 4H), 6.67–6.63 (m, 2H), 3.92–3.80 (m, 2H), 3.62 (brd, *J* = 17.2 Hz, 1H), 3.07–2.72 (m, 4H), 2.19 (brd, *J* = 17.4 Hz, 1H), 1.95–1.86 (m, 1H), 1.54–1.43 (m, 2H), 0.29–0.26 (m, 2H), 0.11 (brs, 1H), −0.27 (d, *J* = 10.4 Hz, 1H); MS (MM) m/z 569.25 [M + H]^+^.

#### Preparation of compound-29

To a stirred solution of **H-21** [1.50 g, 3.303 mmol, AMRI lot # IN-GUM-D-194] in DMF (20.0 mL) was added DIPEA (1.130 mL, 6.606 mmol) followed by **iodofluoropropane** (0.40 mL, 3.303 mmol) at 0 °C, under argon atmosphere. The reaction mixture was stirred for 48 h at below 20 °C. Then, quenched the reaction with cold water to get solid, filtered the solid and washed with excess water and dried to obtain brown crude solid which was purified by flash chromatography using silicagel (230-400 mesh) by eluting with 3-7% MeOH/CH_2_Cl_2_ to afford **Compound-29** [0.850 g, 56.00%, AMRI lot # IN-GUM-D-197] as an off-white solid.

^1^H NMR (400 MHz, DMSO-*d*_*6*_): δ 9.37 (s, 1H), 7.51–7.39 (m, 5H), 7.16 (d, *J* = 8.0 Hz, 2H), 6.88–6.76 (m, 4H), 6.67–6.62 (m, 2H), 4.52 (t, *J* = 5.6 Hz, 1H), 4.40 (t, *J* = 6.0 Hz, 1H), 3.82 (d, *J* = 6.4 Hz, 2H), 3.62 (d, *J* = 16.8 Hz, 1H), 2.63–2.58 (m, 3H), 2.46–2.34 (m, 4H), 2.19 (d, *J* = 17.2 Hz, 1H), 1.94–1.74 (m, 3H), 1.50–1.45 (m, 1H), 0.27 (brs, 2H), 0.14–0.11 (m, 1H), −0.24 to −0.27 (m, 1H); MS (MM) m/z 515.65 [M + H]^+^.

### Synthesis of compound-4A (30) and compound 4B (31)

#### Introduction

Synthesis of **compound-30** and **compound-31** was achieved from earlier synthesized intermediate **H-8**. Copper mediated O-arylation of **H-8** with **H-22** in presence of CuI and Cs_2_CO_3_ afforded **H-23**. **H-23** was treated with TMS-Cl in trifluoroethanol to afford **H-24**. **H-24** was treated with **H-22** to achieve **H-25**. **H-25** was reacted with TiCl_4_ to afford **compound-30** and **compound-31**. Supplementary Fig. 41 shows the synthetic scheme.

#### Preparation of H-23

The seal tube was charged with **H-8** (1.00 g, 1.751 mmol, AMRI lot # IN-GUM-D-149), CuI (33.0 mg, 0.175 mmol), Cs_2_CO_3_ (1.13 g, 3.502 mmol), 1,10-phenanthroline (63.0 mg, 0.20 mmol), **H-22** (1.30 g, 7.105 mmol) and butyronitrile (2.0 mL) and then stirred at 130 °C for 24 h. The progress of the reaction was monitored by TLC and UPLC-MS. Upon completion of reaction, the mixture was cooled to room temperature and diluted with EtOAc (250 mL). Copper salts were filterd through celite pad, washed with excess of EtOAc (100.0 mL), the filtrate was concentrated under reduced pressure to obtain crude material. The obtained crude material was purified by flash chromatography using silica gel (230–400 mesh) by eluting with 30–35% EtOAc in hexanes to afford **H-23** (0.880 g, 74%, AMRI lot # IN-GUM-D-159) as a pale yellow foam solid.

^1^H NMR (400 MHz, DMSO): δ 7.49–7.40 (m, 9H), 7.37–7.35 (m, 1H), 7.17 (d, *J* = 8.4 Hz, 2H), 7.02 (d, *J* = 8.4 Hz, 1H), 6.93–6.86 (m, 5H), 5.12 (s, 2H), 4.07 (d, *J* = 6.8 Hz, 2H), 3.94 (brs, 2H), 3.68 (d, *J* = 16.8 Hz, 3H), 2.95–2.90 (m, 1H), 2.27 (brd, *J* = 17.2 Hz, 1H), 1.37 (s, 9H), 0.30 (brs, 2H), 0.14 (brs, 1H), -0.23 (d, *J* = 10. Hz, 1H); MS (MM) m/z 631.44 [M + H]^+^.

#### Preparation of H-24

TMSCl (1.50 mL) was added to a stirred solution of **H-23** (0.820 g, 1.396 mmol, AMRI lot # IN-GUM-D-159) in trifluoroethanol (10.0 mL) at 0 °C. The resulting mixture was stirred at room temperature for 3 h and concentrated under reduced pressure to afford **H-24** [0.810 g (crude HCl salt), AMRI lot # IN-GUM-D-160] as an off white solid; MS (MM) m/z 531.12 [M + H]+.

#### Preparation of H-25

To a stirred solution of **H-24** [0.80 g, 1.415 mmol, AMRI lot # IN-GUM-D-160] in DMF (20.0 mL) was added DIPEA (0.50 mL, 2.83 mmol) followed by **iodofluoropropane** (0.15 mL, 1.415 mmol) at 0 °C under argon atmosphere. The reaction mixture was stirred for 24 h at below 20 °C. The reaction mixture was quenched with cold water to get solid, filtered the solid, washed with excess water, and dried to obtain brown crude solid. Obtained crude was purified by flash chromatography using silicagel (230-400 mesh) by eluting with 5-8% MeOH/CH_2_Cl_2_ to afford **H-25** [0.37 g, 44.00%, AMRI lot # IN-GUM-D-163] as an off-white solid; MS (MM) m/z 591.20 [M + H]^+^.

#### Preparation of compound-30 and compound-31

TiCl_4_ (3.00 mL) was added to a stirred solution of **H-25** (0.370 g, 0.627 mmol, AMRI lot # IN-GUM-D-163) in CH_2_Cl_2_ (30.0 mL) at 0 °C and the resulting mixture was stirred at the same temperature for 4 h. The progress of the reaction was monitored by UPLC. The reaction mixture was quenched by pouring into ice-cold saturated NaHCO_3_ solution (150 mL); the resulted material was extracted with 10% MeOH in CH_2_Cl_2_ (3 × 150 mL). The combined organic layers were washed with saturated NaHCO_3_ solution (3 × 50.0 mL), brine (3 × 50.0 mL), dried over Na_2_SO_4_, filtered, and concentrated under reduced pressure to obtain crude material. The obtained crude material was subjected to preparative-HPLC (Method-A) purification and isolated two compounds. The pure fractions were collected and CH_3_CN was concentrated under reduced pressure. Aqueous layer (10.0 mL) was extracted with 10% MeOH in CH_2_Cl_2_ (3 × 20.0 mL); combined organic layers were washed with saturated aq. NaHCO_3_ solution (10.0 mL), dried over Na_2_SO_4_, filtered, and concentrated. The obtained materials were dried under lyophilization to afford **Compound**-**30** [0.040 g, 12.77%, AMRI lot # IN-GUM-D-168-3] as an off-white solid and **Compound**-**31** [0.040 g, 12.38%, AMRI lot # IN-GUM-D-168-4] as an off-white solid.

**Compound**-**30:** ^1^H NMR (400 MHz, DMSO-*d*_*6*_): δ 9.41 (s, 1H), 7.51–7.40 (m, 5H), 7.16 (d, *J* = 8.4 Hz, 2H), 6.89–6.77 (m, 4H), 6.67–6.62 (m, 2H), 4.49 (t, *J* = 6.0 Hz, 1H), 4.37 (t, *J* = 6.0 Hz, 1H), 4.05 (d, *J* = 6.8 Hz, 2H), 3.62 (d, *J* = 16.8 Hz, 1H), 3.28 (t, *J* = 6.4 Hz, 2H), 2.99–2.83 (m, 2H), 2.78–2.72 (m, 1H), 2.48–2.38 (m, 2H), 2.19 (d, *J* = 16.8 Hz, 1H), 1.67–1.57 (m, 2H), 0.27–0.21 (m, 2H), 0.14–0.12 (m, 1H), −0.27 (brd, *J* = 10.4 Hz, 1H); MS (MM) m/z 501.18 [M + H]^+^.

**Compound**-**31**: ^1^H NMR (400 MHz, DMSO-*d*_*6*_): δ 9.41 (s, 1H), 7.51–7.40 (m, 5H), 7.16 (d, *J* = 8.4 Hz, 2H), 6.99–6.77 (m, 4H), 6.67–6.62 (m, 2H), 4.05 (d, *J* = 6.8 Hz, 2H), 3.64–3.59 (m, 3H), 3.31–3.20 (m, 4H), 2.99–2.90 (m, 2H), 2.78–2.72 (m, 1H), 2.19 (d, *J* = 16.8 Hz, 1H), 1.73–1.66 (m, 2H), 0.27–0.21 (m, 2H), 0.14–0.12 (m, 1H), −0.27 (brd, *J* = 10.4 Hz, 1H); MS (MM) m/z 517.18 [M + H]^+^.

### Reporting summary

Further information on research design is available in the Nature Research Reporting Summary linked to this article.

## Supplementary information

Supplementary Information

Reporting Summary

## Data Availability

All x-ray structures have been deposited in the protein databank (PDB) under accession IDs 8DU6, 8DU8, 8DU9, 8DUB, 8DUC, 8DUD, 8DUG, 8DUG, 8DUI, 8DUK, 8DUS, 8DV5, 8DV7, 8DV8, 8DVB, 7TE7. Structures can be found at www.RCSB.org. The RNAseq data that support the findings of this study are have been registered with the BioProject database and are openly available at the National Center for Biotechnology Information (NCBI) Sequence Read Archive (SRA) repository at https://www.ncbi.nlm.nih.gov/sra/PRJNA889442, reference number PRJNA889442^50^. All other data that supports this work is available from the authors upon request.
