# Supplementary material for: Unconventional isoquinoline-based SERMs elicit fulvestrant-like transcriptional programs in ER+ breast cancer cells
Source: NPJ Breast Cancer. 2022 Dec 14;8:130. doi: 10.1038/s41523-022-00497-9 (PMC9748900; doi:10.1038/s41523-022-00497-9)
Supplement: Supplementary file 1 — Supplementary Information [file 41523_2022_497_MOESM1_ESM.pdf]

SUPPLEMENTARY FIGURES

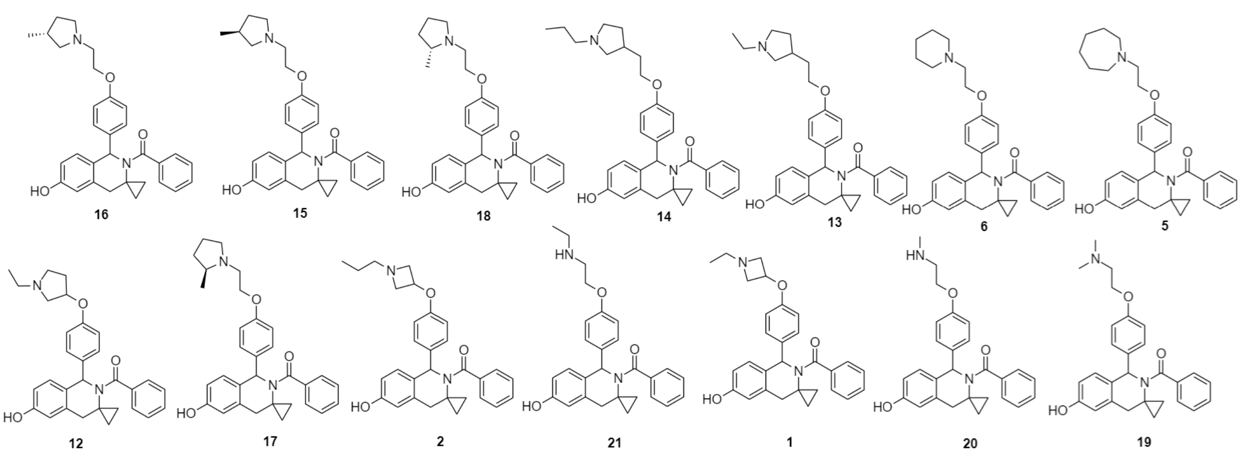

Supplementary Figure 1: Chemical structures of the first 14 T6I derivatives.

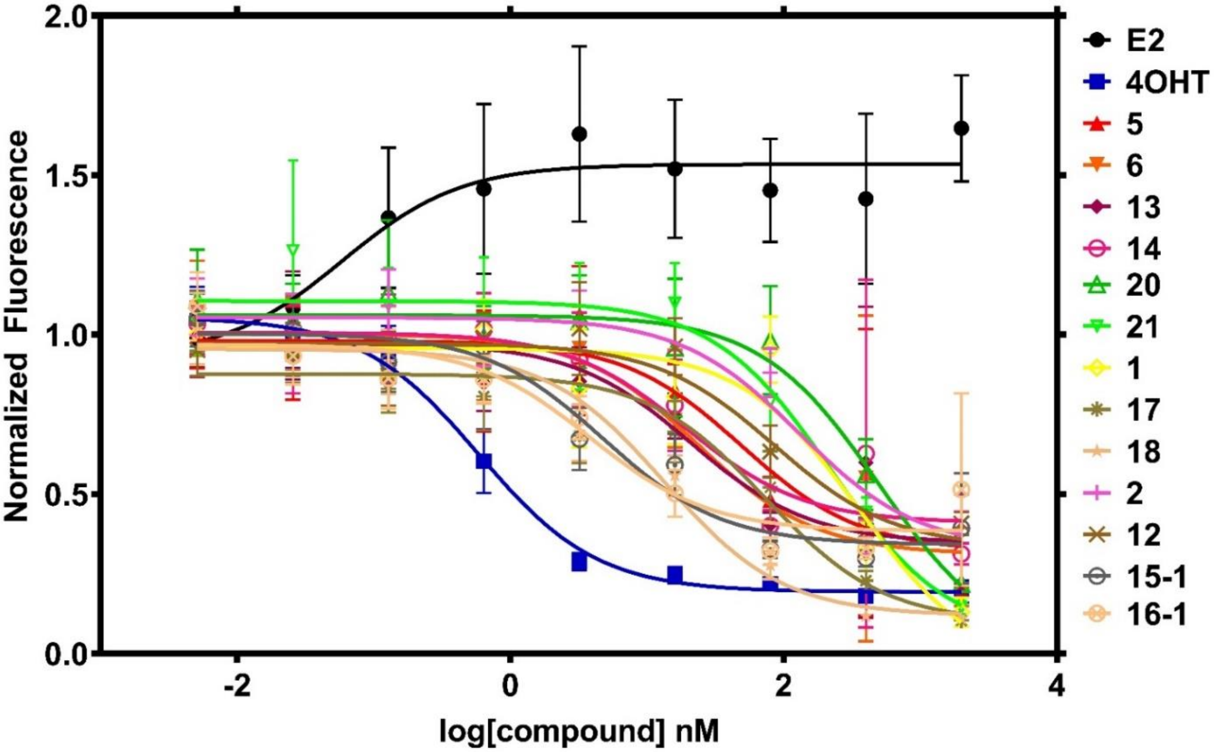

Supplementary Figure 2: Estrogen response element reporter gene expression in the presence of 1 nM E2. X-1 indicates molecules that have been chirally purified and the first peak tested. Second peak was inactive. Data shown are the mean of three replicates + std. dev..

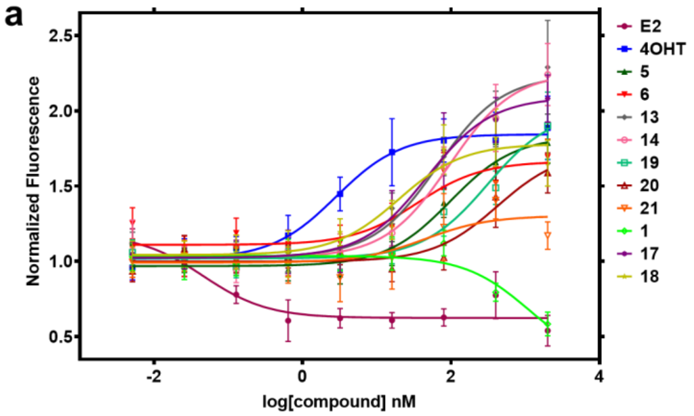

b

Ligand	IC_{50} (nM)	R^2	Max
E2	0.05 ± 0.02	0.76	0.62 ± 0.02
4OHT	2.83 ± 0.06	0.89	1.84 ± 0.04
5	97.96 ± 0.06	0.93	1.83 ± 0.06
6	34.98 ± 0.07	0.73	1.67 ± 0.07
13	65.93 ± 0.09	0.91	2.23 ± 0.08
14	95.15 ± 0.08	0.92	2.26 ± 0.08
19	310.7 ± 0.11	0.88	2.00 ± 0.11
20	423.8 ± 0.13	0.78	1.73 ± 0.13
21	ND	ND	1.04 ± 0.08
1	1,127 ± 0.72	0.72	0.32 ± 0.02
17	47.17 ± 0.07	0.91	2.09 ± 0.07
18	19.61 ± 0.07	0.84	1.78 ± 0.06

Supplementary Figure 3: Influence of representative T6Is on halo-ERα in T47D breast cancer cells. A) Dose response curves for halo-ERα accumulation after 24 hours treatment with E2, 4OHT, and representative T6I. B) IC_{50}, Goodness of Fit (R^2), and normalized signal at maximum dose (1 μM). All data are the mean of three treatments ± std. dev. and are normalized to cell count in their respective wells.

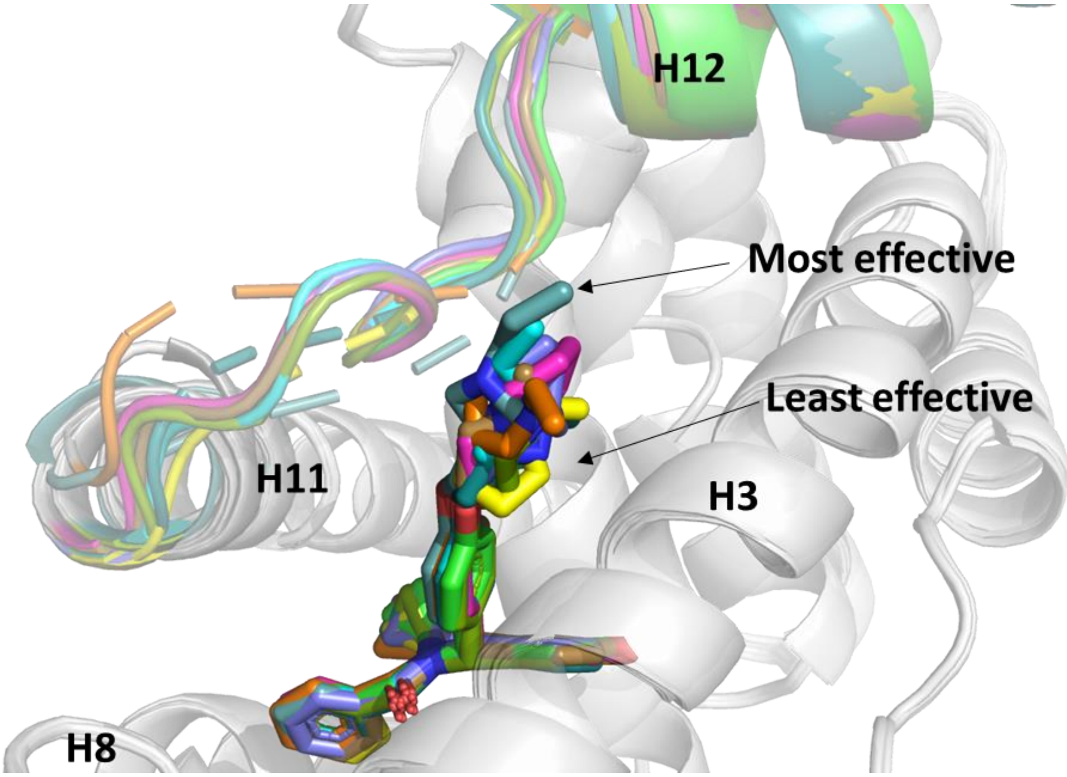

Supplementary Figure 4: Superposition of first 10 x-ray co-crystal structures of T6I SERMs in complex with ERα LBD highlighting the relationship between side-arm position relative to H12 and anti-transcriptional potency.

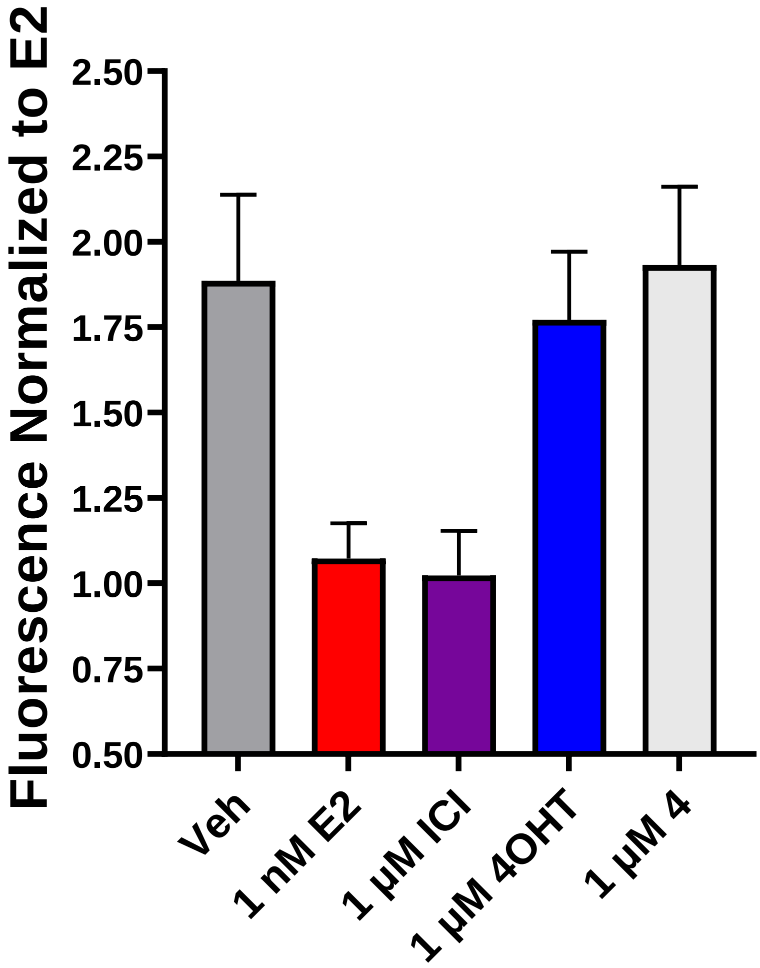

Supplementary Figure 5: In-cell western analysis of T6I-4 compared to E2, ICI, and 4OHT in T47D breast cancer cells. Data are the mean of three replicates ± std. dev.

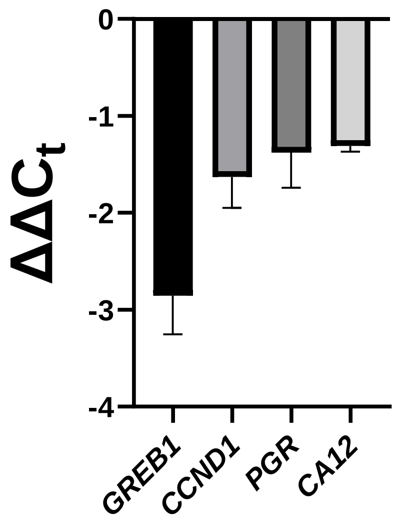

Supplementary Figure 6: Induction of ERα target gene expression. $\Delta\Delta C_t = \Delta C_t(E2) - \Delta C_t(veh)$.

Data are the mean of three replicates ± std. dev..

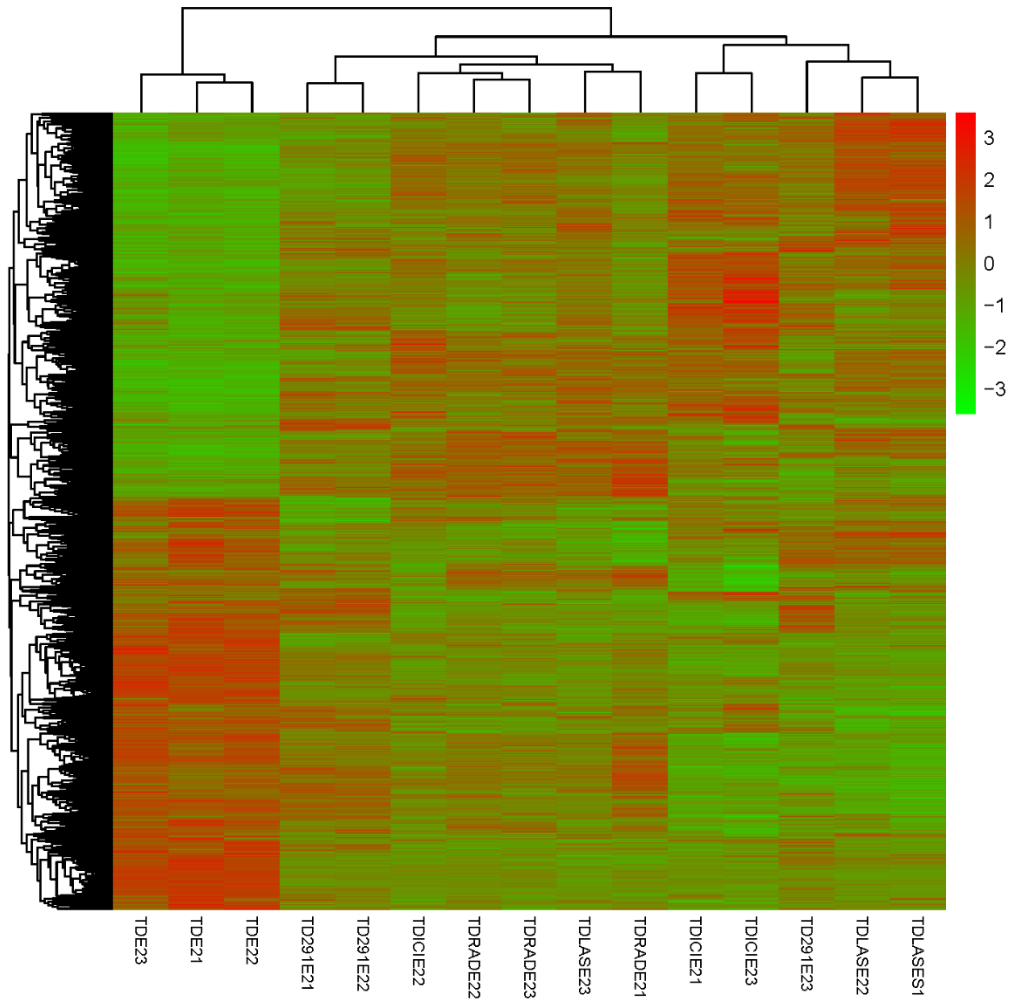

Supplementary Figure 7: Heatmap of differentially expressed genes in T47D cells treated with vehicle, 1 nM E2, or 29P1A, laso, RAD, ICI in the presence of 1 nM E2. TDE2 are replicates of T47D cells treated with E2. TD-291E2 are replicates of T47D cells treated with E2 and T6I-29-1A. TDLASE2 are replicates of T47Ds treated with E2 and laso. TDICIE2 are replicates of T47Ds treated with E2 and ICI. TDRADE2 are replicates of T47Ds treated with RAD1901 and E2.

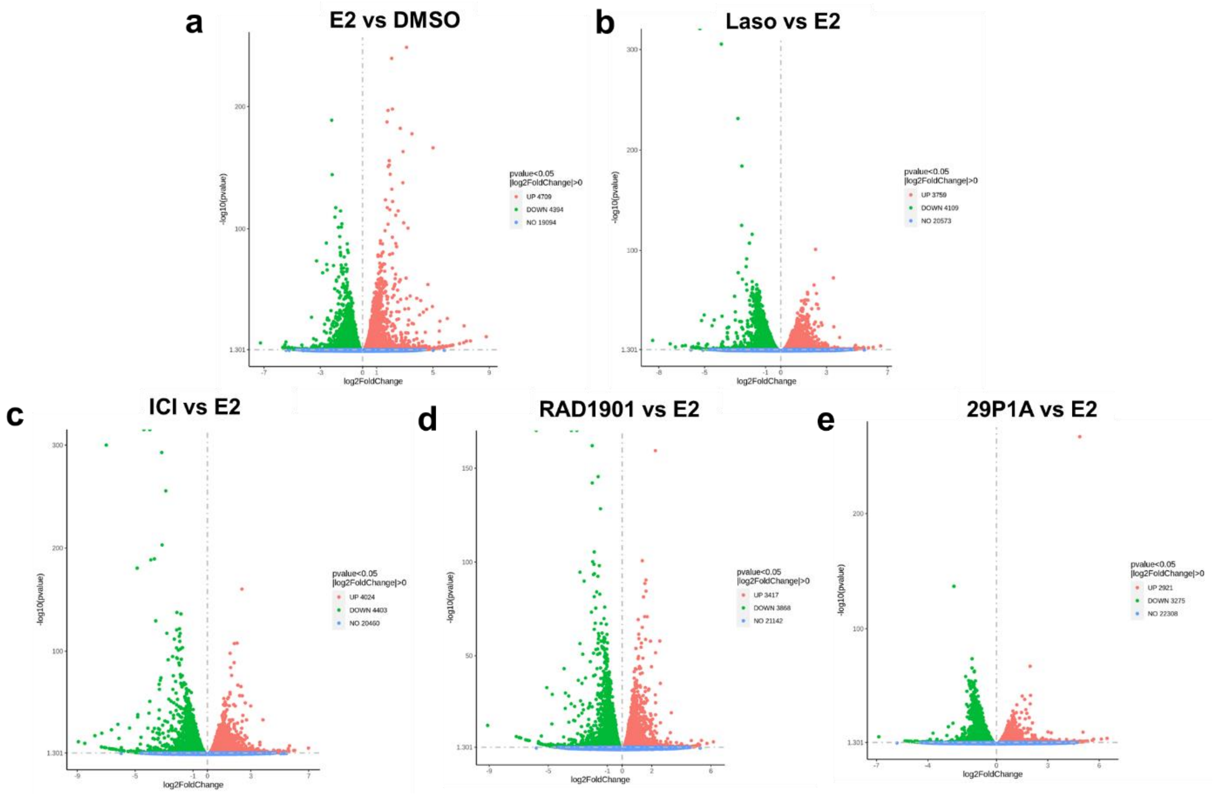

Supplementary Figure 8: Volcano plots of differentially expressed genes between A) E2 and vehicle (DMSO), B) Laso, C) ICI, and D) 29P1A. All antagonists are in the presence of 1 nM E2 and compared to 1 nM E2-only treatment.

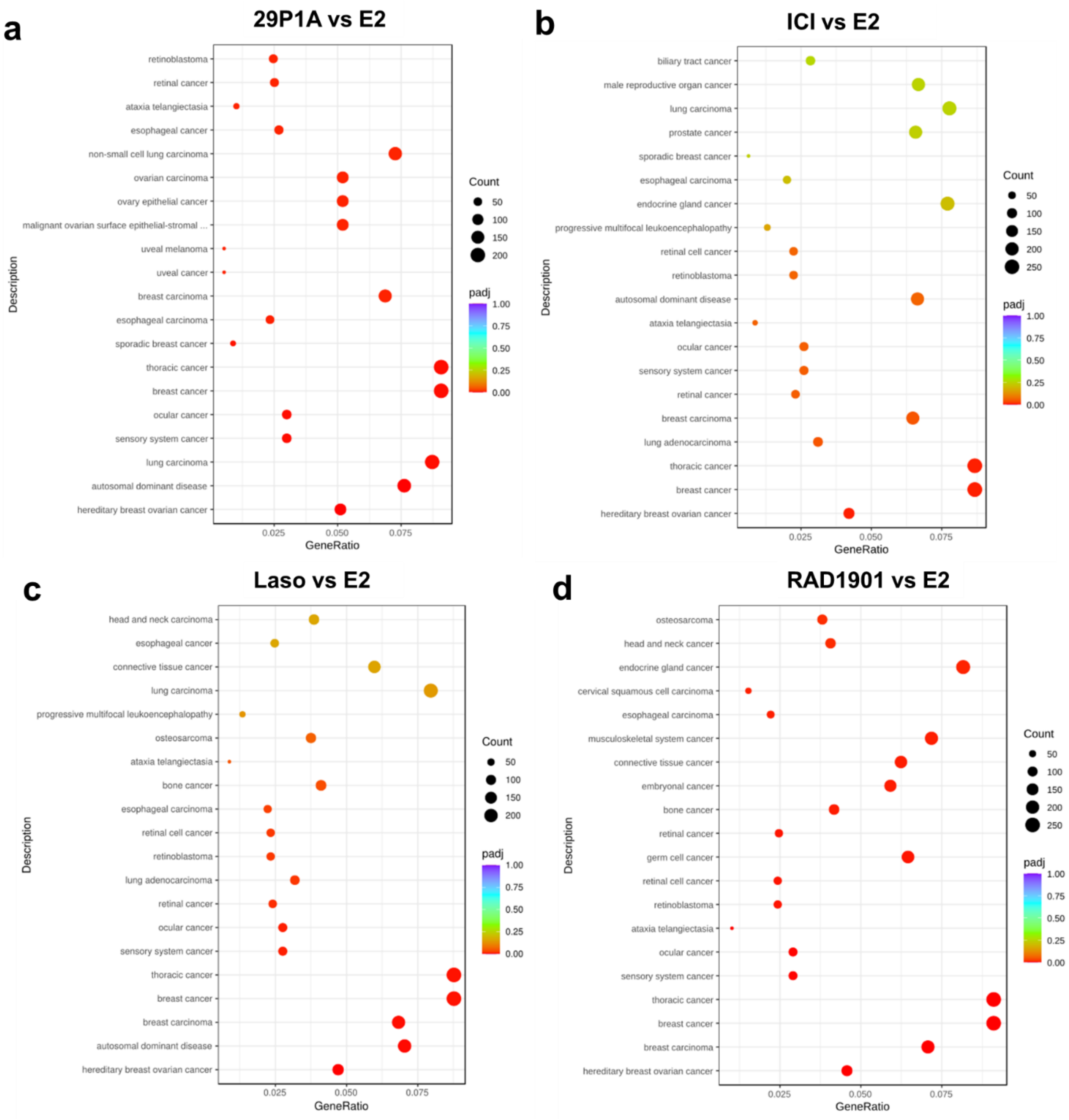

Supplementary Figure 9: Disease ontology (DO) enrichment analysis for T47D cells in in the presence of 1 nM E2 and treated with A) T6I-29P1A, B) ICI, C) Laso, and D) RAD1901. DO terms with corrected *P*-value less than 0.05 were included as significantly enriched by differential expressed genes compared to E2 only treated cells.

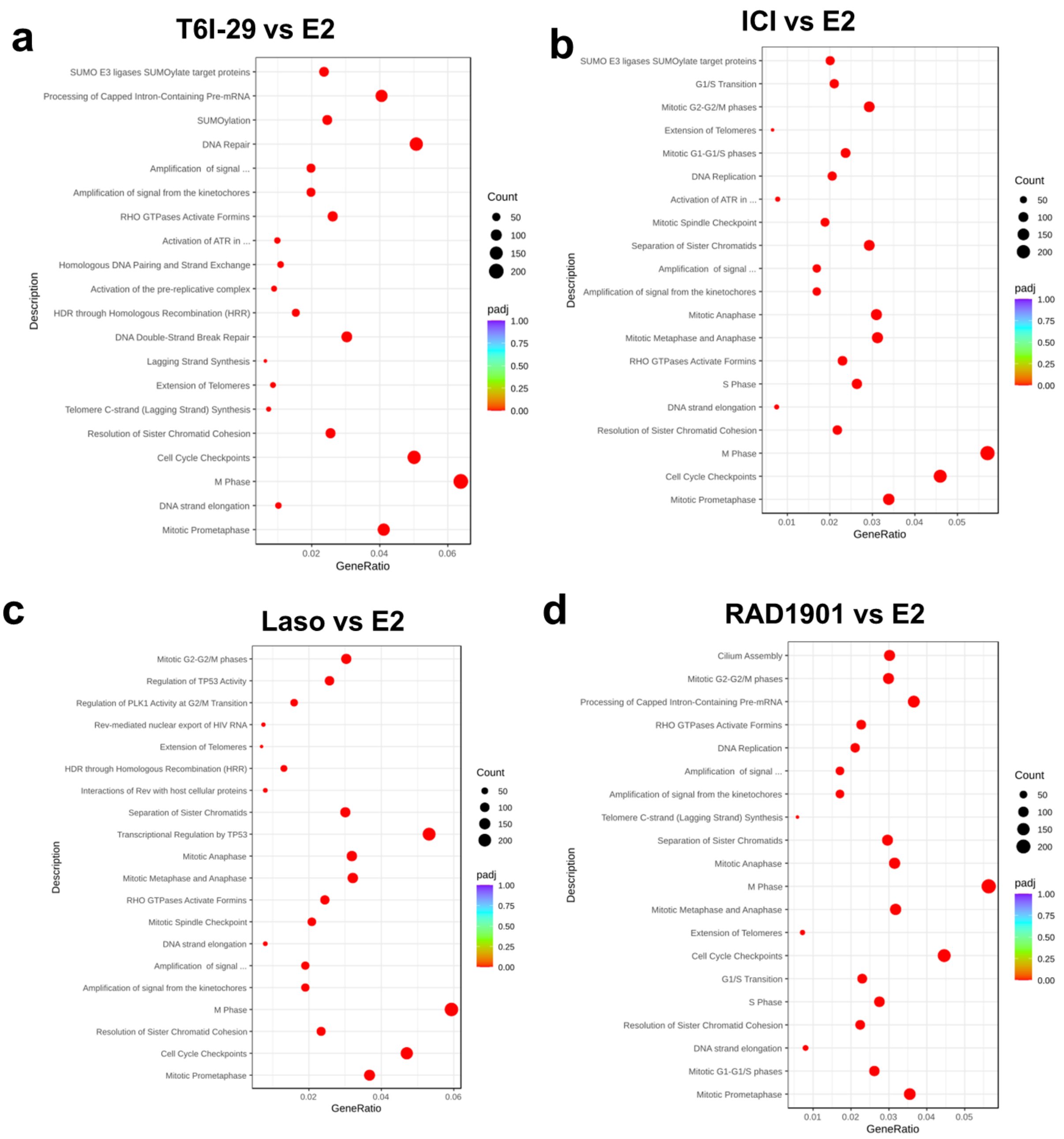

Supplementary Figure 10: Reactome pathway analysis for T47D cells in in the presence of 1 nM E2 and treated with A) T6I-29P1A, B) ICI, C) Laso, and D) RAD1901. GO terms with corrected *P*-value less than 0.05 were included as significantly enriched by differential expressed genes compared to E2 only treated cells.

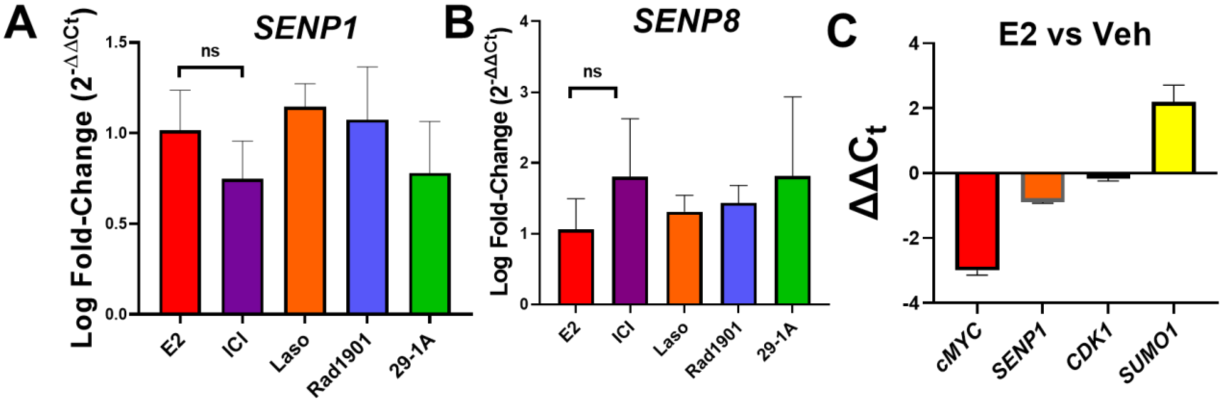

Supplementary Figure 11: Induction of SUMO-related genes in T47D breast cancer cells. A) Effect of 1 µM antagonist treatment on *SENP1* expression in the presence of 1 nM E2, normalized to 1 nM E2 alone. B) Effect of 1 µM antagonist treatment on *SENP8* expression in the presence of 1 nM E2, normalized to 1 nM E2 alone. C) Change in *cMYC, SENP1, CDK1, SUMO1* gene expression in T47D breast cancer cells treated with 1 nM E2 compared to vehicle (veh). $\Delta\Delta C_t = \Delta C_t(E2) - \Delta C_t(veh)$. Data are the mean of three replicates ± std. dev. and significance was determined by unpaired t-test.

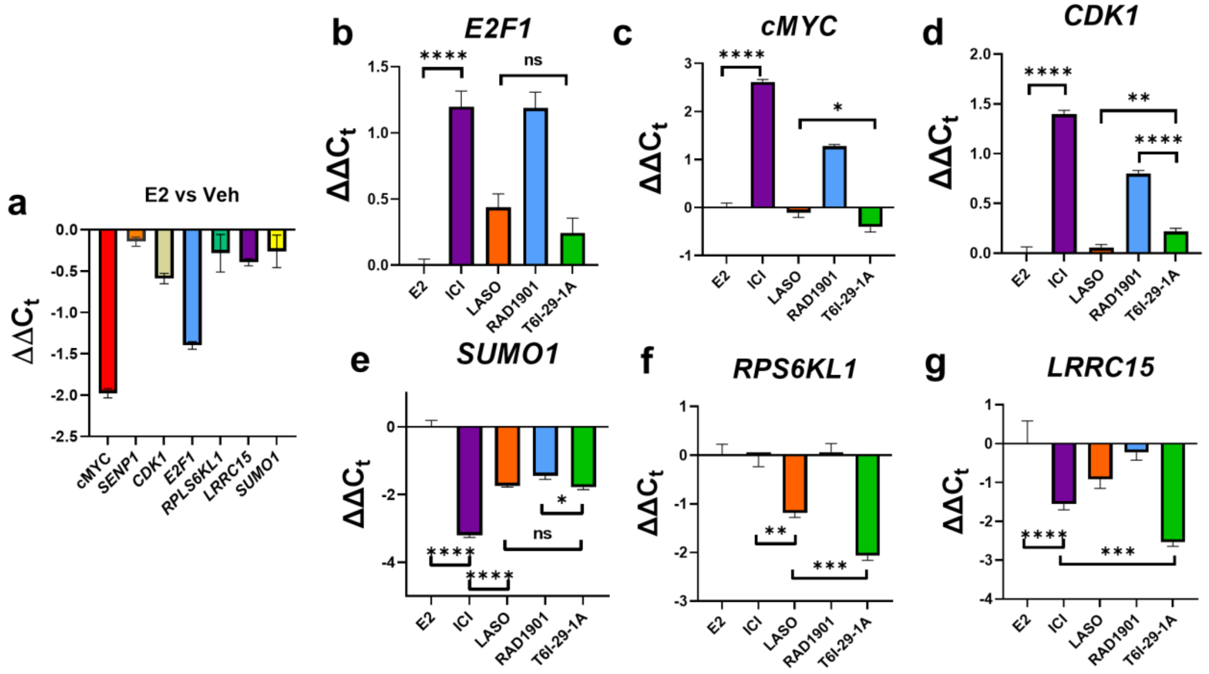

Supplementary Figure 12: Induction of ERα target gene expression in MCF7 WS:8 breast cancer cells. $\Delta\Delta C_t = \Delta C_t(E2) - \Delta C_t(veh)$ (A). The effect of T6I-29-1A on the transcription E2F1 (B), cMYC (C), CDK1 (D), SUMO1 (E), RPSKL1 (F), and LRRC15 (G) in MCF7 WS:8 cells. Significance was determined by unpaired t-test where * $p < 0.05$, ** $p < 0.005$, ***$p < 0.0005$, **** $p < 0.00005$, ns = not significant.

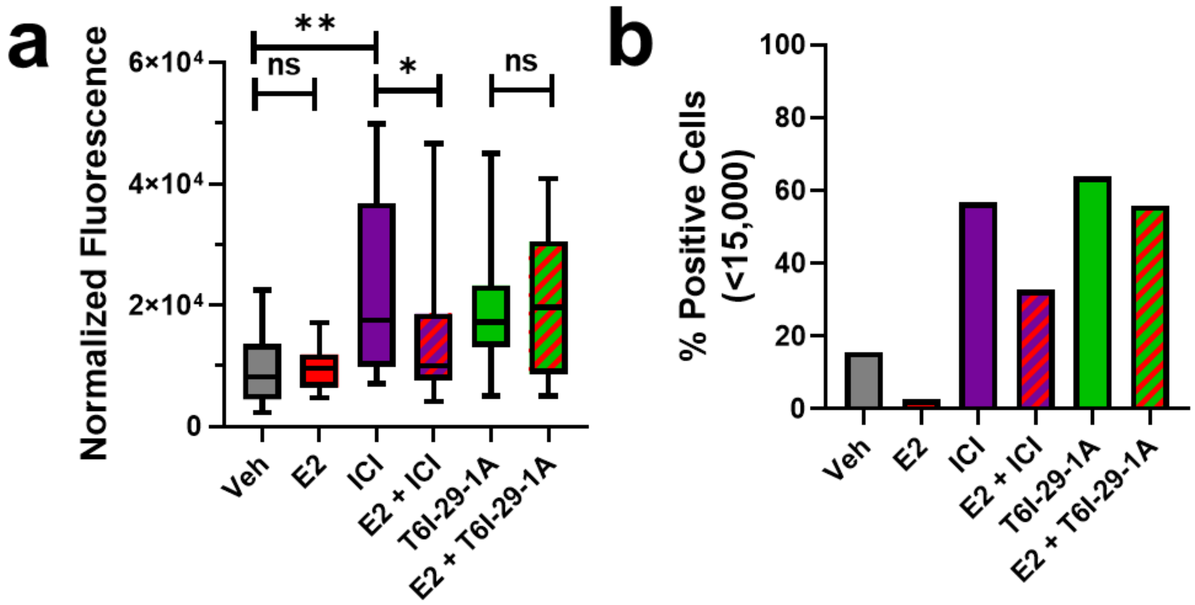

Supplementary Figure 13: Quantification of nuclear SUMO1 immunofluorescence (IF) in T47D breast cancer cells. A) Green fluorescence (SUMO signal) per cell. Significance determined by unpaired t-test where ns = not significant, * $p < 0.05$, ** $p < 0.005$. B) Percent positive cells (those above a threshold of 15,000 relative fluorescence units) in each treatment condition.

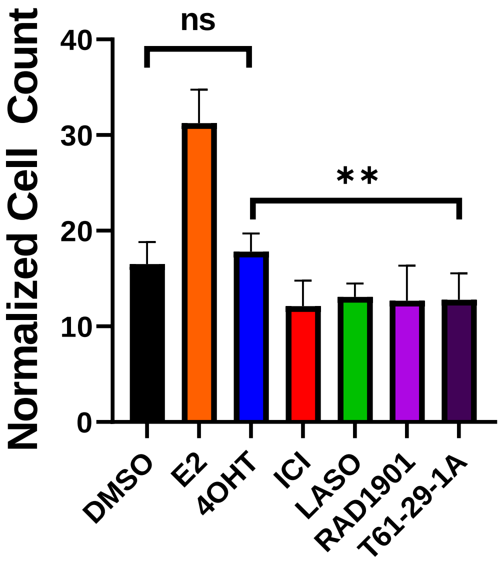

Supplementary Figure 14: Effect of E2 and antiestrogens on Ishikawa proliferation after 72 hours treatment. Cell count is normalized to starting cell count per well. Data are the mean of 2 independent replicates (6 technical replicates total) ± std. dev., ns = not significant, ** $p < 0.005$ as determined by unpaired t-test.

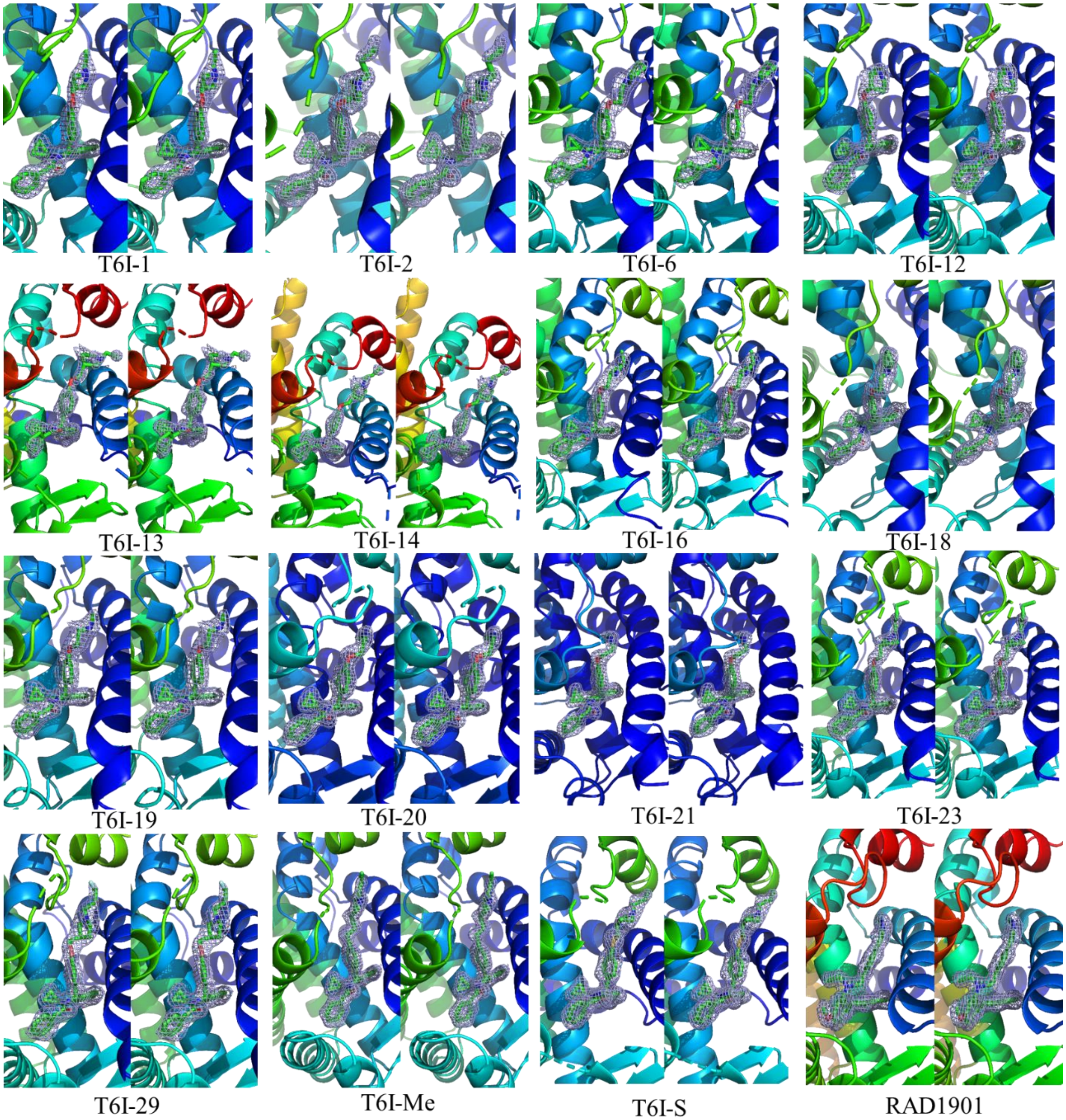

Supplementary Figure 15: Stereo images of difference density maps (2mFo-DFc) of each ligand in the hormone binding pocket for every x-ray co-crystal structure, maps are shown at 1.5 σ.

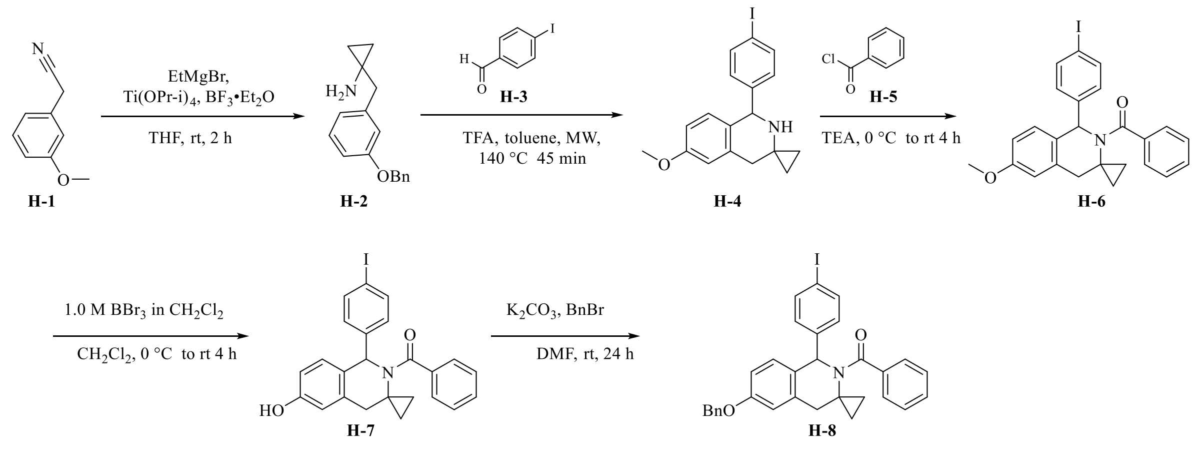

Supplementary Figure 16: Synthetic scheme for intermediate H-8.

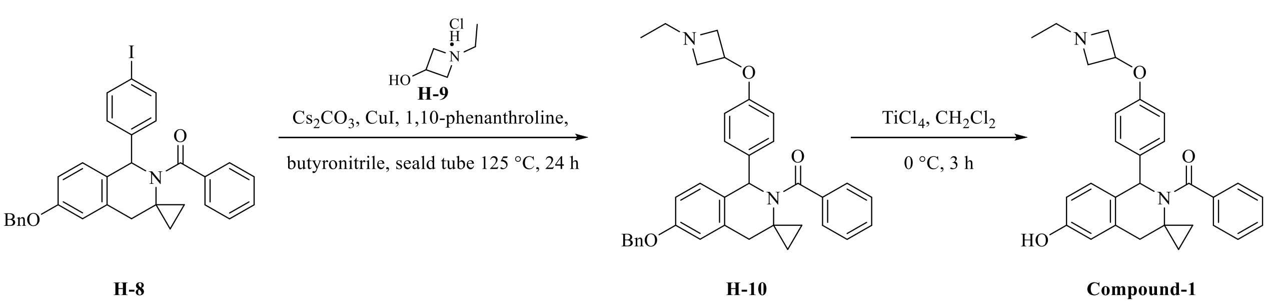

Supplementary Figure 17: Synthetic scheme for compound-1 (T6I-1).

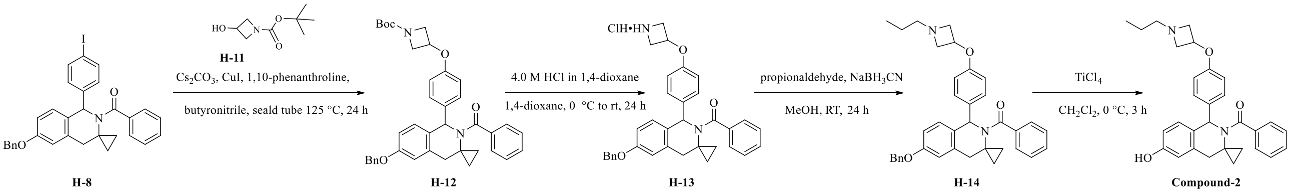

Supplementary Figure 18: Synthetic scheme for compound-2 (T6I-2).

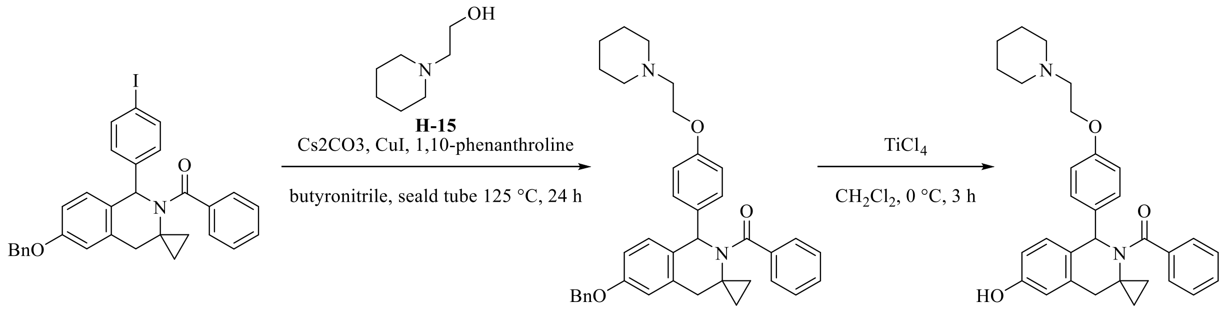

H-8 **H-16** **Compound-6**

Supplementary Figure 19: Synthetic scheme for compound-6 (T6I-6).

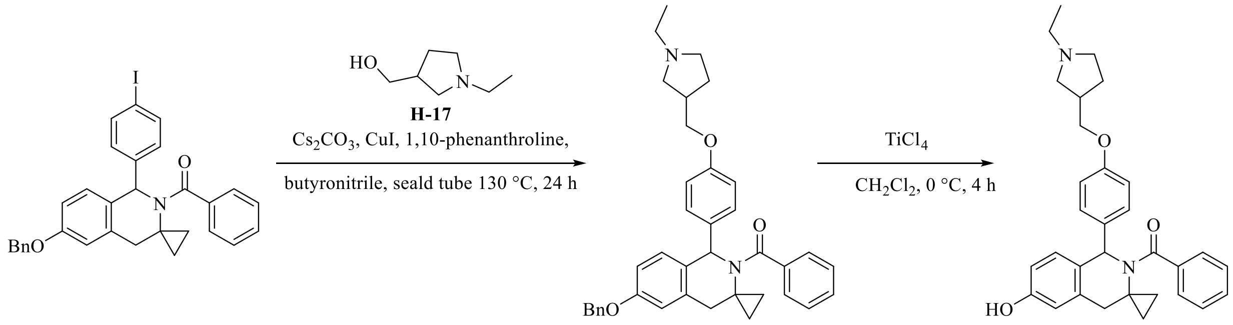

H-8 **H-18** **Compound-13**

Supplementary Figure 20: Synthetic scheme for compound-13 (T6I-13).

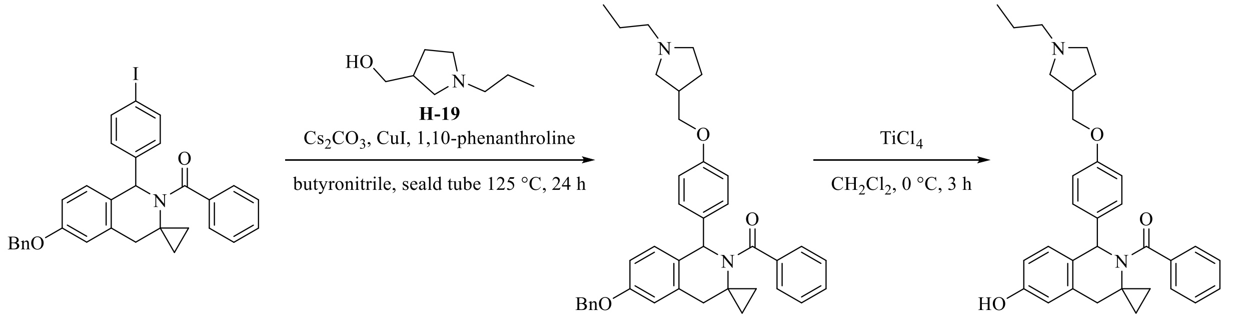

H-8 **H-20** **Compound-14**

Supplementary Figure 21: Synthetic scheme for compound-14 (T6I-14).

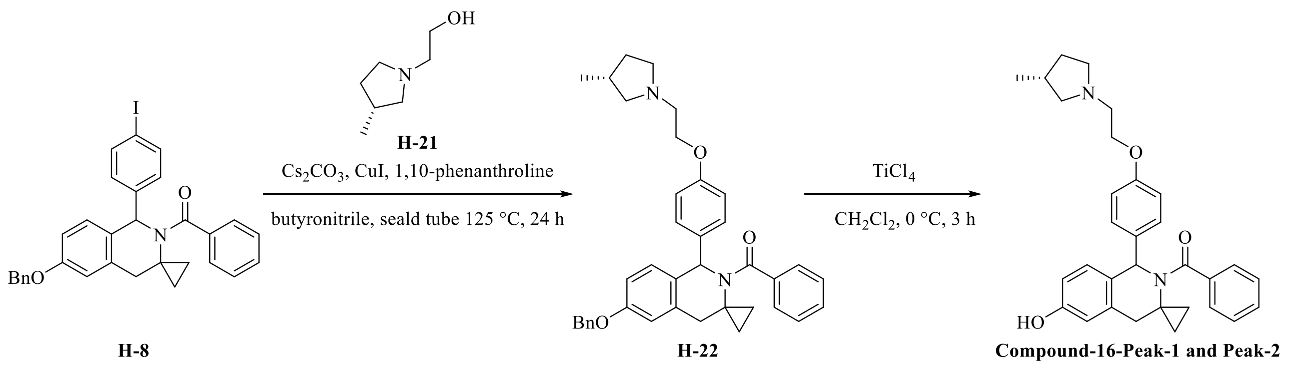

Supplementary Figure 22: Synthetic scheme for compound-16 (T6I-16).

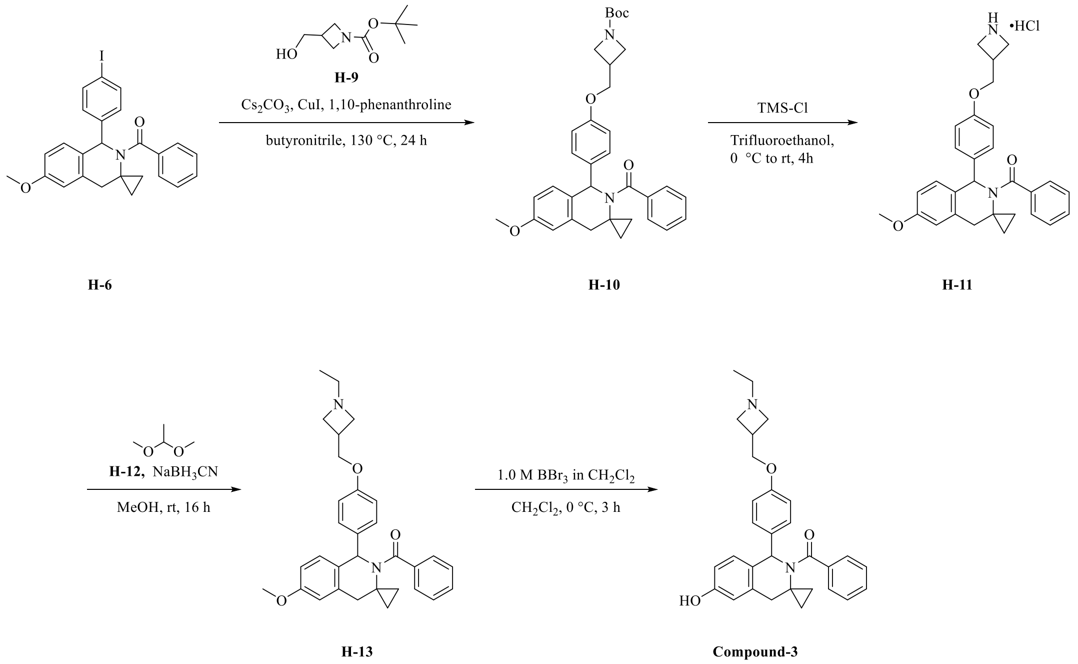

Supplementary Figure 23: Synthetic scheme for compound-3 (T6I-3).

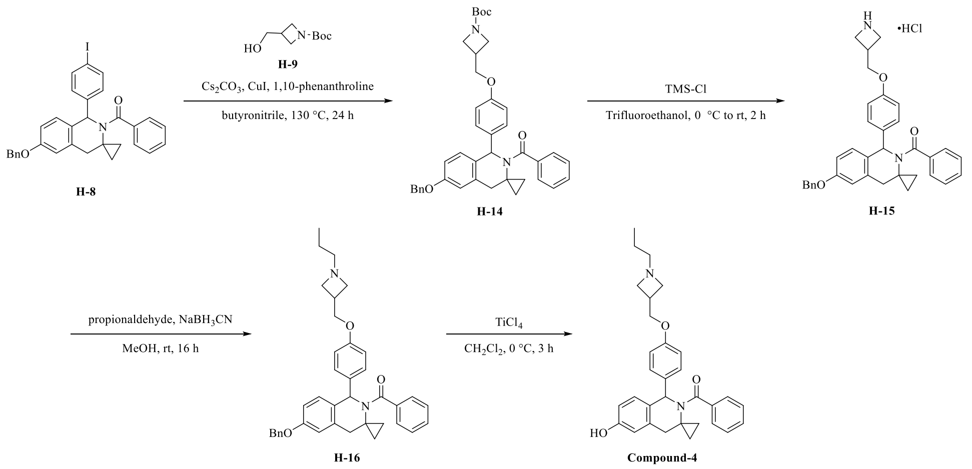

Supplementary Figure 24: Synthetic scheme for compound-4 (T6I-4).

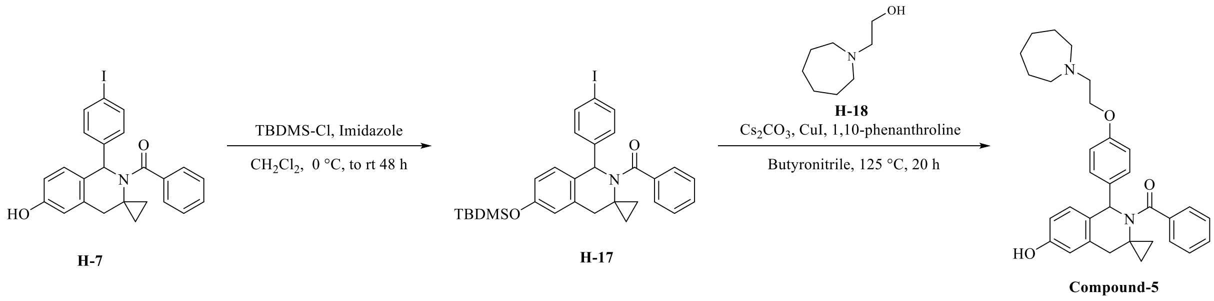

Supplementary Figure 25: Synthetic scheme for compound-5 (T6I-5).

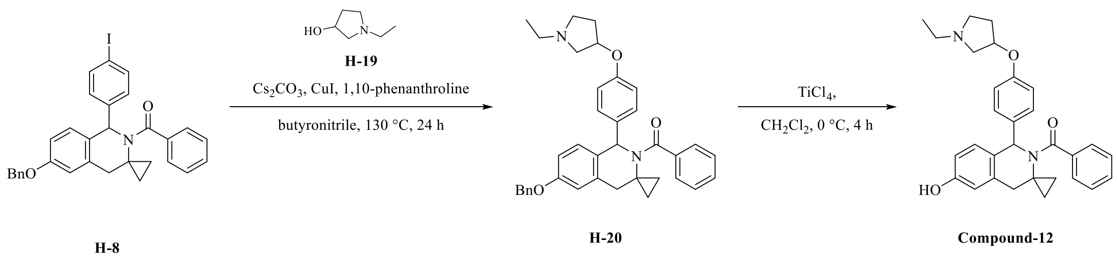

Supplementary Figure 26: Synthetic scheme for compound-12 (T6I-12).

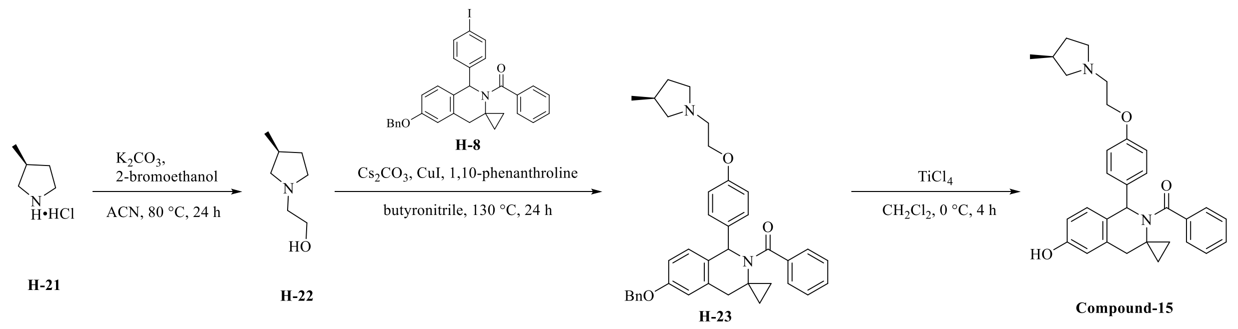

Supplementary Figure 27: Synthetic scheme for compound-15 (T6I-15).

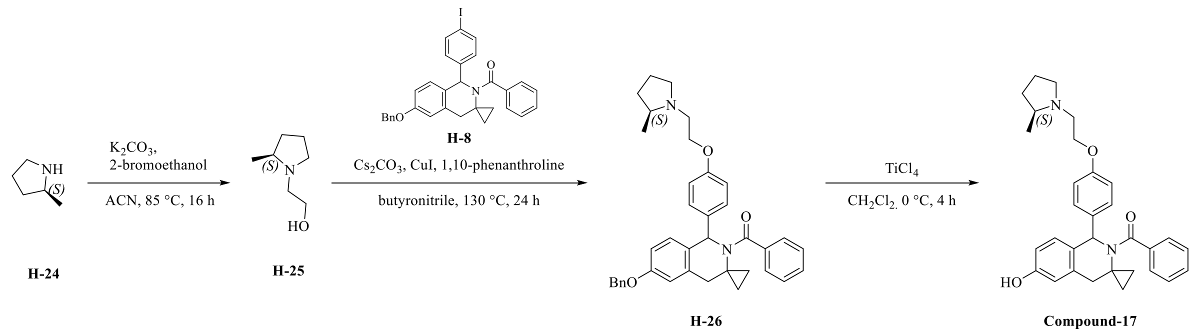

Supplementary Figure 28: Synthetic scheme for compound-17 (T6I-17).

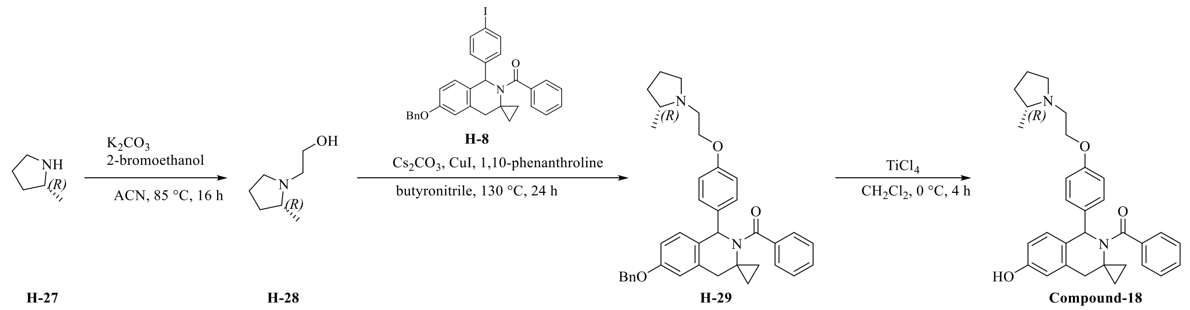

Supplementary Figure 29: Synthetic scheme for compound-18 (T6I-18).

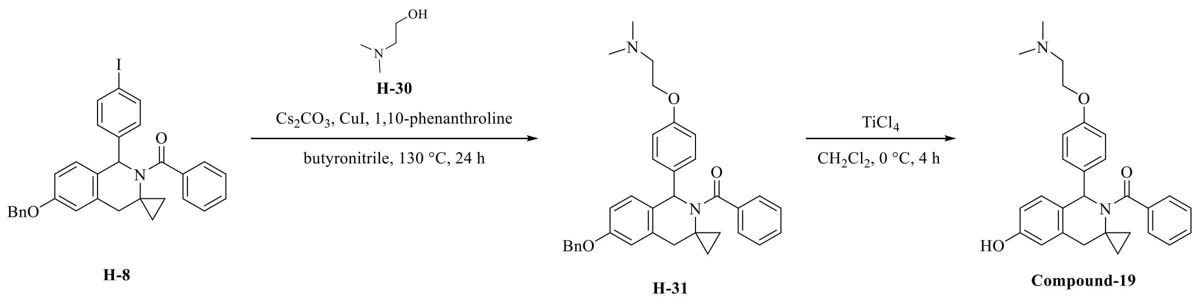

Supplementary Figure 30: Synthetic scheme for compound-19 (T6I-19).

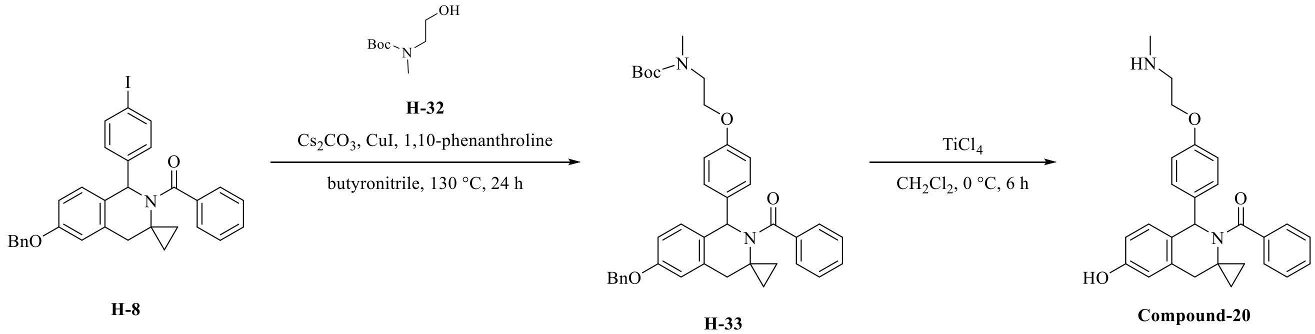

Supplementary Figure 31: Synthetic scheme for compound-20 (T6I-20).

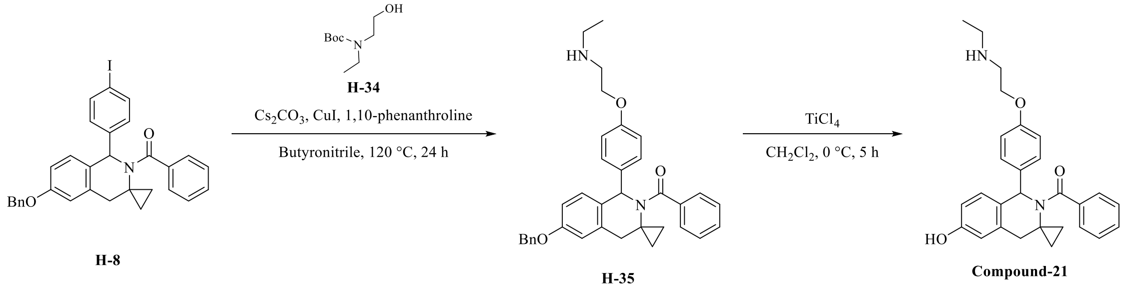

Supplementary Figure 32: Synthetic scheme for compound-21 (T6I-21).

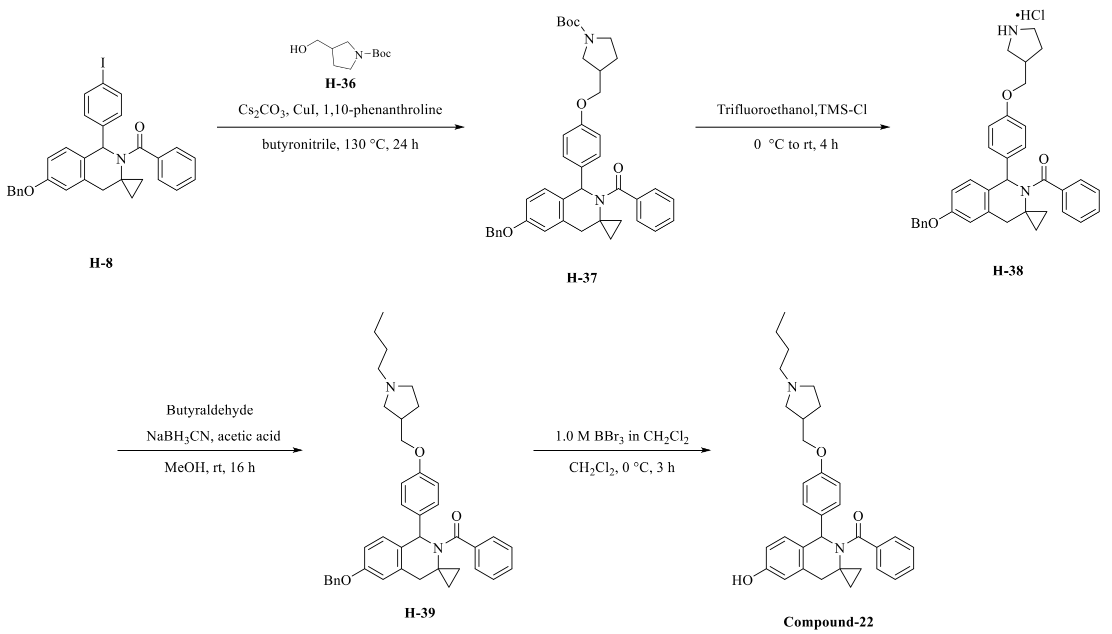

Supplementary Figure 33: Synthetic scheme for compound-22 (T6I-22).

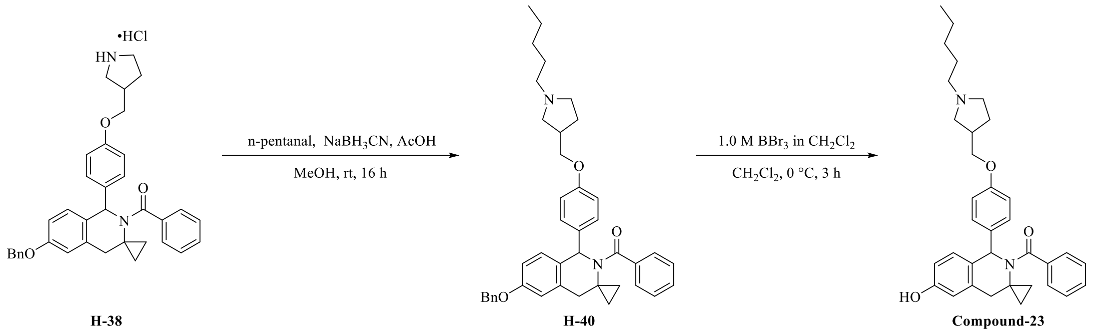

Supplementary Figure 34: Synthetic scheme for compound-23 (T6I-23).

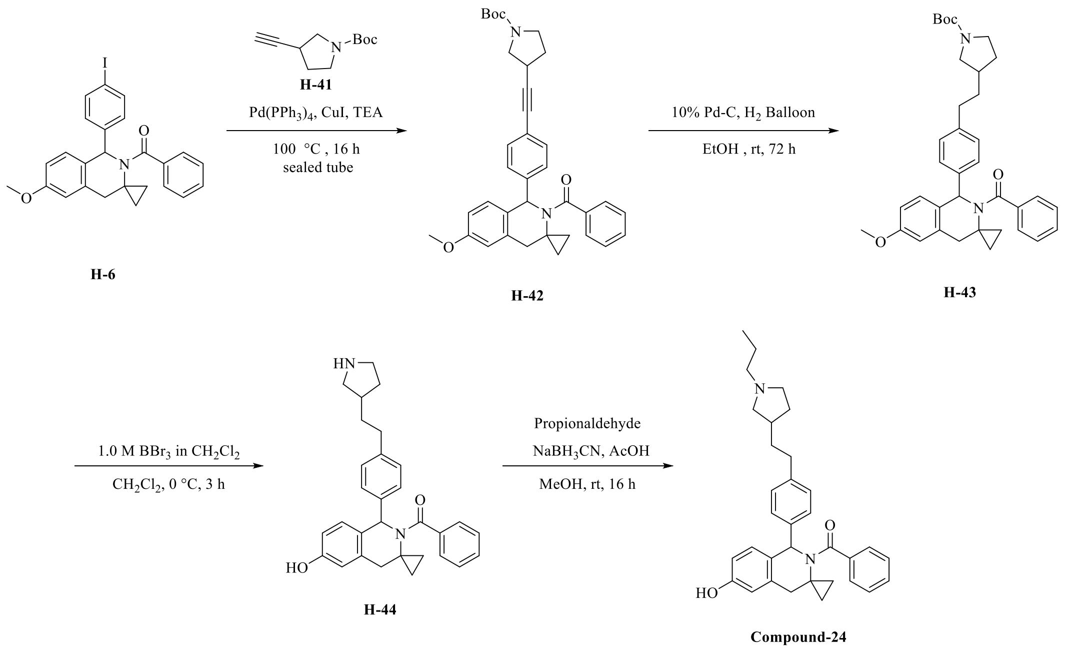

Supplementary Figure 35: Synthetic scheme for compound-24 (T6I-Me).

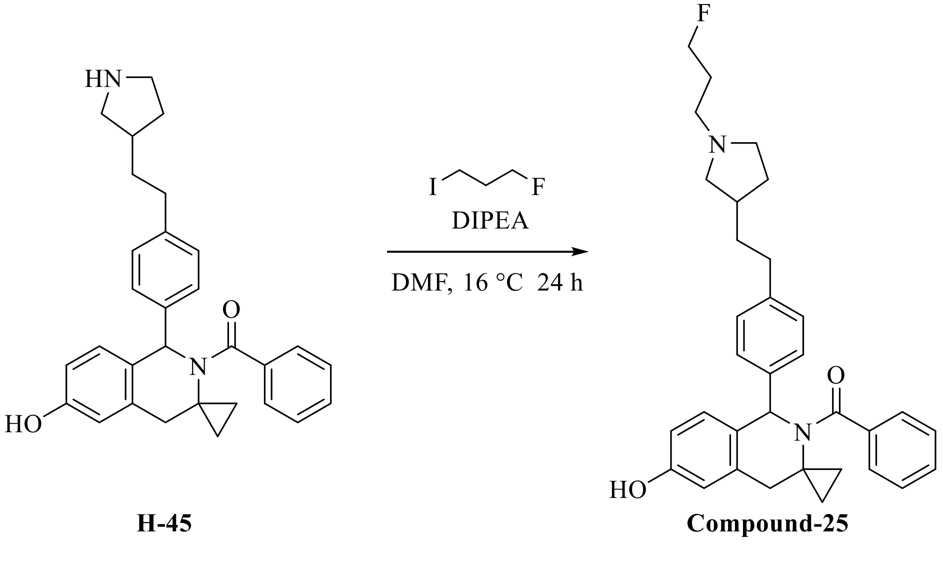

Supplementary Figure 36: Synthetic scheme for compound-25 (T6I-25).

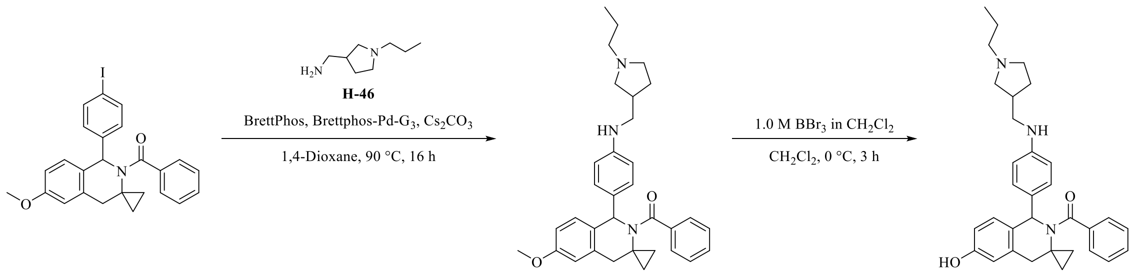

H-6 H-47 Compound-26

Supplementary Figure 37: Synthetic scheme for compound-26 (T6I-NH).

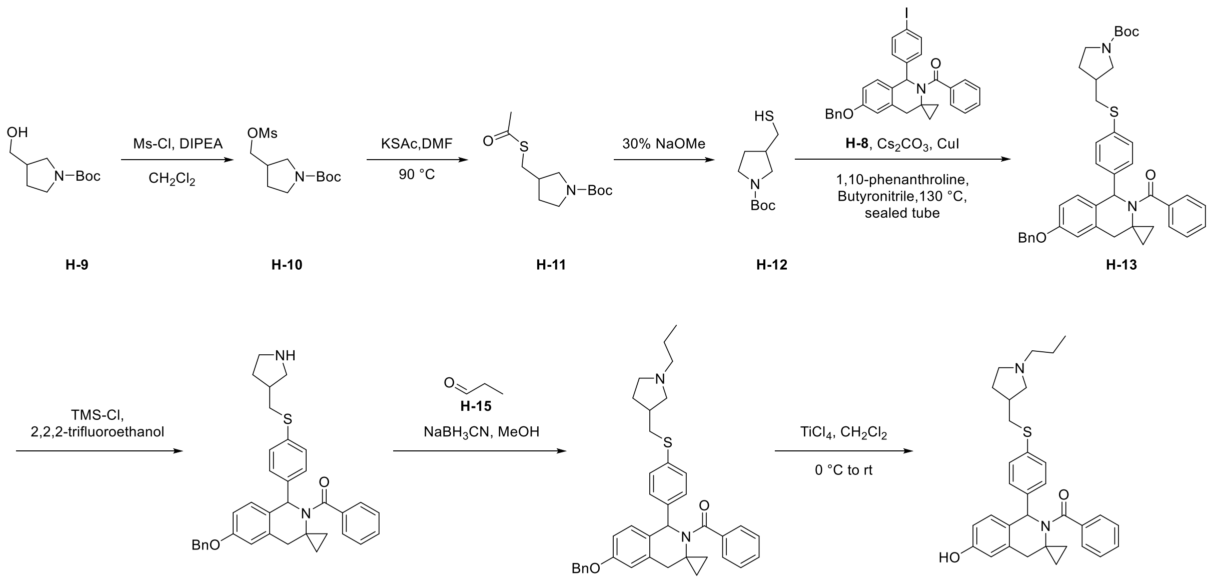

H-14 H-16 Compound- 27

Supplementary Figure 38: Synthetic scheme for compound-27 (T6I-27).

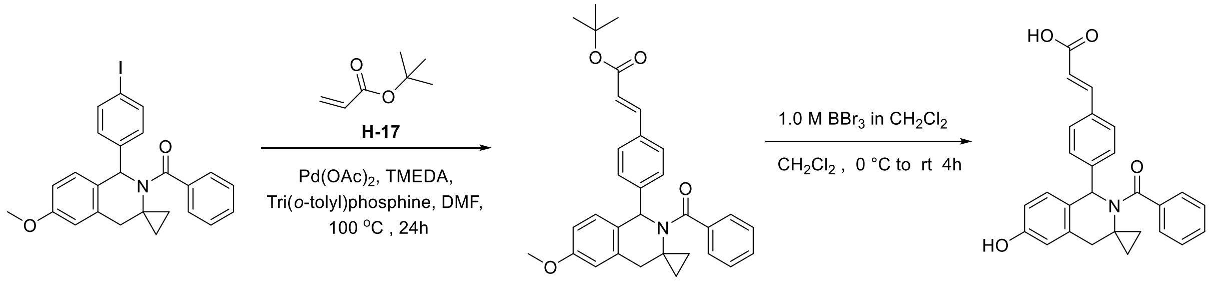

H-6 H-18 Compound-28

Supplementary Figure 39: Synthetic scheme for compound-28 (T6I-28).

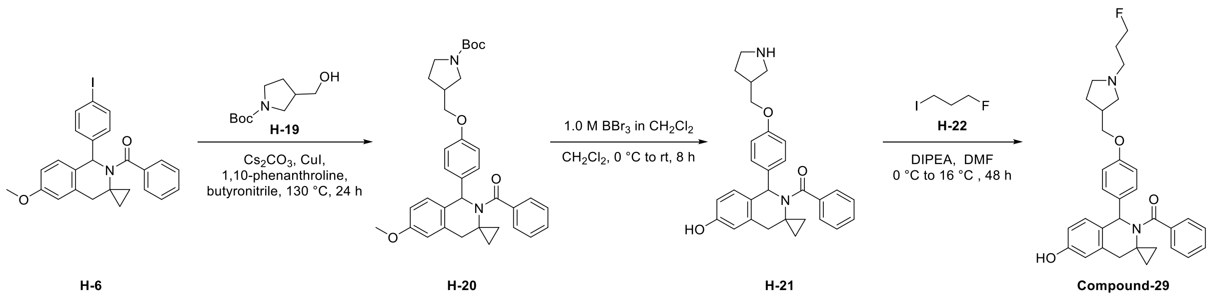

Supplementary Figure 40: Synthetic scheme for compound-29 (T6I-29).

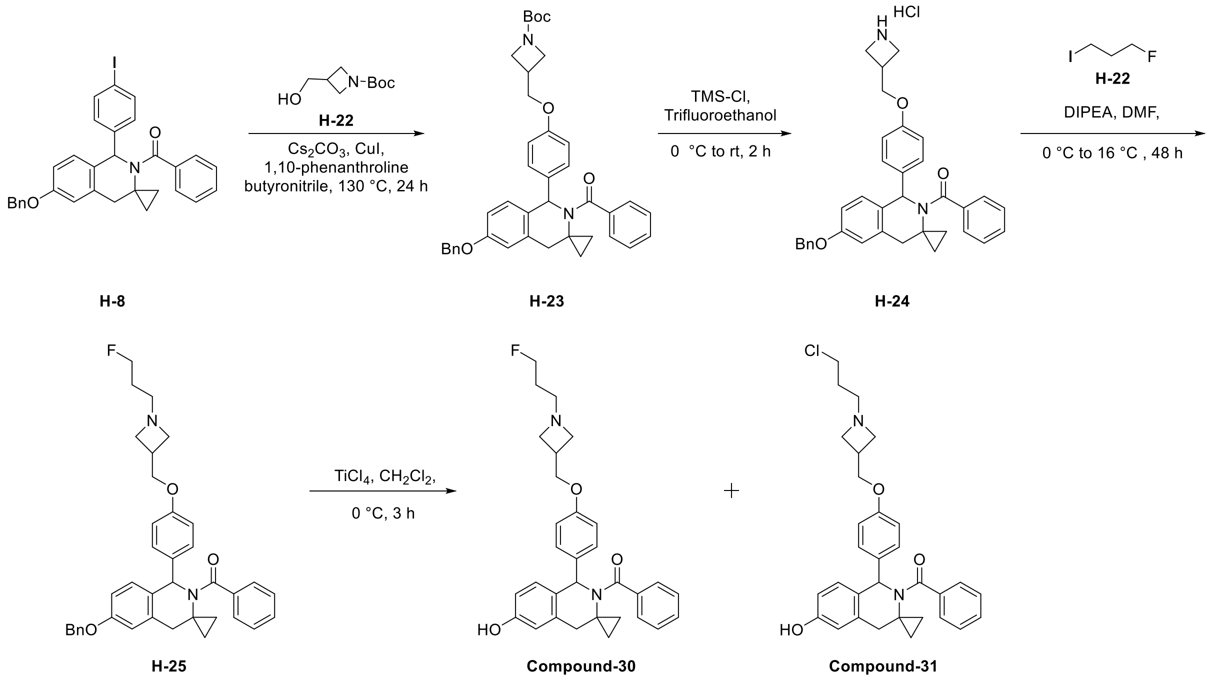

Supplementary Figure 41: Synthetic scheme for compounds-30 and 31 (T6I-4A and 4B).

SUPPLEMENTARY TABLES

Supplementary Table 1: Mean + std. dev. for AP1 assay (*n* = 3-9).

**Treatment**	**AP1 Induction**
E2	0.99 ± 0.05
4OHT	0.67 ± 0.12
ICI	0.25 ± 0.08
Laso	0.51 ± 0.16
RAD1901	0.28 ± 0.08
T6I-1	0.30 ± 0.07
T6I-2	0.25 ± 0.08
T6I-3	0.34 ± 0.07
T6I-4	0.24 ± 0.07
T6I-4A	0.69 ± 0.16
T6I-4B	0.61 ± 0.10
T6I-5	0.57 ± 0.14
T6I-6	0.43 ± 0.14
T6I-12	0.41 ± 0.08

T6I-13-1	0.66 ± 0.17
T6I-14-1	0.55 ± 0.09
T6I-15-1	0.55 ± 0.12
T6I-16-1	0.35 ± 0.09
T6I-17	0.71 ± 0.20
T6I-18	0.69 ± 0.10
T6I-19	0.36 ± 0.03
T6I-20	0.30 ± 0.05
T6I-21	0.46 ± 0.04
T6I-22	0.55 ± 0.06
T6I-23	0.44 ± 0.08
T6I-Me	0.57 ± 0.08
T6I-NH	0.50 ± 0.09
T6I-SH	0.42 ± 0.07
T6I-29	0.63 ± 0.08
T6I-29-1A	0.54 ± 0.07
T6I-29-1B	0.32 ± 0.05

T6I-29-2A	0.33 $\pm$ 0.05
T6I-29-2B	0.56 $\pm$ 0.09

Supplementary Table 2: X-ray Crystal Structure Data Collection and Refinement for Ligands in Complex with ERα LBD (Highest Resolution Shell).

	**T6I-1**	**T6I-2**	**T6I-6**	**T6I-12**
PDB ID	8DU6	8DU8	8DU9	8DUB
Data Collection				
Space Group	C2	C2	C2	C2
Cell dimensions				
a, b, c (Å)	102.28, 58.37, 87.66	102.66, 57.67, 87.43	102.25, 58.08, 87.57	102.52, 57.89, 87.72
α, β, γ (°)	90.00, 103.12, 90.00	90.00, 103.20, 90.00	90.00, 103.00, 90.00	90.00, 103.09, 90.00
Resolution (Å)	2.10	1.47	2.50	1.84
$CC^{1/2}$	0.985 (0.558)	0.998 (0.92)	0.980 (0.589)	0.984 (0.606)
$I/\sigma I$	2.44 (at 2.10 Å)	2.40 (at 1.47 Å)	5.81 (at 2.51 Å)	1.83 (at 1.84 Å)
Completeness	95.6 (86.5)	98.3 (88.0)	99.9 (100)	99.8 (100)
Redundancy	3.3	3.7	3.6	3.7
Refinement				
Resolution Range (Å)	49.97 – 2.1	29.13 – 1.47	49.81 – 2.50	45.52 – 1.84
Number of Reflections	28,464	83,028	17,470	43,269
R_{work}/R_{free}	18.9/23.9	16.9/19.4	21.0/25.8	17.5/22.1
No. Atoms	3,875	4,306	3,577	3,926
Water Molecules	199	541	81	316
Ligand Molecules	2	2	2	2
R.m.s. deviations				
Bond Lengths (Å)	0.003	0.018	0.004	0.018
Bond Angles (°)	0.61	0.74	0.73	1.662

	**T6I-13**	**T6I-14-1**	**T6I-16-1**	**T6I-18**
PDB ID	8DUC	8DUD	8DUG	8DUH
Data Collection				
Space Group	P6522	P6522	C2	C2
Cell dimensions				
a, b, c (Å)	58.60, 58.60, 276.75	58.77, 58.77, 276.78	103.13, 57.22, 87.69	102.72, 57.28, 87.52
α, β, γ (°)	90.00, 90.00, 120.00	90.00, 90.00, 120.00	90.00, 103.89, 90.00	90.00, 103.49, 90.00
Resolution (Å)	1.70	1.81	2.20	1.90
$CC^{1/2}$	0.99 (0.58)	0.99 (0.52)	0.998 (0.58)	0.991 (0.502)
$I/\sigma I$	1.68 (at 1.70 Å)	1.95 (at 1.81 Å)	1.70 (at 2.20 Å)	0.97 (at 1.89 Å)
Completeness	99.9 (98.2)	99.1 (84.6)	99.7 (96.5)	97.4 (82.7)
Redundancy	18.9	14.9	3.6	3.6
Refinement				

Resolution Range (Å)	47.65 – 1.70	47.77 – 1.81	45.32 – 2.20	45.26 – 1.90
R_{work}/R_{free}	22.2/26.2	21.2/25.5	19.1/25.0	18.6/23.5
Number of Reflections	32,444	25,250	25,439	37,736
No. Atoms	2,029	1,998	3,655	3,888
Water Molecules	239	179	141	289
Ligand Molecules	1	1	2	2
Bond Lengths (Å)	0.01	0.01	0.04	0.009
Bond Angles (°)	1.32	1.24	0.61	1.12

	**T6I-19**	**T6I-20**	**T6I-21**	**T6I-23**
PDB ID	8DUI	8DUK	8DUS	8DV5
Data Collection				
Space Group	C2	C2	C2	C2
Cell dimensions				
a, b, c (Å)	102.97, 57.49, 87.57	102.03, 57.66, 174.52	102.04, 57.67, 259.87	101.93, 57.34, 87.14
α, β, γ (°)	90.00, 103.77, 90.00	90.00, 102.51, 90.00	90.00, 100.08, 90.00	90.00, 103.09, 90.00
$CC^{1/2}$	0.998 (0.521)	0.998 (0.624)	0.993 (0.516)	0.999 (0.547)
$I/\sigma I$	2.35 (at 2.04 Å)	1.72 (at 1.70 Å)	1.59 (at 1.90 Å)	1.89 (at 1.86 Å)
Completeness	0.998 (0.95)	0.994 (0.942)	0.988 (0.872)	0.998 (0.982)
Redundancy	3.7	3.4	3.0	3.7
Refinement				
Resolution Range (Å)	45.42 – 2.04	49.91 – 1.7	49.91 – 1.90	45.15 – 1.85
Number of Reflections	38,034	106,232	102,498	41,967
R_{work}/R_{free}	18.8/22.6	23.8 /26.8	28.5/32.7	18.4/21.3
No. Atoms	3,757	8,138	11,368	3,986
Water Molecules	199	656	489	301
Ligand Molecules	2	4	6	2
Bond Lengths (Å)	0.04	0.01	0.01	0.06
Bond Angles (°)	0.92	1.22	1.31	1.017

	**T6I-Me**	**T6I-S**	**T6I-29**	**RAD1901**
PDB ID	8DV7	8DV8	8DVB	7TE7
Data Collection				
Space Group	C2	C2	C2	C2
Cell dimensions				
a, b, c (Å)	102.29, 57.78, 87.41	103.11, 58.13, 87.64	103.10, 56.34, 87.35	58.183, 58.183, 277.24
α, β, γ (°)	90.00, 103.10, 90.00	90.00, 102.79, 90.00	90.00, 103.25, 90.00	90.00, 90.00, 120.00

Resolution	1.59	1.70	2.19	1.85
$CC^{1/2}$	0.997 (0.524)	0.999 (0.876)	0.999 (0.524)	0.998 (0.510)
$I/\sigma I$	1.93 (at 1.97 Å)	1.76 (at 1.70 Å)	1.23 (at 2.18 Å)	7.26 (at 1.86 Å)
Completeness	0.951 (0.70)	0.996 (0.975)	0.999 (0.96)	0.993 (0.87)
Redundancy	3.6	3.7	3.7	3.7
Refinement				
Resolution Range (Å)	41.13 – 1.59	41.41 – 1.70	49.12 – 2.19	50 – 1.85
Number of Reflections	63,596	54,985	19,241	27,286
R_{work}/R_{free}	16.7/20.8	18.7/21.1	19.2/24.2	21.9/24.6
No. Atoms	4,195	4,000	3,867	1,841
Water Molecules	442	466	137	250
Ligand Molecules	2	2	2	1
Bond Lengths (Å)	0.016	0.007	0.004	0.005
Bond Angles (°)	1.56	1.17	0.931	0.847

Supplementary Table 3: HPLC Method Gradient.

Time (min)	Flow (mL/min)	%A	%B
0	1.0	95.0	5.0
2	1.0	95.0	5.0
8	1.0	30.0	70.0
10	1.0	5.0	95.0
12	1.0	5.0	95.0
12.1	1.0	95.0	5.0
15	1.0	95.0	5.0

Supplementary Table 4: Prep HPLC Gradient.

Time (min)	Flow (mL/min)	%A	%B
0	30.0	90.0	10
1	30.0	90.0	10
10	30.0	50.0	50
15	30.0	20.0	80
15.5	30.0	5.0	95.0
17.5	30.0	5.0	95.0
18.0	30.0	90.0	10.0
20.0	30.0	90.0	10.0

Supplementary Table 5: Preparative SFC Method Conditions.

Column	Chiralcel OX-H (250mmX21mm) 5µ
Total Flow	85g-40%
Mobile Phase A	51g/min (CO_2)
Mobile Phase B	34 mL/min (0.3% NH_3 in Methanol)
ABPR	100 bar
Detection	UV@220 nm
Run time	20 min

Stocking time	12 min
Loading	10 mg/injection
Dilution	Methanol (100 %)

Supplementary Table 6: Analytical SFC Method Conditions (Method-B):

Column	Chiralcel OX-H(150mmX4.6mm) 5µ
Total Flow	4g-40%
Mobile Phase A	2.4g/min (CO_2)
Mobile Phase B	1.6 mL/min (0.3% DEA in Methanol)
ABPR	1500 psi
Column Temp	40 degree
Detection	UV@220 nm
Run time	15 min
Dilution	Methanol (100 %)
